# Accuracy of serological tests for COVID-19: A systematic review and meta-analysis

**DOI:** 10.3389/fpubh.2022.923525

**Published:** 2022-12-16

**Authors:** Xiaoyan Zheng, Rui hua Duan, Fen Gong, Xiaojing Wei, Yu Dong, Rouhao Chen, Ming yue Liang, Chunzhi Tang, Liming Lu

**Affiliations:** ^1^School of Rehabilitation Sciences, Southern Medical University, Guangzhou, China; ^2^First Clinical Medical College, Guangzhou University of Chinese Medicine, Guangzhou, China; ^3^Medical College of Acupuncture-Moxibustion and Rehabilitation, Guangzhou University of Chinese Medicine, Guangzhou, China

**Keywords:** serological tests, COVID-19, systematic review, meta-analysis, RT–PCR

## Abstract

**Objective:**

To determine the diagnostic accuracy of serological tests for coronavirus disease-2019 (COVID-19).

**Methods:**

PubMed, Embase and the Cochrane Library were searched from January 1 2020 to September 2 2022. We included studies that measured the sensitivity, specificity or both qualities of a COVID-19 serological test and a reference standard of a viral culture or reverse transcriptase polymerase chain reaction (RT–PCR). The risk of bias was assessed by using quality assessment of diagnostic accuracy studies 2 (QUADAS-2). The primary outcomes included overall sensitivity and specificity, as stratified by the methods of serological testing [enzyme-linked immunosorbent assays (ELISAs), lateral flow immunoassays (LFIAs) or chemiluminescent immunoassays (CLIAs)] and immunoglobulin classes (IgG, IgM, or both). Secondary outcomes were stratum-specific sensitivity and specificity within the subgroups, as defined by study or participant characteristics, which included the time from the onset of symptoms, testing *via* commercial kits or an in-house assay, antigen target, clinical setting, serological kit as the index test and the type of specimen for the RT–PCR reference test.

**Results:**

Eight thousand seven hundred and eighty-five references were identified and 169 studies included. Overall, we judged the risk of bias to be high in 47.9 % (81/169) of the studies, and a low risk of applicability concerns was found in 100% (169/169) of the studies. For each method of testing, the pooled sensitivity of the ELISAs ranged from 81 to 82%, with sensitivities ranging from 69 to 70% for the LFIAs and 77% to 79% for the CLIAs. Among the evaluated tests, IgG (80–81%)-based tests exhibited better sensitivities than IgM-based tests (66–68%). IgG/IgM-based CLIA had the highest sensitivity [87% (86–88%)]. All of the tests displayed high specificity (97–98%). Heterogeneity was observed in all of the analyses. The detection of nucleocapsid protein (77–80%) as the antigen target was found to offer higher sensitivity results than surface protein detection (66–68%). Sensitivity was higher in the in-house assays (78–79%) than in the commercial kits (47–48%).

**Conclusion:**

Among the evaluated tests, ELISA and CLIA tests performed better in terms of sensitivity than did the LFIA. IgG-based tests had higher sensitivity than IgM-based tests, and combined IgG/IgM test-based CLIA tests had the best overall diagnostic test accuracy. The type of sample, serological kit and timing of use of the specific tests were associated with the diagnostic accuracy. Due to the limitations of the serological tests, other techniques should be quickly approved to provide guidance for the correct diagnosis of COVID-19.

## Introduction

Coronavirus disease 2019 (COVID-19), which is caused by severe acute respiratory syndrome coronavirus-2 (SARS-CoV-2), has affected 219 countries and territories, with 614,385,693 confirmed cases; additionally, 6,522,600 deaths have been reported by the World Health Organization last update 30 September 2022. Accurate and rapid diagnostic tests are critical in achieving the global control of COVID-19. There are two main diagnostic tests for COVID-19: molecular tests that detect viral RNA, and serological tests that detect anti-SARS-CoV-2 immunoglobulin (1). Reverse transcription polymerase chain reaction (RT–PCR) is the gold standard diagnostic test recommended by the current guidelines (2). However, RT–PCR exhibits its own limitations, including inappropriate specimen collection techniques, viral load time since the time of exposure (3) and the source of the specimen, which can contribute to false-negative test results (4). The rates of false-positive RT–PCR performance on the day of the onset of symptoms are 100% but decrease to 38% 5 days later (5). Serological testing is a blood test that can detect specific antibodies against COVID-19, including immunoglobulin M (IgM), IgG and IgA antibodies. Serological tests have been developed as supplementary diagnostic methods, as they can take several days or weeks to develop antibodies after viral exposure; therefore, they can provide information about recent or prior infections (1). As such, serological tests can be used as surveillance tools to better understand the overall infection rate in different regions and populations wherein quantitative PCR assays are not available or are delayed (6). Given the importance of serological tests in combating COVID-19, systematic reviews and meta-analyses that aim to summarize the accuracy parameters of serological tests and to investigate whether they are sufficiently specific or sensitive to achieve their role in practice are urgently needed.

Although some studies have compared pooled sensitivities and specificities of serological test methods, as well as identifying study and patient characteristics (7–10), high-quality evidence supporting the use of antibody tests for COVID-19 in practice is missing, due to a fast-growing field; additionally, ongoing updates of this systematic review will be implemented (11). Therefore, we conducted a systematic review and meta-analysis to assess the diagnostic accuracy of serological tests for COVID-19 infection. We aimed to understand the global serological tests of coronavirus with maps and updates on the overall sensitivity and specificity. To reduce variability in the estimates and to enhance generalizability, both sensitivity and specificity were stratified by clinical setting (outpatient vs. inpatient), antigen target, serological kit as the index test and the number of days that elapsed since the onset of symptoms. Analyses on the sensitivity and specificity of the different testing methods were performed to provide scientific guidance for the design and evaluation of vaccines and therapeutic antibodies in the future (1).

## Methods

### Search strategy

This meta-analysis was conducted according to the Preferred Reporting Project for Systematic Reviews and Meta-Analyses (PRISMA) guidelines (12) and recommends best practices (13). We searched the PubMed, Embase and the Cochrane Library. The search terms used were (SARS-CoV-2 OR Coronavirus disease 2019 OR COVID-19) AND (IgM OR IgG). The searches ends September 2, 2022, with no restrictions on language. The detailed search strategy is in Supplementary material.

### Types of studies

We included studies that met the following criteria. (1) Eligible studies, including randomized trials, cohort studies, or case-control studies, and case series reporting sensitivity, specificity, or both qualities of serological testing for COVID-19. (2) Studies evaluating any test that detects antibodies to SARS-CoV-2, including laboratory-based methods and tests designed for use in field therapy. Test methods include: laboratory-based enzyme-linked immunosorbent assay (ELISA) and chemiluminescence immunoassay (CLIA). Rapid diagnostic tests use lateral flow assays (LFIA), including colloidal gold or fluorescently labeled immunochromatographic assays (CGIA or FIA). (3) Serological diagnostic tests not limited to any antibodies, antigens or test methods.

The exclusion criteria were as follows: (1) case reports, review articles and editorials; (2) studies that focus on ineligible populations, such as vaccinated patients and people not infected with the coronavirus.

Three different researchers independently screened literature, extracted data and validated the results. If there is an objection, resolve it by discussion or negotiation with a third researcher.

### Participants

We included studies that recruited people with suspicion of current or previous SARS-CoV-2 infection confirmed by NAT (such as RT PCR or sequencing) or NAT in combination with clinical outcomes.

### Outcomes

The primary outcomes included overall sensitivity and specificity, stratified by serological tests (ELISA, LFIA, and CLIA) and immunoglobulin class (IgG, IgM, or both). Secondary outcomes include layer-specific sensitivity and specificity within subgroups, defined by study or participant characteristics.

### Data extraction and bias assessment

The following data were independently extracted by 2 professional researchers: general study details (authors, year of publication, country of origin, study design, sample size, reagent company, time from symptom onset to index test and clinical setting and whether testing was performed *via* commercial kits or an in-house assay), methods, characteristics and diagnostic test results [true positive (TP), true negative (TN), false-positive (FP), false negative (FN), sensitivity, specificity and accuracy] (7).

Two researchers independently assessed the risk of bias for each study using the Cochrane Collaboration recommended Diagnostic Precision Study Quality Assessment Tool (QUADAS-2) (14). Quadas-2 is a quality assessment tool developed specifically for the systematic evaluation of accuracy studies, covering the following four key areas: patient selection, index test, reference standard and flow and timing. Additionally, each area was divided into low risk, high risk and unclear risk. The tool classifies evidence from observational studies into “low risk of bias,” “unclear” and “high risk of bias” level. If at least 50% of the fields are classified as low bias risk, the overall risk of bias for individual studies is classified as low bias risk; Otherwise, a higher risk of bias is defined (15).

### Statistical analysis

Sensitivity and specificity of calculated estimates for each individual study (based on 2 × 2 contingency tables). All of the results are reported with 95% confidence intervals (CIs). Data are summarized as paired Forest plots. Since different studies have different cutoff values, a two-variable random effects model was used for meta-analysis. A summary receiver operating characteristic (ROC) curve based on TP and FP rates was established to describe the relationship between detection sensitivity and specificity. The area under the curve (AUC) is close to 1, indicating that the test has good diagnostic performance. All of the analyses were performed using Meta-Disc version 1.4.7. Random effects logistic regression model was used to compare the diagnostic accuracy of different antibodies, different antibody detection methods and different antigens. The heterogeneity of the study was determined by summary ROC curves with 95% prediction regions, estimated using bivariate meta-analysis with a test level random effect only, and forest plots. As our models were bivariate, we did not use the *I*^2^ statistic.

In the subgroup analysis, to assess pre-specified variables as potential determinants of diagnostic accuracy, we collected samples at times associated with symptom onset (at week 1, week 2, at week 3, or after week 3); Depending on the antigen target [surface protein (S), nucleocapsid protein (N), surface and nucleocapsid protein], the test is performed using a commercial kit or an internal test; Clinical institutions (inpatient, outpatient, inpatient, outpatient only); Serological kits as indicative tests (using commercial kits or in-house tests); and the type of specimen used for RT PCR reference testing (nasopharyngeal or sputum, saliva or oral, throat and pharyngeal). In these analyses, we pooled data according to the test method (ELISA, LFIA and CLIA) and immunoglobulin class (IgM, IgG or both).

### Patient and public involvement

Patients were not involved in the formulation of study questions or outcome measurements, the conduct of the study or the preparation of the manuscript (7).

## Results

### Description of included studies

**Figure 1:**
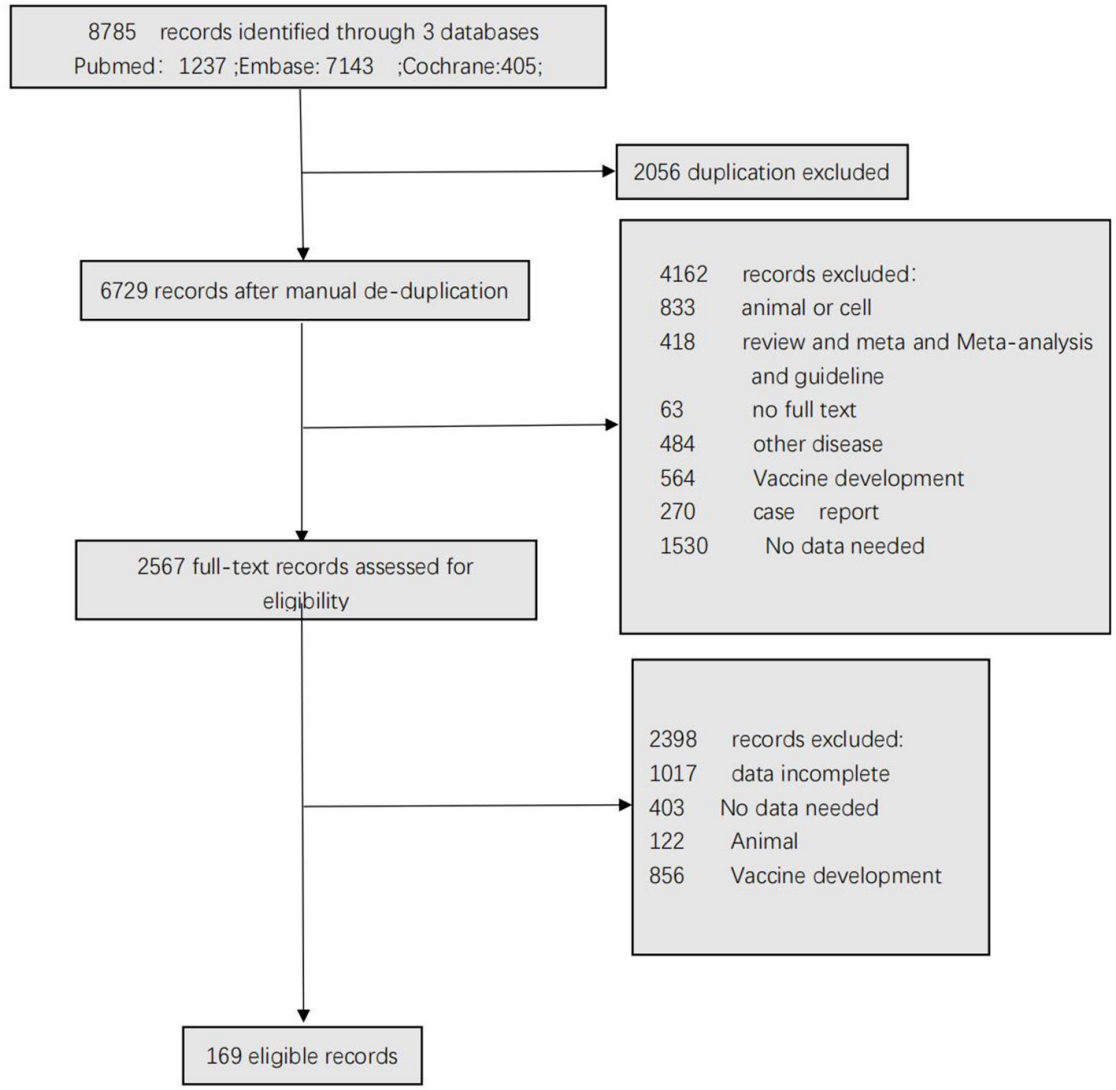
Study selection.

Figure 1 shows the selection of the studies. A total of 8,785 articles were identified after the removal of duplicate articles. Of these articles, 2,056 articles were excluded during the screening phase (title and abstract reading), with 6,560 records being fully appraised. Finally, 169 articles met the inclusion criteria.

### Participant characteristics

Table 1 summarizes the studies by test method; the sum of the number of studies exceeded 169 because some studies evaluated more than one method. For example, a study that assessed 2 LFIAs and 3 ELISAs on the same set of patients would contribute 5 study arms. Twenty percent (33/169) of the studies were from the United States, Fifteen percent (26/169) of the studies were from China and the remainder of the studies were from Italy (12/169), Germany (9/169), Belgium (8/169), France (7/169), Japan (6/169), UK (6/169), Australia (5/169), Spain (5/169), Switzerland (5/169), Brazil (4/169), Saudi Arabia (4/169), Singapore (4/169), Austria (3/169), Sweden (3/169), Canada (2/169), Ecuador (2/169), Liechtenstein (2/169), Netherlands (2/169), Thailand (2/169), Bangladesh (1/169), Chile (1/169), Colombia (1/169), Croatia (1/169), Finland (1/169), Greece (1/169), India (1/169), Iran (1/169), Israel (1/169), Kenya (1/169), Korea (1/169), Mexico (1/169), New Zealand (1/169), Nigeria (1/169), Qatar (1/169), Serbia (1/169), South Africa (1/169), Uganda (1/169), and United Arab Emirates (1/169). Three SARS-CoV-2 antigens, including surface protein (S), nucleocapsid protein (N) and envelope protein (E), were used either together or separately in the studies that were included in the review. The spike protein was used as the antigen in 31 study arms, and the nucleocapsid protein was used in 21 study arms. Fifty-two study arms separately used both S and N as antigens. In 19 study arms, S and N antigens (S-N) were used together as the antigen. In 17 study arms, N and E antigens (N-E) were used together as the antigen. The sample was collected from inpatients in 48 articles and in 11 articles regarding outpatients. Fifty-nine study arms were separately comprised of outpatients and inpatients. In 42 study arms, samples were collected from inpatients and outpatients together. Most of the serological assay test kits were commercial (*n* = 173 study arms), and 22 study arms involved in-house assays. When regarding the type of specimen used for the RT–PCR reference test, 70 study arms involved nasopharyngeal samples, and 47 study arms involved sputum, saliva or oral, throat and pharyngeal samples. Table 1 reports the characteristics of each individual study.

**Table 1:** Summary of characteristics of included studies, stratified by method of serological testing.

**ELISA**				**CLIA**			**LFIA**		
**Characteristics**	**No. of studies**	**No. of participants**	**COVID-19/healthy**	**No. of studies**	**No. of participants**	**COVID-19/healthy**	**No. of studies**	**No. of participants**	**COVID-19/healthy**
**Total**	**94**	**32,584**	**8,265/24,319**	**63**	**24,326**	**6,308/18,018**	**50**	**15,063**	**5,266/9,797**
Australia	4	2,472	352/2,120	1	209	71/138	2	476	143/333
Austria	2	240	81/159	1	571	230/341			
Bangladesh	1	184	79/105						
Belgium	5	1,010	550/460	4	691	362/329	3	608	333/275
Brazil	2	633	423/210	1	228	134/94	2	524	371/153
Canada	1	160	49/111	2	340	122/218			
China	8	2,060	1,105/955	16	6,052	2,851/3,201	9	1,609	806/803
Colombia				1	142	83/59			
Croatia	1	160	60/100	1	160	60/100	1	160	60/100
Denmark	1	736	150/586						
Ecuador	1	127	78/49						
Finland				1	151	70/81			
France	2	395	154/241	4	398	157/241	2	1,084	690/394
Germany	6	13,408	1,266/12,142	3	422	229/193	1	50	25/25
Greece	1	200	50/150						
India							1	50	25/25
Iran	1	179	67/112						
Israel	1	633	309/324						
Italy	6	5,541	509/5,032	5	5,489	400/5,089	4	4,861	366/4,495
Japan	1	317	143/174	3	1,265	397/868	3	549	235/314
Liechtenstein				1	1,338	145/1,193			
Kenya	1	665	149/516						
Korea							1	149	70/79
Mexico	1	378	149/229						
Netherlands	1	228	99/129				2	544	196/348
New Zealand	1	134	21/113						
Nigerian	1	195	96/99						
Qatar	1	171	101/70						
Saudi Arabia	4	881	291/590						
Serbia	1	118	50/68						
Spain	2	436	200/236				5	710	460/250
Singapore				4	2,021	660/1,361			
South Africa	1	512	373/139						
Sweden	2	485	239/246	2	399	199/200	1	302	152/150
Switzerland	4	2,353	451/1,902	2	1,450	327/1,123	1	91	41/50
Thailand	2	615	436/179						
Uganda	1	150	50/100						
United Arab Emirates	1	93	63/30	1	93	63/30			
UK	2	233	133/100	2	530	286/244	3	1,695	490/1,205
USA	24	14,419	3,224/11,195	8	5,647	1,041/4,606	9	3,993	2,121/1,872
**Time post-onset**									
First week	12	4,310	1,428/2,882	12	10,452	1,869/8,583	11	2,185	1,348/837
Second week	11	11,594	1,203/10,391	11	3,079	1,139/1,940	7	4,106	829/3,277
Third week	22	12867	3310/9607	16	8807	2604/6203	13	3055	1,693/1,362
Third week later (22–28 day)	9	5,274	1,788/3,486	10	5,805	1,771/2,034	7	2,671	1,015/1,656
**Antigen target**									
Surface protein	28	10,510	3,427/7,083	10	2,927	1,579/1,348	1	105	30/75
Nucleocapsid protein	22	16,359	1,748/14,611	7	3,332	800/2,532	4	873	415/458
Surface and nucleocapsid proteins	18	5,385	1,912/3,473	9	6,101	1,655/4,446	5	1,096	511/585
**Clinical setting**									
Inpatient only	21	5,896	1,822/4,074	18	6,354	2,536/3,818	10	1,822	889/933
Outpatient	7	6,408	692/5,716	2	2,709	170/2,539	4	5,103	456/4,647
Inpatient and outpatient	18	10,636	3,072/7,564	19	11,502	3,005/8,497	11	3,261	1,518/1,743
No reported	46	27,033	5,731/21,352	25	9,090	2,898/6,192	32	11,125	4,415/6,710
**Serological kit as index test**									
Commercial serological kit	81	45,393	9,592/35,851	55	23,704	7,024/16,680	50	20,426	6,905/13,521
In-house assay	16	5,397	2,233/3,164	5	4,134	990/3,144	2	2,275	1,289/986
Unclear	1	736	150/586	2	302	141/161	6	1,059	402/657
**Type of specimen for RT–PCR reference test**									
Nasopharyngeal	36	15,480	3,878/11,602	19	10,564	1,800/8,764	19	7,122	3,694/3,428
Sputum, saliva, or oral, throat, or pharyngeal	21	9,893	2,343/7,550	13	6,257	1,728/4,529	15	8,579	2,389/6,190
Not reported	44	31,019	6,379/24,690	36	14,664	5,010/9,654	29	8,865	2,872/5,993

TP, true positive; FN, false negative; TN, true negative; FP, false positive; ELISA, enzyme linked immunosorbent assay; LFIA, lateral flow immunoassay; CLIA, chemiluminescent immunoassay.

### Methodological qualities of the included studies

**Figure 2:**
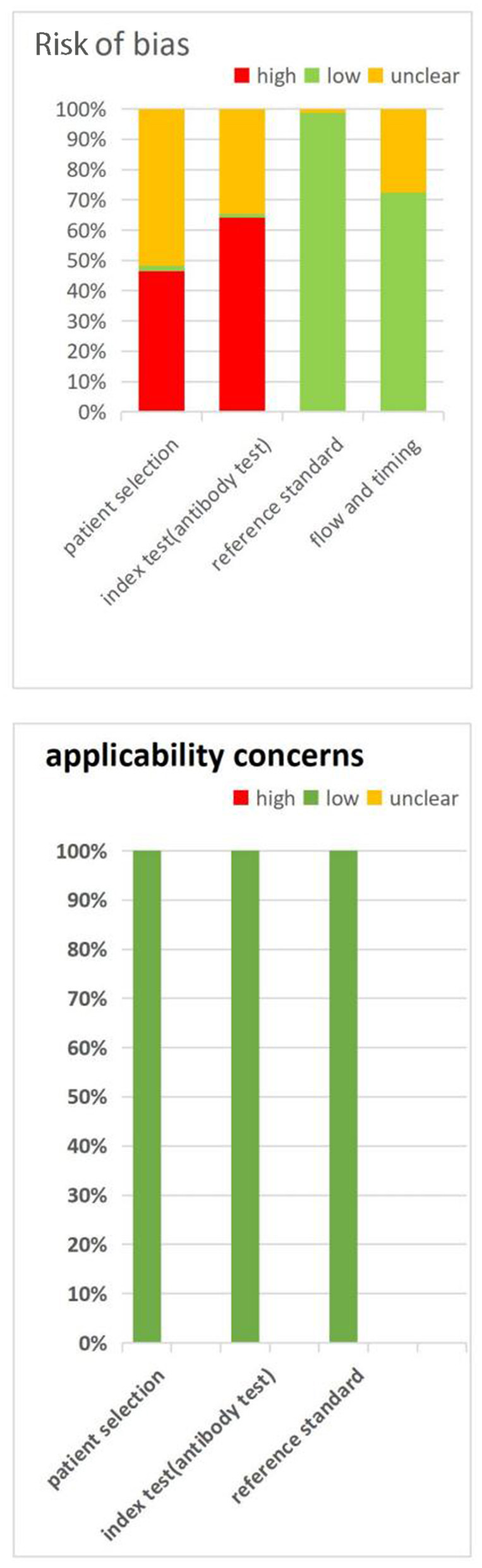
Quality assessment of QuaDas-2 assessment.

Figure 2 summarizes the QUADA-2 assessment, and Supplementary Table S1 provides details for each study QUADAS-2 evaluations. For the patient selection domain, a high or unclear risk of bias was observed in 98% (166/169) of the QUADAS-2 assessments, with the risks of bias mostly related to a case-control design and not due to conductive or random sampling. For the index test domain, 99% (167/169) of the assessments demonstrated a high or unclear risk of bias because it was not clear whether the serological test was interpreted blindly to the reference standard or whether the cut-off values for classifying the results were positive or negative. For the reference standard domain, 99% (167/169) of the assessments concluded a low risk of bias because the RT–PCR test is currently the best diagnostic method for use in novel coronavirus patients and is evaluated without knowing the results of the novel coronavirus serum test. The risk of bias from flow and timing was high or unclear in 27.2% (46/169) of the assessments, which was due to an appropriate time interval between the new coronavirus serum test that we investigated and the gold standard RT–PCR test. All of the patients underwent the same gold standard test, and most of the researched cases were included in the analysis.

### Overall sensitivity

Table 2 reports on the sensitivity that was stratified by test type and immunoglobulin class. Within each test method (CLIA, ELISA, and LFIA), point estimates were similar between the different types of immunoglobulins, and the confidence intervals overlapped. Within each class of immunoglobulin, the sensitivity was lowest for the LFIA method. The pooled sensitivity of the ELISAs measuring IgM was 71% (95% CI: 70–73%), with IgG being 84% (95% CI: 83–84%) and IgM or IgG being 84% (95% CI: 83–85%). The pooled sensitivity of the LFIAs measuring IgM was 65% (95% CI: 64–67%), with IgG being 73% (95% CI: 71–74%) and IgM/IgG being 69% (95% CI: 68–71%). The pooled sensitivity of the CLIAs measuring IgM was 70% (95% CI: 69–72%), with IgG being 80% (95% CI: 79–81%) and IgM/IgG being 87% (95% CI: 86–88%). For all of the test methods and immunoglobulin classes, visual inspections of the summary ROC curves (Supplementary Figure S1) and of the forest plots (Supplementary Figure S2) exhibited significant heterogeneity.

**Table 2:** Individual and pooled sensitivity by serological test method and immunoglobulin class detected.

		**IgM**			**IgG**			**IgM or IgG**	
**Method and studies**	**TP**	**FN**	**Sensitivity (%) (95% CI)**	**TP**	**FN**	**Sensitivity (%) (95% CI)**	**TP**	**FN**	**Sensitivity (%) (95% CI)**
**ELISA (*****n*** **=** **94 arms)**									
A Cramer (2021)				378	62	85.9 (82.3–89.0)			
Abdullah Algaissi (2020)	26	78	25.0 (17.0–34.4)	60	44	57.7 (47.6–67.3)			
Alexander Krüttgen (2020)				65	10	86.7 (76.8–93.4)			
Angel Guevara (2021)				73	5	93.6 (85.7–97.9)			
Anita S. Iyer (2020)	210	49	81.1 (75.8–85.7)	251	8	96.9 (94.0–98.7)			
Antoine-Reid. T (2020)				18	3	85.7 (63.7–97.0)			
Archana Thomas (2021)				36	14	72.0 (57.5–83.8)			
Ariel D. Stock (2020)				4	4	50.0 (15.7–84.3)			
Ayesha Appa (2020)	39	46	45.9 (35.0–57.0)	76	94	44.7 (37.1–52.5)			
B. Meyer (2020)				170	11	93.9 (89.4–96.9)			
Bijon Kumar Sil (2021)				75	4	94.9 (87.5–98.6)			
Bin Lou (2020)	74	6	92.5 (84.4–97.2)	71	9	88.8 (79.7–94.7)			
Carleen Klumpp-Thomas (2020)							194	95	67.1 (61.4–72.5)
Caturegli. G (2020)				301	7	97.7 (95.4–99.1)			
Chang Zhou (2020)	150	0	100 (97.6–100.0)	149	1	99.3 (96.3–100)			
Christian Wechselberger (2020)				50	1	98.0 (89.6–100)			
Clarence W. Chan (2020)				74	4	94.9 (87.4–98.6)	146	23	86.4 (80.3–91.2)
D. S. Y. Ong (2020)							193	96	66.8 (61.0–72.2)
Daniel Brigger (2020)				281	55	83.6 (79.2–87.4)			
David M (2020)				79	5	94.0 (86.7–98.0)	81	2	97.6 (91.6–99.7)
E. Catry (2020)				16	2	88.9 (65.3–98.6)			
Ekasit Kowitdamrong (2020)				84	15	84.8 (76.2–91.3)			
Eshan U. Patel (2020)				127	19	87.0 (80.4–92.0)			
Fei Xiang (2020)	51	15	77.3 (65.3–86.7)	55	11	83.3 (72.1–91.4)			
Gang Xu (2020)				26	0	100 (86.8–100)			
Giuseppe Vetrugno (2021)				129	35	78.7 (71.6–84.7)			
Gláucia Cota (2020)							242	47	83.7 (79.0–87.8)
Hadi M. Yassine (2020)				382	123	75.6 (71.7–79.3)			
Isabel Montesinos (2020)				79	49	61.7 (52.7–70.2)			
Isabelle Piec (2021)				214	37	85.3 (80.3–89.4)			
Iyer. A S (2020)	278	65	81.0 (76.5–85.1)	326	17	95.0 (92.2–97.1)			
Jeffrey D. Whitman (2020)							192	64	75.0 (69.2–80.2)
Jialin Xiang (2020)	20	6	76.9 (56.4–91.0)	20	6	76.9 (56.4–91.0)			
Jira Chansaenroj (2021)				176	47	78.9 (73.0–84.1)			
Joanna Jung (2020)				104	0	100 (96.5–100)			
Julien Favresse (2020)				750	340	68.8 (66.0–71.5)			
Julien Marlet (2020)				183	49	78.9 (73.1–83.9)			
Justin Manalac (2020)				97	0	100 (96.3–100)			
Katherine Bond (2020)				85	6	93.4 (86.2–97.5)			
Kristin E. Mullins (2021)							277	5	98.2 (95.9–99.4)
Lene H. Harritsh (2021)	187	113	62.3 (56.6–67.8)	281	9	96.9 (94.2–98.6)	289	11	96.3 (93.5–98.2)
Luciano F. Huergo (2021)							516	50	91.2 (88.5–93.4)
Marc Kovac (2020)							147	142	50.9 (44.9–56.8)
Margherita Bruni (2020)				54	2	96.4 (87.7–99.6)			
Maria Martínez Serrano (2020)				106	24	81.5 (73.8–87.8)			
Marie Tré-Hardy (2020)	12	27	30.8 (17.0–47.6)	42	2	95.5 (84.5–99.4)			
Marzia Nuccetelli (2020)				88	4	95.7 (89.2–98.8)			
Marzia Nuccetelli (2021)							84	3	96.6 (92.6–98.7)
Massimo Pieri (2020)	30	10	75.0 (58.8–87.3)	75	5	93.8 (86.0–97.9)			
Maximilian Kittel (2020)				137	46	74.9 (67.9–81.0)			
Melkon G. DomBourian (2020)				92	10	90.2 (82.7–95.2)			
N. Davidson (2020)	16	131	10.9 (6.4–17.1)	86	56	60.6 (52.0–68.7)			
Qiang Wang (2020)	14	0	100 (76.8–100)						
Reuben McGregor (2020)	4	17	19.0 (5.4–41.9)	21	0	100 (83.9–100)	21	0	100 (83.9–100)
Sarah E. Turbett (2020)				90	38	70.3 (61.6–78.1)			
Sarah M. Hicks (2020)							43	0	100 (91.8–100)
Stefani N. Thomas (2021)							68	11	86.1 (76.5–92.8)
Suliman A. Alharbi (2020)	35	5	87.5 (73.2–95.8)	37	3	92.5 (79.6–98.4)			
Tania ReginaTozetto-Mendoza (2021)				121	13	90.3 (84.0–94.7)			
Teodora Djukic (2021)	47	3	94.0 (83.5–98.7)	47	3	94.0 (83.5–98.7)			
Teresa Stock da Cunha (2020)	40	8	83.3 (69.8–92.5)	48	0	100 (92.6–100)			
Thamir A. Alandijany (2020)				109	0	100 (96.7–100)			
Thomas Nicol (2020)				141	0	100 (97.4–100)			
Thomas W. McDade (2020)				27	3	90.0 (73.5–97.9)			
Traugott M (2020)	8	22	26.7 (12.3–45.9)	1	29	3.3 (1.0–17.2)	11	19	36.7 (19.9–56.1)
Victoria Indenbaum (2020)	133	148	47.3 (41.4–53.3)	271	36	88.3 (84.1–91.7)			
Wanbing Liu (2020)	146	68	68.2 (61.5–74.4)	150	64	70.1 (63.5–76.1)	172	42	80.4 (74.4–85.5)
Zahra Rikhtegaran Tehrani (2020)	234	57	80.4 (75.4–84.8)	179	21	89.5 (84.4–93.4)			
Brad Poore (2021)							173	19	90.0 (85.0–94.0)
Valentina Pecoraro (2021)	20	4	83.0 (63.0–95.0)	22	2	92.0 (73.0–99.0)			
James Nyagwange (2021)				138	11	93.0 (87.0–96.0)	142	7	95.0 (91.0–98.0)
Pan-pan Liu (2021)	160	8	95.0 (91.0–98.0)	163	5	97.0 (93.0–99.0)	168	0	100.0 (98.0–100.0)
Maryam Ranjbar (2021)	62	5	93.0 (83.0–98.0)	61	6	91.0 (82.0–97.0)			
Marina Bubonja-Šonje (2021)	58	2	97.0 (88.0–100.0)	44	16	73.0 (60.0–84.0)			
Tom Lutalo (2021)	33	17	66.0 (51.0–79.0)	49	1	98.0 (89.0–100.0)	46	4	92.0 (81.0–98.0)
Oskar Ekelund (2021)				150	2	99.0 (95.0–100.0)			
P. J. Ducrest (2021)							21	2	91.0 (72.0–99.0)
Norihito Kaku (2021)							112	31	78.0 (71.0–85.0)
David Triest (2021)							108	0	100.0 (97.0–100.0)
Maemu P. Gededzha (2021)				239	134	64.0 (59.0–69.0)			
Robert Needle (2021)				48	0	100.0 (93.0–100.0)			
Arwa A. Faizo (2021)				90	0	100.0 (96.0–100.0)			
Rosa Camacho-Sandoval (2021)				148	1	99.0 (96.0–100.0)			
Theano Lagousi (2021)	35	15	70.0 (55.0–82.0)	46	4	92.0 (81.0–98.0)			
Adnan Alatoom (2021)							27	5	84.0 (67.0–95.0)
Fehintola Ige (2021)				68	28	71.0 (61.0–80.0)			
Ingrid Sander (2022)				91	5	95.0 (88.0–98.0)			
Shiji Wu (2022)	329	55	86.0 (82.0–89.0)	368	16	96.0 (93.0–98.0)			
Vijayalakshmi Nandakumar (2021)				116	8	94.0 (88.0–97.0)			
Elena Riester (2021)							116	8	94.0 (88.0–97.0)
Ismar A. Rivera-Olivero (2022)				106	21	83.0 (76.0–89.0)			
Nina Lagerqvist (2021)				71	16	82.0 (72.0–89.0)			
Ji Luo (2021)				61	7	90.0 (80.0–96.0)			
Suellen Nicholson (2021)				72	72	50.0 (42.0–58.0)			
Pooled	2,451	980	71.0 (70.0–73.0)	9,418	1,820	84.0 (83.0–84.0)	3,589	686	84.0 (83.0–85.0)
**LFIA (*****n*** **=** **55 arms)**									
A Cramer (2021)							62	16	79.5 (68.8–87.8)
Chao Huang (2020)	5	0	100 (47.8–100)						
Choe JY (2020)							65	5	92.9 (84.1–97.6)
Clarence W (2021)							90	9	90.9 (83.4–95.8)
D. S. Y. Ong (2020)							43	56	43.3 (33.5–53.8)
Diego O. Andrey (2020)							40	6	87.0 (73.7–95.1)
E Tuaillon (2020)	12	3	80.0 (51.9–95.7)	13	2	86.7 (59.5–98.3)			
E. Catry (2020)	43	1	97.7 (88.0–99.9)	39	13	75.0 (61.1–86.0)	25	1	96.2 (80.4–99.9)
Feng M (2020)	27	1	96.4 (81.7–99.9)	27	1	96.4 (81.7–99.9)			
Francis Stieber (2020)							30	0	100 (88.4–100)
Francisco Javier Candel González (2020)							35	0	100 (90.0–100)
Giovanni Sotgiu (2020)	6	1	85.7 (42.1–99.6)	4	3	57.1 (18.4–90.1)			
Giuseppe Vetrugno (2021)							104	60	63.4 (55.5–70.8)
Gladys VirginiaGuedez-López (2020)	28	22	56.0 (41.3–70.0)	26	24	52.0 (37.4–66.3)	268	167	61.6 (56.9–66.2)
Gláucia Cota (2020)							1,260	484	72.2 (70.1–74.3)
Hua Li (2020)	68	7	90.7 (81.7–96.2)	51	23	68.9 (57.1–79.2)	69	6	92.0 (83.4–97.0)
Isabel Montesinos (2020)	238	146	62.0 (56.9–66.9)				271	113	70.6 (65.7–75.1)
J. Van Elslande (2020)	60	93	39.2 (31.4–47.4)	95	58	62.1 (53.9–69.8)	100	53	65.4 (57.3–72.9)
Jeffrey D. Whitman (2020)	691	427	61.8 (58.9–64.7)	658	461	58.8 (55.9–61.7)	62	411	13.1 (10.2–16.5)
Kathrine McAulay (2020)							312	23	93.1 (89.9–95.6)
Klaus Puschel (2021)							176	128	57.9 (52.1–63.5)
Laurent Dortet (2020)							525	243	68.4 (64.9–71.6)
Linda Hueston (2020)	78	48	61.9 (52.8–70.4)	113	13	89.7 (83.0–94.4)	78	48	61.9 (52.8–70.4)
Lixia Zhang (2020)	120	7	94.5 (89.0–97.8)	121	6	95.3 (90.0–98.2)			
Maria Martínez Serrano (2020)	41	85	32.5 (24.5–41.5)	89	37	70.6 (61.9–78.4)			
Marta Cancella de Abreu (2020)							103	34	75.2 (67.1–82.2)
Maya Moshe (2021)				164	18	90.1 (84.8–94.0)	161	30	84.3 (78.3–89.1)
Morihito Takita (2020)				5	0	100 (47.8–100)			
Niko Kohmer (2020)				13	4	76.5 (50.1–93.2)			
Peter Findeisen (2020)							42	0	100 (91.6–100)
Qiang Wang (2020)	14	0	100 (76.8–100)						
Roselle S. Robosa (2020)	27	27	50.0 (36.1–63.9)	30	25	54.5 (40.6–68.0)	35	25	58.3 (44.9–70.9)
Scott J C Pallett (2020)							375	37	91.0 (87.8–93.6)
Shun Kaneko (2020)	69	18	79.3 (69.3–87.3)	75	6	92.6 (84.6–97.2)			
SilviaMontolio Breva (2021)							46	17	73.0 (60.3–83.4)
Thomas Nicol (2020)	141	0	100 (97.4–100)	141	0	100 (97.4–100)			
Tian Wen (2020)				38	17	69.1 (55.2–80.9)			
Vani Maya (2021)							23	2	91.0 (74.0–99.0)
Won Lee (2020)	44	6	88.0 (75.7–95.5)	42	8	84.0 (70.9–92.8)			
Yaqing Li (2020)							72	17	80.9 (71.2–88.5)
Yunbao Pan (2020)	48	38	55.8 (44.7–66.5)	47	39	54.7 (43.5–100)	59	27	68.6 (57.7–78.2)
Zahra Rikhtegaran Tehrani (2020)	82	18	82.0 (73.1–89.0)	92	8	92.0 (84.8–96.5)			
Ziad Daoud (2020)	156	64	70.9 (64.4–76.8)	159	61	72.3 (65.9–78.1)			
Jialin Xiang (2020/10)	13	37	26.0 (14.6–40.3)						
Valentina Pecoraro (2021)	16	8	67.0 (45.0–84.0)	22	2	92.0 (73.0–99.0)			
Marina Bubonja-Šonje (2021)	60	0	100.0 (94.0–100.0)	46	14	77.0 (64.0–87.0)			
Bianca A. Trombetta (2021)	51	5	91.0 (80.0–97.0)	52	4	93.0 (83.0–98.0)	51	5	91.0 (80.0–97.0)
Oskar Ekelund (2021)							110	42	72.0 (65.0–79.0)
Norihito Kaku (2021)	33	110	23.0 (16.0–31.0)	69	74	48.0 (40.0–57.0)			
Sérgio M. de Almeida (2021)	68	14	83.0 (73.0–90.0)	59	23	72.0 (61.0–81.0)	69	13	84.0 (74.0–91.0)
Amedeo De Nicolò (2021)							80	63	56.0 (47.0–64.0)
Dennis Souverein (2021)	17	80	18.0 (11.0–27.0)	77	20	79.0 (70.0–87.0)	78	19	80.0 (71.0–88.0)
Sophie I. Owen (2021)	67	33	67.0 (57.0–76.0)	51	49	51.0 (41.0–61.0)	70	30	70.0 (60.0–79.0)
Shiji Wu (2022)	268	116	70.0 (65.0–74.0)	353	31	92.0 (89.0–94.0)			
Ismar A. Rivera-Olivero (2022)	101	26	80.0 (71.0–86.0)	101	26	80.0 (71.0–86.0)			
Pooled	2,692	1,441	65.0 (64.0–67.0)	2,872	1,070	73.0 (71.0–74.0)	4,989	2,190	69.0 (68.0–71.0)
**CLIA (*****n*** **=** **64 arms)**									
Bin Lou (2020)	69	11	86.3 (76.7–92.9)	69	11	86.3 (76.7–92.9)			
Dachuan Lin (2020)	48	31	60.8 (49.1–71.6)	65	14	82.3 (72.1–90.0)			
Wanbing Liu (2020)	149	57	72.3 (65.7–78.3)				40	4	90.9 (78.3–97.6)
Maria Infantino (2020)	45	16	73.8 (60.9–84.2)	47	14	77 (64.5–86.8)			
Shao Lijia (2020)	9	6	60 (32.3–83.7)	22	3	88 (68.8–97.5)			
Fang Hu (2020)	51	17	75 (63.0–84.7)	57	11	83.8 (72.9–91.6)	104	12	89.7 (82.6–94.5)
Charpentier, C (2020)				4	2	66.7 (22.3–95.7)			
Jääskeläinen, A J (2020)				56	14	80 (68.7–88.6)			
Ping li (2020)	88	28	75.9 (67.0–83.3)	104	12	89.7 (82.6–94.5)			
Chew, K L (2020)				15	162	8.5 (4.8–13.6)			
Fabrizio Bonelli (2020)				275	147	65.2 (60.4–69.7)			
Narjis Boukli (2020)				292	140	67.6 (63.0–72.0)			
Andrew Bryan (2020)				66	59	52.8 (43.7–61.8)			
MarinaJohnson (2020)				189	7	96.4 (92.8–98.6)			
Niko Kohmer (2020)				35	10	77.8 (62.9–88.8)			
Z. Huang (2020)							325	21	93.9 (90.9–96.2)
Ayesha Appa (2020)				12	0	100 (73.5–100)			
Chungen Qian (2020)	441	72	86 (82.7–88.9)	496	17	96.7 (94.7–98.1)	77	45	63.1 (53.9–71.7)
Marie Tré-Hardy (2020)				40	81	33.1 (24.8–42.2)			
Isabel Montesinos (2020)	74	52	58.7 (49.6–67.4)	67	59	53.2 (44.1–62.1)			
Julien Marlet (2020)				49	9	84.5 (72.6–92.7)			
Elitza S. Theel (2020)				5	71	6.6 (2.2–14.7)			
Massimo Pieri (2020)	30	10	75 (58.8–87.3)	36	4	90 (76.3–97.2)	363	46	88.8 (85.3–91.6)
Thomas Nicol (2020)				141	0	100 (97.4–100)			
Morihito Takita (2020)				5	0	100 (47.8–100)			
Raymond T (2020)	158	113	58.3 (52.2–64.2)	188	83	69.4 (63.5–74.8)	130	20	86.7 (80.2–91.7)
Justin Manalac (2020)				208	17	92.4 (88.2–95.5)	264	34	88.6 (84.4–92.0)
C. S. Lau (2020)				270	9	96.8 (94.0–98.5)			
Yafang Wan (2020)	109	41	72.7 (64.8–79.6)	130	20	86.7 (80.2–91.7)	46	4	92 (80.8–97.8)
E. Catry (2020)				16	2	88.9 (65.3–98.6)	17	1	94.4 (72.7–99.9)
Li-xiang Wu (2020)	126	26	82.9 (76.0–88.5)	138	14	90.8 (85.0–94.9)	146	6	96.1 (91.6–98.5)
Jenna Rychert (2020)							36	3	92.3 (79.1–98.4)
Anja Dörschug (2020)							38	54	41.3 (31.1–52.1)
Nan wu (2020)	9	23	28.1 (13.7–46.7)	25	7	78.1 (60.0–90.7)			
Shey-Ying Chen (2020)							325	21	93.9 (90.9–96.2)
Xueping Qiu (2020)	356	53	87 (83.4–90.1)	356	53	87 (83.4–90.1)	364	46	88.8 (85.3–91.7)
Hélène Haguet (2021)	28	110	20.3 (13.9–28.0)	131	7	94.9 (89.8–97.9)			
Huihui Wang (2021)							39	1	97.5 (86.8–99.9)
Rasmus Strand MSc (2021)	7	40	14.9 (6.2–28.3)	41	6	87.2 (74.3–95.2)			
Tania ReginaTozetto-Mendoza (2021)				121	13	90.3 (84–94.7)			
C. S. Lau (2021)							129	4	97 (92.5–99.2)
Luigi Vimercati (2021)	5	13	27.8 (9.7–53.5)	10	8	55.6 (30.8–78.5)			
Sousuke Kubo1 (2021)				202	0	100 (98.2–100)	202	0	100 (98.2–100)
Anna Schaffner (2020)							234	117	66.7 (61.5–71.6)
N. DAVIDSON (2020)				45	26	63.4 (51.1–74.5)			
A Cramer (2021)							68	7	90.7 (81.7–96.2)
Victoria Higgins (2021)	52	21	71.2 (59.4–81.2)	85	61	58.2 (49.8–66.3)	45	28	61.6 (49.5–72.8)
Myriam C. Weber (2020)				265	25	91.4 (87.5–94.3)	139	6	95.9 (91.2–98.5)
Maximilian Kittel (2020)	112	254	30.6 (25.9–35.6)	324	225	59 (54.8–63.2)	141	97	59.2 (52.7–65.5)
Christian Irsara (2021)				359	62	85.3 (81.5–88.5)	390	35	91.8 (88.7–94.2)
Yaqing Li (2020)							347	10	97.2 (94.9–98.6)
Yuki Nakano (2021)	173	13	93 (88.3–96.2)	164	22	88.2 (82.6–92.4)			
Brad Poore (2021)				189	3	59.0 (54.0–65.0)			
Valentina Pecoraro (2021)	20	4	83.0 (63.0–95.0)	22	2	92.0 (73.0–99.0)			
Gabriel N Maine (2022)				239	11	96.0 (92.0–98.0)			
Kotaro Aoki (2021)				135	71	66.0 (59.0–72.0)			
Marina Bubonja-Šonje (2021)				58	2	97.0 (88.0–100.0)			
Oskar Ekelund (2021)				123	29	81.0 (74.0–87.0)			
Lau CS (2021)	68	65	51.0 (42.0–60.0)						
Maria del Mar Castro (2021)				69	14	83.0 (73.0–90.0)			
Robert Needle (2021)				47	2	96.0 (86.0–100.0)			
Adnan Alatoom (2021)				27	5	84.0 (67.0–95.0)			
Shiji Wu (2022)	355	29	92.0 (89.0–95.0)	354	30	92.0 (89.0–95.0)			
Vijayalakshmi Nandakumar (2021)				117	7	94.0 (89.0–98.0)			
Pooled	2,582	1,399	70.0 (69.0–72.0)	6,609	1,683	80.0 (79.0–81.0)	4,009	622	87.0 (86.0–88.0)

Ig, immunoglobulin; TP, true positive; FN, false negative; TN, true negative; FP, false positive; ELISA, enzyme linked immunosorbent assay; LFIA, lateral flow immunoassay; CLIA, chemiluminescent immunoassay.

### Overall specificity

Table 3 describes the within study and pooled specificities, as stratified by test type and immunoglobulin class. The pooled specificity of the ELISAs measuring IgM was 98% (95% CI: 98–99%), with IgG being 96% (95% CI: 95–96%), and IgM or IgG being 99% (95% CI: 99–99%). The pooled specificity of the LFIAs measuring IgM was 96% (95% CI: 96–97%), with IgG being 97.0% (95% CI: 96–97%) and IgM or IgG being 97% (95% CI: 97–98%). The pooled specificity of the CLIAs measuring IgM was 94% (95% CI: 93–95%), with IgG being 98% (95% CI: 98–99%) and IgM or IgG being 97% (95% CI: 97–97%). Within each class of immunoglobulin, the specificity was the lowest for the IgM-based CLIA tests. All of the tests displayed high specificity (ranging from 93.0 to 99.0%). For all of the test methods and immunoglobulin classes, visual inspections of the summary ROC curves (Supplementary Figure S1) and of the forest plots (Supplementary Figure S3) showed meta-analytical estimates of specificity (with a value of 95%) by using the serological test method and antibody class.

**Table 3:** Individual and pooled specificity by serological test method and immunoglobulin class detected.

	**IgM**				**IgG**			**IgM or IgG**	
**Method and studies**	**TN**	**FP**	**Specificity (95% CI)**	**TN**	**FP**	**Specificity (95% CI)**	**TN**	**FP**	**Specificity (95% CI)**
**ELISA (*****n*** **=** **94 arms)**									
A Cramer (2021)				241	9	96.4 (93.3–98.3)			
Abdullah Algaissi (2020)	240	10	96.0 (92.8–98.1)	236	14	94.4 (90.8–96.9)			
Alexander Krüttgen (2020)				24	1	96.0 (79.6–99.9)			
Angel Guevara (2021)				49	0	100 (92.7–100)			
Anita S. Iyer (2020)	1,548	0	100 (99.8–100)	1,548	0	100 (99.8–100)			
Antoine-Reid. T (2020)				187	53	77.9 (72.1–83.0)			
Archana Thomas (2021)				300	0	100 (98.8–100)			
Ariel D. Stock (2020)				79	11	87.8 (79.2–93.7)			
Ayesha Appa (2020)	246	15	94.3 (90.7–96.7)	492	30	94.3 (91.9–96.1)			
B. Meyer (2020)				316	10	96.9 (94.4–98.5)			
Bijon Kumar Sil (2021)				102	3	97.1 (91.9–99.4)			
Bin Lou (2020)	300	0	100 (98.8–100)	300	0	100 (98.8–100)			
Carleen Klumpp-Thomas (2020)							111	5	95.7 (90.2–98.6)
Caturegli. G (2020)				561	7	98.8 (97.5–99.5)			
Chang Zhou (2020)	144	6	96.0 (91.5–98.5)	150	150	50.0 (44.2–55.8)			
Christian Wechselberger (2020)				46	13	78.0 (65.3–87.7)			
Clarence W Chan (2020)				53	0	100 (93.3–100)	57	0	100 (93.7–100)
D. S. Y. Ong (2020)							114	2	98.3 (93.9–99.8)
Daniel Brigger (2020)				1,650	105	94.0 (92.8–95.1)			
David M (2020)				172	16	91.5 (86.5–95.1)	188	0	100 (98.1–100)
E. Catry (2020)				100	0	100 (96.4–100)			
Ekasit Kowitdamrong (2020)				166	5	97.1 (93.3–99.0)			
Eshan U. Patel (2020)				548	14	97.5 (95.9–98.6)			
Fei Xiang (2020)	60	0	100 (94.0–100)	57	3	95.0 (86.1–99.0)			
Gang Xu (2020)				120	2	98.4 (94.2–99.8)			
Giuseppe Vetrugno (2021)				4,290	28	99.4 (99.1–99.6)			
Gláucia Cota (2020)							62	54	53.4 (44.0–62.8)
Hadi M. Yassine (2020)				318	32	90.9 (87.3–93.7)			
Isabel Montesinos (2020)				71	1	98.6 (92.5–100)			
Isabelle Piec (2021)				588	20	96.7 (95.0–98.0)			
Iyer. A S (2020)	1,546	2	99.9 (99.5–100)	1,546	2	99.9 (99.5–100)			
Jeffrey D. Whitman (2020)							107	1	99.1 (94.9–100)
Jialin Xiang (2020)	37	4	90.2 (76.9–97.3)	39	2	95.1 (83.5–99.4)			
Jira Chansaenroj (2021)				130	0	100 (97.2–100)			
Joanna Jung (2020)				36	2	94.7 (82.3–99.4)			
Julien Favresse (2020)				470	2	99.6 (98.5–99.9)			
Julien Marlet (2020)				662	50	93.0 (90.8–94.7)			
Justin Manalac (2020)				35	72	32.7 (24.0–42.5)			
Katherine Bond (2020)				953	356	72.8 (70.3–75.2)			
Kristin E. Mullins (2021)							440	1	99.8 (98.7–100)
Lene H. Harritsh (2021)	979	6	99.4 (98.7–99.8)	1,182	14	98.8 (98.0–99.4)	1,273	6	99.5 (99.0–99.8)
Luciano F. Huergo (2021)							1,019	9	99.1 (98.3–99.6)
Marc Kovac (2020)							82	34	70.7 (61.5–78.8)
Margherita Bruni (2020)				414	22	95.0 (92.5–96.8)			
Maria Martínez Serrano (2020)				84	0	100 (95.7–100)			
Marie Tré-Hardy (2020)	79	0	100 (95.4–100)	79	2	97.5 (91.4–99.7)			
Marzia Nuccetelli (2020)				82	6	93.2 (85.7–97.5)			
Marzia Nuccetelli (2021)							118	0	100 (96.9–100)
Massimo Pieri (2020)	40	0	100 (91.2–100)	80	0	100 (95.5–100)			
Maximilian Kittel (2020)				91	6	93.8 (87.0–97.7)			
Melkon G. DomBourian (2020)				106	0	100 (96.6–100)			
N. Davidson (2020)	55	7		259	17	93.8 (90.3–96.4)			
Qiang Wang (2020)	50	22	69.4 (57.5–79.8)						
Reuben McGregor (2020)	195	0	100 (98.1–100)	195	0	100 (98.1–100)	113	0	100 (96.8–100)
Sarah E. Turbett (2020)				1,259	9	99.3 (98.7–99.7)			
Sarah M Hicks (2020)							182	2	98.9 (96.1–99.9)
Stefani N. Thomas (2021)							237	0	100 (98.5–100)
Suliman A Alharbi (2020)	63	2	96.9 (89.3–99.6)	1	4	20.0 (0.5–71.6)			
Tania ReginaTozetto-Mendoza (2021)				92	2	97.9 (92.5–99.7)			
Teodora Djukic (2021)	66	2	97.1 (89.8–99.6)	67	1	98.5 (92.1–100)			
Teresa Stock da Cunha (2020)	150	2	98.7 (95.3–99.8)	149	3	98.0 (94.3–99.6)			
Thamir A. Alandijany (2020)				304	5	98.4 (96.3–99.5)			
Thomas Nicol (2020)				147	5	96.7 (92.5–98.9)			
Thomas W. McDade (2020)				13	2	86.7 (59.5–98.3)			
Traugott M (2020)	97	3	97.0 (91.5–99.4)	98	2	98.0 (93.0–99.8)	97	3	97.0 (91.5–99.4)
Victoria Indenbaum (2020)	180	0	100 (98.0–100)	318	6	98.1 (96.0–99.3)			
Wanbing Liu (2020)	100	0	100 (96.4–100)	100	0	100 (96.4–100)	100	0	100 (96.4–100)
Zahra Rikhtegaran Tehrani (2020)	870	15	98.3 (97.2–99.0)	591	9	98.5 (97.2–99.3)			
Brad Poore (2021)							129	0	100.0 (97.0–100.0)
Valentina Pecoraro (2021)	2	13	13.0 (2.0–40.0)	2	13	13.0 (2.0–40.0)			
James Nyagwange (2021)				515	1	100.0 (99.0–100.0)	507	9	98.0 (97.0–99.0)
Pan-pan Liu (2021)	90	0	100 (96.0–100)	90	0	100.0 (96.0–100.0)	90	0	100.0 (96.0–100.0)
Maryam Ranjbar (2021)	109	3	97.0 (92.0–99.0)	110	2	98.0 (94.0–100.0)			
Marina Bubonja-Šonje (2021)	100	0	100 (96.0–100)	99	1	99.0 (95.0–100.0)			
Tom Lutalo (2021)	90	10	90.0 (82.0–95.0)	98	2	98.0 (93.0–100.0)	89	11	89.0 (81.0–94.0)
Oskar Ekelund (2021)				148	2	99.0 (95.0–100.0)			
P. J. Ducrest (2021)							95	3	97.0 (91.0–99.0)
Norihito Kaku (2021)							174	0	100.0 (98.0–100.0)
David Triest (2021)							84	5	94.0 (87.0–98.0)
Maemu P. Gededzha (2021)				132	7	95.0 (90.0–98.0)			
Robert Needle (2021)				109	2	98.0 (94.0–100.0)			
Arwa A. Faizo (2021)				91	1	99.0 (94.0–100.0)			
Rosa Camacho-Sandoval (2021)				224	5	98.0 (95.0–99.0)			
Theano Lagousi (2021)	137	13	91.0 (86.0–95.0)	146	4	97.0 (93.0–99.0)			
Adnan Alatoom (2021)							30	0	100.0 (88.0–100.0)
Fehintola Ige (2021)				99	0	100.0 (96.0–100.0)			
Ingrid Sander (2022)				176	7	96.0 (92.0–98.0)			
Shiji Wu (2022)	139	3	98.0 (94.0–100.0)	138	4	97.0 (93.0–99.0)			
Vijayalakshmi Nandakumar (2021)				184	8	96.0 (92.0–98.0)			
Elena Riester (2021)							9,561	14	100.0 (100.0–100.0)
Ismar A. Rivera-Olivero (2022)				40	0	100.0 (91.0–100.0)			
Nina Lagerqvist (2021)				95	1	99.0 (94.0 to100.0)			
Ji Luo (2022)				1,489	1	100.0 (100.0–100.0)			
Suellen Nicholson (2021)				179	30	86.0 (80.0–90.0)			
Pooled	7,712	138	98.0 (98.0–99.0)	26,510	1,219	96.0 (95.0–96.0)	15,059	159	99.0 (99.0–99.0)
**LFIA (*****n*** **=** **55 arms)**									
A Cramer (2021)							20	0	100 (93.2–100)
Chao Huang (2020)	13	1	92.9 (66.1–99.8)						
Choe JY (2020)							67	12	84.8 (75.0 to91.9)
Clarence W (2021)							165	3	98.2 (94.9–99.6)
D. S. Y. Ong (2020)							126	3	97.7 (93.4–99.5)
Diego O. Andrey (2020)							44	1	97.8 (88.2–99.9)
E Tuaillon (2020)	19	1	95.0 (75.1–99.9)	20	0	100 (83.2–100)			
E. Catry (2020)	288	12	96.0 (93.1–97.9)	396	4	99.0 (97.5–99.7)	200	0	100 (98.2–100)
Feng M (2020)	72	5	93.5 (85.5–97.9)	77	0	100 (95.3–100)			
Francis Stieber (2020)							75	0	100 (95.2–100)
Francisco Javier Candel González (2020)							5	0	100 (47.8–100)
Giovanni Sotgiu (2020)	1	21	45.0 (1.0–22.8)	16	6	72.7 (49.8–89.3)			
Giuseppe Vetrugno (2021)							4,173	145	96.6 (96.1–97.2)
Gladys VirginiaGuedez-López (2020)	76	19	80.0 (70.5–87.5)	80	15	84.2 (75.3–90.9)	34	26	56.7 (43.2–69.4)
Gláucia Cota (2020)							668	28	96.0 (94.2–97.3)
Hua Li (2020)	136	3	97.8 (93.8–99.6)	138	1	99.3 (96.1–100)	135	4	97.1 (92.8–99.2)
Isabel Montesinos (2020)	213	3	98.6 (96.0–99.7)				213	3	98.6 (96.0–99.7)
J. Van Elslande (2020)	94	9	91.3 (84.1–95.9)	101	2	98.1 (93.2–99.8)	93	10	90.3 (82.9–95.2)
Jeffrey D. Whitman (2020)	900	56	94.1 (92.5–95.5)	933	23	97.6 (96.4–98.5)	1,000	0	100 (99.6–100)
Kathrine McAulay (2020)							416	4	99.0 (97.6–99.7)
Klaus Puschel (2021)							215	3	98.6 (96–99.7)
Laurent Dortet (2020)							150	0	100 (97.6–100)
Linda Hueston (2020)	2,621	8	99.7 (99.4–99.9)	2,607	22	99.2 (98.7–99.5)	2,626	2	99.9 (99.7–100)
Lixia Zhang (2020)	20	0	100 (83.2–100)	20	0	100 (83.2–100)			
Maria Martínez Serrano (2020)	79	5	94.0 (86.7–98.0)	83	1	98.8 (93.5–100)			
Marta Cancella de Abreu (2020)							16	4	80.0 (6.3–94.3)
Maya Moshe (2021)				500	0	100 (99.3–100)	250	0	100 (98.5–100)
Morihito Takita (2020)				38	2	95.0 (83.1–99.4)			
Niko Kohmer (2020)				19	0	100 (82.4–100)			
Peter Findeisen (2020)							89	3	96.7 (90.8–99.3)
Qiang Wang (2020)	50	22	69.4 (57.5–79.8)						
ROSELLE S. ROBOSA (2020)	71	0	100 (94.9–100)	70	1	98.6 (92.4–100)	70	1	98.6 (92.4–100)
Scott J C Pallett (2020)							286	16	94.7 (91.5–96.9)
Shun Kaneko (2020)	100	0	100 (96.4–100)	100	0	100 (96.4–100)			
Silvia Montolio Breva (2021)							59	2	96.7 (88.7–99.6)
Thomas Nicol (2020)	145	7	95.4 (90.7–98.1)	147	5	96.7 (92.5–98.9)			
Tian Wen (2020)				30	0	100 (88.4–100)			
Vani Maya (2021)							25	0	100 (86.3–100)
Won Lee (2020)	47	3	94.0 (83.5–98.7)	49	1	98.0 (89.4–99.9)			
Yaqing Li (2020)							291	9	97.0 (94.4–98.6)
Yunbao Pan (2020)	8	14	36.4 (17.2–59.3)	13	9	59.1 (36.4–79.3)	14	8	63.6 (40.7–82.8)
Zahra Rikhtegaran Tehrani (2020)	275	25	91.7 (87.9–94.5)	280	20	93.3 (89.9–95.9)			100.0 (48.0–100.0)
Ziad Daoud (2020)	363	2	99.5 (98.0–99.9)	365	0	100 (99.0–100)			97.0 (94.0–99.0)
Jialin Xiang ((2020)/10)	13	4	76.5 (50.1–93.2)						
Valentina Pecoraro (2021)	3	12	20.0 (4.0–48.0)	3	12	20.0 (4.0–48.0)			
Marina Bubonja-Šonje (2021)	100	0	100.0 (96.0–100.0)	100	0	100.0 (96.0–100.0)			
Bianca A. Trombetta (2021)	55	1	98.0 (90.0–100.0)	56	0	100.0 (94.0–100.0)	56	0	100.0 (94.0–100.0)
Oskar Ekelund (2021)							145	5	97.0 (92.0–99.0)
Norihito Kaku (2021)	174	0	100.0 (98.0–100.0)	172	2	99.0 (96.0–100.0)			
Sérgio M. de Almeida (2021)	37	0	100.0 (91.0–100.0)	37	0	100.0 (91.0–100.0)	37	0	100.0 (91.0–100.0)
Amedeo De Nicolò (2021)							75	4	95.0 (88.0–99.0)
Dennis Souverein (2021)	218	1	100.0 (97.0–100.0)	218	1	100.0 (97.0–100.0)	218	1	100.0 (97.0–100.0)
Sophie I. Owen (2021)	86	19	82.0 (73.0–89.0)	97	98	50.0 (43.0–57.0)	85	20	81.0 (72.0–88.0)
Shiji Wu (2022)	142	0	100.0 (97.0–100.0)	142	0	100.0 (97.0–100.0)			
Ismar A. Rivera-Olivero (2022)	37	3	93.0 (80.0–98.0)	40	0	100.0 (91.0–100.0)			
Pooled	5,604	256	96.0 (96.0–97.0)	6,947	225	97.0 (96.0–97.0)	12,141	317	97.0 (97.0–98.0)
**CLIA (*****n*** **=** **64 arms)**									
Bin Lou (2020)	298	2	99.3 (97.6–99.9)	299	1	99.7 (98.2–100)			
Dachuan Lin (2020)	74	6	92.5 (84.4–97.2)	78	2	97.5 (91.3–99.7)			
Wanbing Liu (2020)	268	2	99.3 (97.4–99.9)				81	0	100 (95.5–100)
Maria Infantino (2020)	59	5	92.2 (82.7–97.4)	64	0	100 (94.4–100)			
Shao Lijia (2020)	50	0	100 (92.9–100)	50	0	100 (92.9–100)			
Fang Hu (2020)	109	1	99.1 (95.0–100)	106	4	96.4 (91.0–99.0)	134	0	100 (97.3–100)
Charpentier, C (2020)				50	2	96.2 (86.8–99.5)			
Jääskeläinen, A J (2020)				77	4	95.1 (87.8–98.6)			
Ping li (2020)	126	8	94 (88.6–97.4)	133	1	99.3 (95.9–100)			
Chew, K L (2020)				163	0	100 (97.8–100)			
Fabrizio Bonelli (2020)				4,923	117	97.7 (97.2–98.1)			
Narjis Boukli (2020)				297	3	99 (97.1–99.8)			
Andrew Bryan (2020)				1,010	10	99 (98.2–99.5)			
MarinaJohnson (2020)				189	5	97.4 (94.1–99.2)			
Niko Kohmer (2020)				35	0	100 (90.0–100)			
Z. Huang (2020)							190	4	97.9 (94.8–99.4)
Ayesha Appa (2020)				1,874	6	99.7 (99.3–99.9)			
Chungen Qian (2020)	946	26	97.3 (96.1–98.2)	947	25	97.4 (96.2–98.3)	72	0	100 (95.0–100)
Marie Tré-Hardy (2020)				4	0	100 (39.8–100)			
Isabel Montesinos (2020)	72	0	100 (95.0–100)	72	0	100 (95.0–100)			
Julien Marlet (2020)				176	2	98.9 (96.0–99.9)			
Elitza S. Theel (2020)				297	1	99.7 (98.1–100)			
Massimo Pieri (2020)	40	0	100 (91.2–100)	40	0	100 (91.2–100)	374	15	96.1 (93.7–97.8)
Thomas Nicol (2020)				151	1	99.3 (96.4–100)			
Morihito Takita (2020)				38	2	95 (83.1–99.4)			
Raymond T (2020)	234	1	99.6 (97.7–100)	233	2	99.1 (97.0–99.9)	355	35	91 (87.7–93.7)
Justin Manalac (2020)				1,236	25	98 (97.1–98.7)	55	6	90.2 (79.8–96.3)
C. S. Lau (2020)				978	2	99.8 (99.3–100)			
Yafang Wan (2020)	371	19	95.1 (92.5–97.0)	355	35	91 (87.7–93.7)	129	1	99.2 (95.8–100)
E. Catry (2020)				98	2	98 (93–99.8)	100	0	100 (96.4–100)
Li-xiang Wu (2020)	229	5	97.9 (95.1–99.3)	219	15	93.6 (89.6–96.4)	215	19	91.9 (87.6–95.0)
Jenna Rychert (2020)							99	1	99 (94.6–100)
Anja Dörschug (2020)							189	3	98.4 (95.5–99.7)
Nan wu (2020)	34	0	100 (89.7–100)	32	2	94.1 (80.3–99.3)			
Shey-Ying Chen (2020)							190	4	97.9 (94.8–99.4)
Xueping Qiu (2020)	377	12	96.9 (94.7–98.4)	377	12	96.9 (94.7–98.4)	374	15	96.1 (93.7–97.8)
Hélène Haguet (2021)	75	1	98.7 (92.9–100)	76	0	100 (95.3–100)			
Huihui Wang (2021)							94	94	50 (42.6–57.4)
Rasmus Strand MSc (2021)	45	5	90 (78.2–96.7)	49	1	98 (89.4–99.9)			
Tania ReginaTozetto-Mendoza (2021)				92	2	97.9 (92.5–99.7)			
C. S. Lau (2021)							245	3	98.8 (96.5–99.7)
Luigi Vimercati (2021)	2,117	272	88.6 (87.3–89.9)	2,341	48	98 (97.3–98.7)			
Sousuke Kubo (2021)				1,000	0	100 (99.6–100)	1,000	0	100 (99.6–100)
Anna Schaffner (2020)							1,157	2	99.8 (99.4–100)
N. Davidson (2020)				130	8	94.2 (88.9–97.5)			
A Cramer (2021)							25	0	100 (86.3–100)
Victoria Higgins (2021)	107	0	100 (96.6–100)	214	0	100 (98.3–100)	107	0	100 (96.6–100)
Myriam C. Weber (2020)				2,377	9	99.6 (99.3–99.8)	1,192	1	99.9 (99.5–100)
Maximilian Kittel (2020)	188	6	96.9 (93.4–98.9)	288	3	99 (97.0–99.8)	42	0	100 (91.6–100)
Christian Irsara (2021)				487	2	99.6 (98.5–100)	627	2	99.7 (98.9–100)
Yaqing Li (2020)							204	10	95.3 (91.6–97.7)
Yuki Nakano (2021)	125	19	86.8 (80.2–91.9)	144	0	100 (97.5–100)			
Brad Poore (2021)				129	0	100.0 (97.0–100.0)			
Valentina Pecoraro (2021)	2	13	13.0 (2.0–40.0)	2	13	13.0 (2.0–40.0)			
Gabriel N Maine (2022)				302	3	99.0 (97.0–100.0)			
Kotaro Aoki (2021)				677	7	99.0 (98.0–100.0)			
Marina Bubonja-Šonje (2021)				99	1	99.0 (95.0–100.0)			
Oskar Ekelund (2021)				149	1	99.0 (96.0–100.0)			
Lau CS (2021)	248	0	100.0 (99.0–100.0)						
Maria del Mar Castro (2021)				59	0	100.0 (94.0–100.0)			
Robert Needle (2021)				109	2	98.0 (94.0–100.0)			
Adnan Alatoom (2021)				30	0	100.0 (88.0–100.0)			
Shiji Wu (2022)	142	0	100.0 (97.0–100.0)	142	0	100.0 (97.0–100.0)			
Vijayalakshmi Nandakumar (2021)				189	3	98.0 (96.0–100.0)			
Pooled	6,336	403	94.0 (93.0–95.0)	23,686	384	98.0 (98.0–99.0)	7,250	215	97.0 (97.0–97.0)

Ig, immunoglobulin; TP, true positive; FN, false negative; TN, true negative; FP, false positive; ELISA, enzyme linked immunosorbent assay; LFIA, lateral flow immunoassay; CLIA, chemiluminescent immunoassay.

### Sensitivity and specificity by potential sources of heterogeneity

Table 4 reports the stratified meta-analyses for evaluating potential sources of heterogeneity in sensitivity and specificity. Heterogeneity was observed in all of the analyses.

**Table 4:** Accuracy of COVID-19 serology tests stratified by potential sources of heterogeneity.

**Subgroup**	**IgM**				**IgG**				**IgM or IgG**			
	**No. of arms (studies)**	**TP**	**FN**	**Pooled sensitivity (95% CI)**	**No. of arms**	**TP**	**FN**	**Pooled sensitivity (95% CI)**	**No. of arms**	**TP**	**FN**	**Pooled sensitivity (95% CI)**
**Time post-onset**												
**ELISA**												
First week	3	80	86	48.0 (40.0–56.0)	7	704	131	84.0 (82.0–87.0)	4	445	77	85.0 (82.0–88.0)
Second week	4	212	107	66.0 (61.0–72.0)	8	491	147	77.0 (73.0–80.0)	5	482	78	86.0 (83.0–89.0)
Third week	6	925	184	83.0 (81.0–86.0)	20	2,502	245	91.0 (90.0–92.0)	2	123	2	98.0 (94.0–100.0)
Third week later	1	62	5	93.0 (83.0–98.0)	6	844	171	83.0 (81.0–85.0)	2	194	21	90.0 (85.0–94.0)
**CLIA**												
First week	6	666	405	62.0 (59.0–65.0)	11	1,086	729	60.0 (58.0–62.0)	2	334	298	53.0 (49.0–57.0)
Second week	5	462	433	52.0 (48.0–55.0)	9	772	452	63.0 (60.0–66.0)	4	654	258	72.0 (69.0–75.0)
Third week	6	686	2,249	23.0 (22.0–25.0)	13	1,790	3,727	32.0 (31.0–34.0)	6	1,152	759	60.0 (58.0–62.0)
Third week later	5	495	238	68.0 (64.0–71.0)	6	1,159	255	82.0 (80.0–84.0)	2	467	58	89.0 (86.0–92.0)
**LFIA**												
First week	5	109	300	27.0 (22.0–31.0)	5	119	291	29.0 (25.0–34.0)	16	1,725	1,080	61.0 (60.0–63.0)
Second week	3	169	2,723	6.0 (5.0–7.0)	3	204	2,711	7.0 (6.0–8.0)	6	501	3,027	14.0 (13.0–15.0)
Third week	12	547	474	54.0 (50.0–57.0)	12	672	698	49.0 (46.0–52.0)	9	1,192	1,329	47.0 (45.0–49.0)
Third week later	3	68	85	44.0 (36.0–53.0)	3	129	24	84.0 (78.0–90.0)	7	646	624	51.0 (48.0–54.0)
Antigen target												
**ELISA**												
Surface protein	6	449	280	62.0 (58.0–65.0)	24	2,513	592	81.0 (80.0–82.0)	8	835	148	85.0 (83.0–87.0)
Nucleocapsid protein	7	537	96	85.0 (82.0–88.0)	21	1,513	292	84.0 (82.0–85.0)	2	284	8	97.0 (95.0–99.0)
Surface and nucleocapsid proteins	5	480	336	59.0 (55.0–62.0)	13	2,490	782	76.0 (75.0–78.0)	6	629	94	87.0 (84.0–89.0)
**CLIA**												
Surface protein	3	254	371	41.0 (37.0–45.0)	8	956	390	71.0 (69.0–73.0)	3	487	181	73.0 (69.0–76.0)
Nucleocapsid protein	0				5	464	144	76.0 (73.0–80.0)	3	421	29	94.0 (91.0–96.0)
Surface and nucleocapsid proteins	4	770	324	70.0 (68.0–73.0)	9	1,668	793	68.0 (66.0–70.0)	5	879	638	58.0 (55.0–60.0)
**LFIA**												
Surface protein	NA											
Nucleocapsid protein	1	101	26	80.0 (71.0–86.0)	3	206	325	39.0 (35.0–43.0)	1	80	63	56.0 (47.0–64.0)
Surface and nucleocapsid proteins	4	211	148	59.0 (53.0–64.0)	4	218	141	61.0 (55.0–66.0)	3	231	77	75.0 (70.0–80.0)
**Clinical setting**												
**ELISA**												
Inpatient only	11	792	282	74.0 (71.0–76.0)	15	1,730	355	83.0 (81.0–85.0)	6	905	165	85.0 (82.0–87.0)
Outpatient	0				7	584	61	91.0 (88.0–93.0)	0			
Inpatient and outpatient	6	560	104	84.0 (81.0–87.0)	14	1,769	148	92.0 (91.0–93.0)	6	1,266	139	90.0 (88.0–92.0)
No reported	10	1,083	463	70.0 (68.0–72.0)	39	5,073	1,153	81.0 (80.0–82.0)	11	1,227	288	81.0 (79.0–83.0)
**CLIA**												
Inpatient only	10	1,282	519	71.2 (69.0,73.3)	15	2,006	548	78.5 (76.8,80.1)	7	1,757	472	78.8 (77.1,80.5)
Outpatient	0				1	123	29		0			
Inpatient and outpatient	4	712	219	76.3 (73.4,79.0)	14	2,199	625	74.0 (72.0,75.9)	8	2,332	471	83.2 (81.8,84.6)
No reported	8	841	919	33.6 (31.0,36.3)	19	2,269	2,120	44.2 (42.6,45.8)	8	697	842	45.0 (43.0,48.0)
**LFIA**												
Inpatient only	4	216	216	50.0 (45.0–55.0)	3	201	179	53.0 (48.0–58.0)	6	1,554	1,170	57.0 (55.0–59.0)
Outpatient									4	654	4,636	12.0 (11.0–13.0)
Inpatient and outpatient	5	212	107	66.0 (61.0–72.0)	8	579	613	49.0 (46.0–51.0)	7	785	797	50.0 (47.0–52.0)
No reported	21	2,031	5,411	27.0 (26.0–28.0)	20	1,946	5,187	27.0 (26.0–28.0)	17	1,472	4,952	23.0 (22.0–24.0)
**Serological kit as index test (whether testing was by commercial kit or an in-house assay)**												
**ELISA**												
Commercial serological kit	23	2,022	703	74.0 (73.0–76.0)	66	8,167	1,731	83.0 (82.0–83.0)	21	2,632	617	81.0 (80.0–82.0)
In-house assay	6	640	238	73.0 (70.0–76.0)	13	1,220	102	92.0 (91.0–94.0)	5	965	125	89.0 (86.0–90.0)
Unclear	0				0				0			
**CLIA**												
Commercial serological kit	20	1,697	3,510	33.0 (31.0–34.0)	43	4,790	5,427	47.0 (46.0–48.0)	18	3,153	1,565	67.0 (65.0–68.0)
In-house assay	2	1,069	212	83.0 (81.0–85.0)	4	1,493	151	91.0 (89.0–92.0)	2	949	131	88.0 (86.0–90.0)
Unclear	0				2	116	68	63.0 (56.0–70.0)	0			
**LFIA**												
Commercial serological kit	28	2,397	5,148	32.0 (31.0–33.0)	25	2,489	5,100	33.0 (32.0–34.0)	30	4,263	11,073	28.0 (27.0–29.0)
In-house assay					2	696	963	42.0 (40.0–44.0)				
Unclear	4	199	700	22.0 (19.0–25.0)	4	186	830	18.0 (16.0–21.0)	4	202	414	33.0 (29.0–37.0)
**Type of specimen for RT–PCR reference test**												
**ELISA**												
Nasopharyngeal	11	575	296	66.0 (63.0–69.0)	27	3,796	934	80.0 (79.0–81.0)	10	1,322	418	76.0 (74.0–78.0)
Sputum, saliva, or oral, throat, or pharyngeal	7	484	132	79.0 (75.0–82.0)	17	1,426	291	83.0 (81.0–85.0)	6	837	273	75.0 (73.0–78.0)
Not reported	11	1,435	458	76.0 (74.0–79.0)	38	4,566	682	87.0 (86.0–88.0)	12	1,958	129	94.0 (93.0–95.0)
**CLIA**												
Nasopharyngeal	7	578	2,328	20.0 (18.0–21.0)	16	1,364	2,791	33.0 (31.0–34.0)	6	1,075	397	73.0 (71.0–75.0)
Sputum, saliva, or oral, throat, or pharyngeal	6	656	246	73.0 (70.0–76.0)	8	788	283	74.0 (71.0–76.0)	6	1,253	181	87.0 (86.0–89.0)
Not reported	13	1,688	1,325	56.0 (54.0–58.0)	30	4,872	3,711	57.0 (56.0–58.0)	11	2,436	1,218	67.0 (65.0–68.0)
**LFIA**												
Nasopharyngeal	8	992	1,366	42.0 (40.0–44.0)	8	1,004	1,272	44.0 (42.0–46.0)	13	2,180	6,808	24.0 (23.0–25.0)
Sputum, saliva, or oral, throat, or pharyngeal	9	1,020	1,301	44.0 (42.0–46.0)	9	1,124	1,305	46.0 (44.0–48.0)	8	719	5,477	12.0 (11.0–12.0)
Not reported	17	1,339	4,106	25.0 (23.0–26.0)	16	1,331	4,436	23.0 (22.0–24.0)	18	1,880	4,612	29.0 (28.0–30.0)
**Subgroup**	**IgM**				**IgG**				**IgM or IgG**			
	**No. of arms (studies)**	**TN**	**FP**	**Pooled specificity (95% CI)**	**No. of arms**	**TN**	**FP**	**Pooled specificity (95% CI)**	**No. of arms**	**TN**	**FP**	**Pooled specificity (95% CI)**
**Time post-onset**												
**ELISA**												
First week	3	440	34	93.0 (90.0–95.0)	7	27,47	129	96.0 (95.0–96.0)	4	726	64	92.0 (90.0–94.0)
Second week	4	432	13	97.0 (95.0–98.0)	8	1,064	35	97.0 (96.0–98.0)	5	9,861	14	100.0 (100.0–100.0)
Third week	6	3,535	18	99.0 (99.0–100.0)	20	8,177	597	93.0 (93.0–94.0)	2	306	0	100.0 (99.0–100.0)
Third week later (22–28 day)	1	109	3	97.0 (92.0–99.0)	6	2,749	81	97.0 (96.0–98.0)	2	224	3	99.0 (96.0–100.0)
**CLIA**												
First week	6	1,392	38	97.0 (96.0–98.0)	11	9,424	159	98.0 (98.0–99.0)	2	238	22	92.0 (87.0–95.0)
Second week	5	982	29	97.0 (96.0–98.0)	9	1,376	50	96.0 (95.0–97.0)	4	427	102	81.0 (77.0–84.0)
Third week	6	1,084	290	79.0 (77.0–81.0)	13	1,838	84	96.0 (95.0–96.0)	6	939	15	98.0 (97.0–99.0)
Third week later (22–28 day)	5	910	20	98.0 (97.0–99.0)	6	1,826	30	98.0 (98.0–99.0)	2	508	15	97.0 (95.0–98.0)
**LFIA**												
First week	5	58	42	58.0 (48.0–68.0)	5	88	9	91.0 (83.0–96.0)	16	994	52	95.0 (94.0–96.0)
Second week	3	72	12	86.0 (76.0–92.0)	3	31	24	56.0 (42.0–70.0)	6	164	30	85.0 (79.0–89.0)
Third week	12	341	58	85.0 (82.0–89.0)	12	347	135	72.0 (68.0–76.0)	9	175	52	77.0 (71.0–82.0)
Third week later (22–28 day)	2	273	2	99.0 (97.0–100.0)	2	274	1	100.0 (98.0–100.0)	7	512	20	96.0 (94.0–98.0)
**Antigen target**												
**ELISA**												
Surface protein	6	2,396	4	100.0 (100.0–100.0)	24	7,008	143	98.0 (98.0–98.0)	8	1,737	17	99.0 (98.0–99.0)
Nucleocapsid protein	7	1,075	32	97.0 (96.0–98.0)	21	4,889	125	98.0 (97.0–98.0)	2	9,651	14	100.0 (100.0–100.0)
Surface and nucleocapsid proteins	5	1,365	32	98.0 (97.0–98.0)	13	4,806	251	95.0 (94.0–96.0)	6	764	10	99.0 (98.0–99.0)
**CLIA**												
Surface protein	3	508	6	99.0 (97.0–100.0)	8	1,056	87	92.0 (91.0–94.0)	3	1,516	3	100.0 (99.0–100.0)
Nucleocapsid protein	0				5	1,319	14	99.0 (98.0–99.0)	3	1,557	2	100.0 (100.0–100.0)
Surface and nucleocapsid proteins	4	774	27	97.0 (95.0–98.0)	9	6,268	144	98.0 (97.0–98.0)	2	380	34	92.0 (89.0–94.0)
**LFIA**												
Surface protein	NA											
Nucleocapsid protein	1	37	3	93.0 (80.0–98.0)	3	52	20	72.0 (60.0–82.0)	1	75	4	95.0 (88.0–99.0)
Surface and nucleocapsid proteins	4	415	20	95.0 (93.0–97.0)	4	425	100	81.0 (77.0–84.0)	3	286	25	92.0 (88.0–95.0)
**Clinical setting**												
**ELISA**												
Inpatient only	11	2,057	70	97.0 (96.0–97.0)	15	3,964	627	86.0 (85.0–87.0)	6	1,275	3	100.0 (99.0–100.0)
Outpatient	0				7	5,641	75	99.0 (98.0–99.0)	0			
Inpatient and outpatient	6	2,083	17	99.0 (99.0–100.0)	14	5,397	234	96.0 (95.0–96.0)	6	1,470	63	96.0 (95.0–97.0)
No reported	10	3,517	44	99.0 (98.0–99.0)	39	11,119	266	98.0 (97.0–98.0)	11	12,251	88	99.0 (99.0–99.0)
**CLIA**												
Inpatient only	10	1,798	35	98.0 (97.0–99.0)	15	3,435	68	98.0 (98.0–98.0)	7	2,251	67	97.0 (96.0–98.0)
Outpatient	0				1	149	1	99.0 (96.0–100.0)	0			
Inpatient and outpatient	4	949	19	98.0 (97.0–99.0)	14	8,680	170	98.0 (98.0–98.0)	8	3,065	30	99.0 (99.0–99.0)
No reported	8	967	39	96.0 (95.0–97.0)	19	4,679	172	96.0 (96.0–97.0)	8	218	108	67.0 (61.0–72.0)
**LFIA**												
Inpatient only	4	75	47	61.0 (52.0–70.0)	3	43	14	75.0 (62.0–86.0)	6	623	53	92.0 (90.0–94.0)
Outpatient									4	247	178	58.0 (53.0–63.0)
Inpatient and outpatient	5	413	14	97.0 (95.0–98.0)	8	455	13	97.0 (95.0–99.0)	7	552	6	99.0 (98.0–100.0)
No reported	21	1,372	194	88.0 (86.0–89.0)	20	1,137	198	85.0 (83.0–87.0)	17	1,147	78	94.0 (92.0–95.0)
**Serological kit as index test (whether testing was by commercial kit or an in-house assay)**												
**ELISA**												
Commercial serological kit	23	6,288	127	98.0 (98.0–98.0)	66	23,800	1,176	95.0 (95.0–96.0)	21	12,676	144	99.0 (99.0–99.0)
In-house assay	6	1,510	20	99.0 (98.0–99.0)	13	2,360	51	98.0 (97.0–98.0)	5	1,566	10	99.0 (99.0–100.0)
Unclear	0				0				0			
**CLIA**												
Commercial serological kit	20	2,370	359	87.0 (86.0–88.0)	43	12,762	393	97.0 (97.0–97.0)	18	4,452	174	96.0 (96.0–97.0)
In-house assay	2	1,285	6	100.0 (99.0–100.0)	4	2,705	39	99.0 (98.0–99.0)	2	751	15	98.0 (97.0–99.0)
Unclear	0				2	248	2	99.0 (97.0–100.0)	0			
**LFIA**												
Commercial serological kit	28	1,841	200	90.0 (89.0–91.0)	25	1,567	194	89.0 (87.0–90.0)	30	2,529	309	89.0 (88.0–90.0)
In-house assay					2	478	23	95.0 (93.0–97.0)				
Unclear	4	27	61	31.0 (21.0–41.0)	4	47	31	60.0 (49.0–71.0)	4	40	6	87.0 (74.0–95.0)
**Type of specimen for RT–PCR reference test**												
**ELISA**												
Nasopharyngeal	11	1,073	37	97.0 (95.0–98.0)	27	10,648	346	97.0 (97.0–97.0)	10	1,412	97	94.0 (92.0–95.0)
Sputum, saliva, or oral, throat, or pharyngeal	7	875	26	97.0 (96.0–98.0)	17	7467	616	92.0 (92.0–93.0)	6	510	60	89.0 (87.0–92.0)
Not reported	11	5,866	71	99.0 (98.0–99.0)	38	13,488	339	98.0 (97.0–98.0)	12	13,642	57	100.0 (99.0–100.0)
**CLIA**												
Nasopharyngeal	7	830	294	74.0 (71.0–76.0)	16	5,900	122	98.0 (98.0–98.0)	6	699	28	96.0 (94.0–97.0)
Sputum, saliva, or oral, throat, or pharyngeal	6	1,135	22	98.0 (97.0–99.0)	8	2,671	23	99.0 (99.0–99.0)	6	2,042	124	94.0 (93.0–95.0)
Not reported	13	1,954	55	97.0 (96.0–98.0)	30	10,332	322	97.0 (97.0–97.0)	11	2,779	47	98.0 (98.0–99.0)
**LFIA**												
Nasopharyngeal	8	785	79	91.0 (89.0–93.0)	8	743	39	95.0 (93.0–96.0)	13	1,514	215	88.0 (86.0–89.0)
Sputum, saliva, or oral, throat, or pharyngeal	9	885	103	90.0 (87.0–91.0)	9	844	49	95.0 (93.0–96.0)	8	950	196	83.0 (81.0–85.0)
Not reported	17	645	130	83.0 (80.0–86.0)	16	513	160	76.0 (73.0–79.0)	18	643	65	91.0 (88.0–93.0)

### Subgroup analysis of the timing of sample collection in relation to symptom onset

The average sensitivity across all of the included studies for ELISA-tested IgG, IgM and IgG/IgM showed low sensitivity during the first week after the onset of symptoms, after which they increased in the second week and reached their highest values beyond 3 weeks. For the ELISAs, sensitivity estimates were higher in the third week or later after symptom onset (ranging from 83.0 to 90.0%). In contrast, for the CLIAs, pooled sensitivity was lower in the third week (<30%); for the LFIAs, pooled sensitivity was lower in the second week (<10%) after symptom onset. Very few studies have evaluated tests beyond 35 days to estimate accuracy. Data on specificity, as stratified by timing, showed that the pooled data were highest in the second week. Specificity was higher at least 2 weeks after symptom onset (ranging from 98.0 to 98.0%) than within the first week (ranging from 96.0 to 97.0%). For the ELISA test method, the pooled specificity of 99% (ranging from 99 to 100%) was high when the measured time post-onset was in the second week. For the CLIA and FLIA test methods, the pooled specificity was high when measured time post-onset was in the third week later (ranging from 97 to 99%) (Table 4).

### Subgroup analysis of test technology type

Point estimates for the pooled sensitivity and specificity were higher when the N protein was used. A subgroup meta-analysis showed that tests using the N antigen (ranging from 77 to 80%) were more sensitive than with the use of S protein (ranging from 66.0 to 68.0.0%) antigen tests. Moreover, IgG-based serological assays that used the N antigen were more sensitive than IgG-based serological assays that used the S antigen. For the ELISAs, specificity was higher when the nucleocapsid protein was used; however, this was not the case for the LFIAs or CLIAs. For the CLIAs, specificity and sensitivity were higher from reported studies that used the nucleocapsid proteins (ranging from 99 to 100%) (Table 4).

### Subgroup analysis of setting (outpatient vs. inpatient)

For the ELISAs, point estimates for pooled sensitivity were higher when estimates at the sample level for both inpatients and outpatients were included, in which case the sensitivity was 90% (ranging from 89 to 91%). For the LFIAs, pooled specificity was higher when estimates at the sample level for both inpatient and outpatient were included, in which case the specificity was 98% (ranging from 97 to 98%). Among the three test methods, point estimates for pooled sensitivity and specificity were higher when estimates at the sample level included both inpatients and outpatients (Table 4).

### Subgroup analysis of serological kits as index tests (whether testing was performed by using commercial kits or an in-house assay)

Both in-house and commercial kits are the preferred molecular tests being used worldwide in the COVID-19 diagnosis. We compared pooled sensitivity and specificity across subgroup according to serological kit as index test (whether testing was by commercial kit or an in-house assay). For all three of the test methods, point estimates of sensitivity and specificity were higher for in-house assays vs. commercial kits. The pooled sensitivity was higher for in-house assays (ranging from 78 to 79%) than for commercial kits (ranging from 47 to 48%). The pooled specificity was higher for in-house assays (ranging from 98 to 99%) than for commercial kits (ranging from 96 to 96%) (Table 4).

### Subgroup analysis of the type of specimen used for the RT–PCR reference test

For the ELISA and CLIA test methods, pooled specificity and sensitivity were high when the types of specimens that were used for the RT–PCR were sputum, saliva, oral, throat or pharyngeal samples. However, when the sample was nasopharyngeal, the pooled sensitivity and specificity were high, as indicated by the LFIA test method (Table 4).

### The accuracy of serological tests world map for COVID-19

We pooled the sensitivity and specificity of COVID-19 serological tests that are used worldwide. For the ELISA, pooled sensitivity was higher in Canada (100%) than in other areas; For the CLIA, pooled sensitivity was higher in Croatia (97%) than in other areas (Supplementary Figures S5–S7). Among these three serological tests, ELISA exhibited higher sensitivity (ranging from 50 to 100%) and higher specificity (ranging from ≥73–100%). For the CLIA, Italy, Switzerland and Singapore had lower sensitivities (< 30%) (Supplementary Table S3). Among the three test methods, point estimates for pooled specificity were higher in Latin America (ranging from 99.0 to 100%) (Supplementary Figures S4–S9, Supplementary Table S3).

## Discussion

In this systematic review and meta-analysis, we found that ELISA and CLIA methods performed better in terms of sensitivity than the LFIA method, thus indicating that viral infections can lead to false-positive results for the LIFA method. For each test method, the type of immunoglobulin being measured (IgM, IgG or both) was associated with diagnostic accuracy, and sensitivities were consistently higher with IgG than with IgM. Moreover, IgG-IgM-based CLIA tests exhibited the best overall diagnostic test accuracy. Moreover, pooled specificities of each test method were high. Pooled sensitivities and specificities were higher with in-house assays vs. commercial kits and in the third week or later, compared with the first and second weeks after symptom onset. Additionally, point estimates for pooled sensitivity and specificity were higher when estimates at the sample level were both inpatient and outpatient; therefore, serological tests are able to detect lower antibody levels that are likely observed with milder and asymptomatic COVID-19 disease.

### Research implications

1. For all three of the methods, the LFIA method had lower sensitivity than the ELISA or CLIA methods for IgM (similar data were available for IgG and IgM/IgG). For the LFIAs, pooled sensitivity was lowest in the second week of symptom onset and highest in the first week. These observations can provide recommendations to the World Health Organization for improving test accuracy when using LFIA serological tests. Given the poor performances of the current LFIA devices (7, 16), LFIA tests for COVID-19 in the second week of symptom onset (with an average sensitivity of 9%) will be falsely identified as not being positive for infection. In addition, sensitivity estimates are likely to increase in the first week, compared with other time points of sample collection. Our time-stratified analyses suggest that LFIA seems to be a better choice (in terms of sensitivity) at the first week of sample collection, in relation to symptom onset.

2. For all three of the test methods, pooled sensitivities and specificities were higher with in-house assays vs. commercial kits. These findings are expected, given that the pooled sensitivities were lower with the commercial kits than with in-house assays (7). Point estimates of pooled sensitivity were lower for commercial kits vs. in-house assays, for all three methods, with the strongest difference seen for LFIAs, where the sensitivity of commercial kits was 28.0% sensitivity and 89.0% specificity with IgM or IgG. For commercial kits based LFIA, the sensitivity was found to be below 50% and higher quality clinical studies assessing the diagnostic accuracy of commercial kits based LFIA are urgently needed.

3. Sensitivity varied with the time since the onset of symptoms and technology test methods. Our findings should give pause to governments that are contemplating the use of serological tests. For example, if LFIAs are applied to a population in the second week after the onset of symptoms, the average sensitivity of the test may be 9%; thus, only 9 patients out of 100 true positive patients can be detected. Serological tests are likely to have a useful role in detecting previous COVID-19 infections if they are used at 15 or more days after the onset of symptoms, except with estimated pooled specificities using CLIAs and LFIAs test methods, which are more suitable for use at 7 days after the onset of symptoms. Overall, the type of sample should be collected with consideration of the timing of the infection. It is necessary to perform the correct test at the correct time in the sample collection process, in order to avoid misdiagnoses of asymptomatic patients who are negative for serological tests.

4. Sensitivity has mainly been evaluated in hospitalized patients (7, 10); therefore, it is unclear whether the tests are able to detect lower antibody levels that are likely observed with milder and asymptomatic COVID-19 disease. Few studies have solely evaluated outpatient sensitivity accuracy. Point estimates for pooled sensitivity and specificity were higher when estimates at the sample level included both inpatients and outpatients. Our findings support the use of serological tests that are applied to people with mild symptoms who were not hospitalized, thus reducing variability in the estimates and enhancing generalizability.

5. There was little clear evidence of differences in specificity between the technology types. Specifically, all of the tests displayed high specificities. Within each class of immunoglobulin, specificity was lowest for the IgM-based CLIA tests.

6. Generally, IgG-based serological tests demonstrated a better choice in terms of sensitivity than IgM-based serological assays in each respective test method. IgG-based tests may be a safer choice at this stage of the pandemic. Low IgM antibody concentrations could potentially be explained by the fact that, immediately after a person is infected, antibodies may not have been developed yet; additionally, if it is too late after a person has been infected, IgM antibodies may have decreased or disappeared (17). The nucleocapsid protein and surface protein were used for detecting IgM and IgG antibodies, and their diagnostic feasibilities were evaluated. A subgroup meta-analysis showed that nucleocapsid antigen-based IgG serological assays are more sensitive than S antigen-based IgG serological assays that use the S antigen, thus indicating that combined IgG/IgM test antigen target nucleocapsid protein-based CLIA tests have the best overall diagnostic test accuracy.

### Comparison to previous studies

The sensitivities of all of the serological assays varied widely across the studies. Similar to other meta-analyses (7, 16, 18), the LFIA method had lower sensitivities than the CLIA and ELISA methods within each antibody class. CLIA and ELISA may be a safer choice at this stage of the pandemic. In addition, similar to other meta-analyses (17), IgM-based serological assays had the lowest sensitivities, compared with IgG-based serological tests, in each respective test method. From this study, we showed that IgG-IgM-based CLIA tests had a higher pooled sensitivity than the ELISA and LFIA tests. Moreover, it must be noted that a meta-analysis by Vengesai et al. (16) found that IgG-IgM-based ELISA tests have the best overall diagnostic test accuracy; however, in that review, they did not estimate the pooled sensitivity of IgG-IgM-based CLIA, due to the limited number of studies.

Few studies have evaluated tests beyond 35 days to estimate accuracy. For ELISAs, sensitivity estimates were higher in the third week or later after the onset of symptoms (ranging from 88 to 90%). In contrast, for the CLIAs, pooled sensitivity was lower in the third week (< 35%); For LFIAs, pooled sensitivity was lower in the second week (< 10%) after symptom onset. These findings differ from those of previous studies, in which sensitivity estimates were lowest in the first week of symptom onset and highest in the third week or later (7, 10). These observations argue against the use of serological tests for COVID-19 that exhibit higher sensitivity when performed later during the course of the disease.

A subgroup meta-analysis showed that tests using the nucleocapsid antigen were more sensitive than surface antigen tests in each immunoglobulin (IgM, IgG or both) test method. The pooled sensitivity results are in agreement with other meta-analyses that demonstrated that IgG-based serological assays that use the N antigen are more sensitive than IgG-based serological assays that use the S antigen (17). However, it must be noted that a meta-analysis by Liu et al. (19) showed that the S antigen is more sensitive than IgM-based serological assays that used N antigen tests. Thus, there is a need for more research concerning a higher sensitivity and earlier immune response to the nucleocapsid antigen.

### Strengths and limitations of this review

Our review had several strengths. For example, our review involved two independent reviewers who systematically assessed potential sources of bias. Additionally, the entire search strategy and data analysis process were relatively standardized. Moreover, we included 134 published articles on SARS-CoV-2 infections that were defined by RT–PCR because a considerable amount of new research is being published in this field. The advantages of large studies and large sample sizes allow researchers to magnify the bias associated with error, which can result from sampling or study design. Another strength of our review was that the study was conducted using in-depth subgroup meta-analyses to evaluate potential sources of heterogeneity in sensitivity and specificity, which reduces variability in the estimates and enhances diagnostic accuracy.

Our study also had some limitations. For example, we did not pool sensitivity and specificity for measurements of IgA or total immunoglobulin levels, due to small numbers. Another limitation was that we did not search for studies from individuals who were not suspected of having COVID-19 or specimens from individuals with COVID-19 symptoms and a negative RT–PCR result for SARS-CoV-2.

## Conclusions

Seroconversion occurred after 7 days in 50% of patients (and by day 14 in all of the patients), but this was not followed by a rapid decline in viral load (20). There is an urgent need for an effective and accurate diagnostic method to limit the spread of the COVID-19 infection. At present, rapid antigen or antibody tests, immunoenzymatic serological tests and molecular tests based on RT–PCR are the most widely used and validated techniques worldwide (21). We have found major weaknesses in the evidence base for serological tests for COVID-19. It is necessary to take into account not only the right test method (ELISAs, LFIAs, or CLIAs) but also the correct time from the onset of symptoms and from the correct biological sample for a successful outcome of the diagnostic test. Due to the limitations of serological tests, other techniques, including isothermal nucleic acid amplification techniques, clusters of regularly interspaced short palindromic repeats/Cas (CRISPR/Cas)-based approaches or digital PCR methods, should be quickly approved to provide guidance for a correct diagnosis of COVID-19.

## Author contributions

XZ and RD: drafting and revision of the manuscript for content, including medical writing for content, analysis or interpretation of data, and major role in the acquisition of data. FG, XW, YD, RC, and ML: major role in the acquisition of data. CT and LL: study concept or design. All authors contributed to the article and approved the submitted version.

## Supplementary material

Supplementary Figure S1 Visual inspection of summary ROC curves by test method and antibody class.

Supplementary Figure S2 Meta-analytical estimates of sensitivity (with 95%) by serological test method and antibody class.

Supplementary Figure S3 Meta-analytical estimates of specificity (with 95%) by serological test method and antibody class.

Supplementary Figure S4 Sensitivity of ELISA around the world.

Supplementary Figure S5 Sensitivity of CLIA around the world.

Supplementary Figure S6 Sensitivity of LFIA around the world.

Supplementary Figure S7 Specificity of ELISA around the world.

Supplementary Figure S8 Specificity of CLIA around the world.

Supplementary Figure S9 Specificity of LFIA around the world.

Supplementary Table S1 The included study individual QUADAS-2 evaluations.

Supplementary Table S2 Report the PubMed ID for each included study.

Supplementary Table S3 The accuracy of serological tests for COVID-19 around the world.
