# Supplementary material for: Accuracy of serological tests for COVID-19: A systematic review and meta-analysis
Source: Front Public Health. 2022 Dec 16;10:923525. doi: 10.3389/fpubh.2022.923525 (PMC9800917; doi:10.3389/fpubh.2022.923525)
Supplement: Supplementary Figure S1 — Visual inspection of summary ROC curves by test method and antibody class. [file Data_Sheet_1.pdf]

Directory

1.Supplementary Methods S1 Search strategy 3
2.Supplementary figure S1 visual inspection of summary ROC curves by test method and antibody class--ROC curves of ELISA IgG 4
3.Supplementary figure S1 visual inspection of summary ROC curves by test method and antibody class--ROC curves of ELISA IgM 5
4.Supplementary figure S1 visual inspection of summary ROC curves by test method and antibody class--ROC curves of ELISA IgG or IgM 6
5.Supplementary figure S1 visual inspection of summary ROC curves by test method and antibody class--ROC curves of CLIA IgG 7
6.Supplementary figure S1 visual inspection of summary ROC curves by test method and antibody class--ROC curves of CLIA IgM 8
7.Supplementary figure S1 visual inspection of summary ROC curves by test method and antibody class--ROC curves of CLIA IgG or IgM 9
8.Supplementary figure S1 visual inspection of summary ROC curves by test method and antibody class--ROC curves of LFIA IgG 10
9.Supplementary figure S1 visual inspection of summary ROC curves by test method and antibody class--ROC curves of LFIA IgM 11
10.Supplementary figure S1 visual inspection of summary ROC curves by test method and antibody class--ROC curves of LFIA IgG or IgM 12
11.Supplementary figure S2 Meta-analytical estimates of sensitivity(with 95%)by serological test method and antibody class--Forest plot of ELISA IgG sensitivity 13
12.Supplementary figure S2 Meta-analytical estimates of sensitivity(with 95%)by serological test method and antibody class--Forest plot of ELISA IgM sensitivity 14
13.Supplementary figure S2 Meta-analytical estimates of sensitivity(with 95%)by serological test method and antibody class--Forest plot of ELISA IgG or IgM sensitivity 15
14.Supplementary figure S2 Meta-analytical estimates of sensitivity(with 95%)by serological test method and antibody class--Forest plot of CLIA IgG sensitivity 16
15.Supplementary figure S2 Meta-analytical estimates of sensitivity(with 95%)by serological test method and antibody class--Forest plot of CLIA IgM sensitivity 17
16.Supplementary figure S2 Meta-analytical estimates of sensitivity(with 95%)by serological test method and antibody class--Forest plot of CLIA IgG or IgM sensitivity 18
17.Supplementary figure S2 Meta-analytical estimates of sensitivity(with 95%)by serological test method and antibody class--Forest plot of LFIA IgG sensitivity 19
18.Supplementary figure S2 Meta-analytical estimates of sensitivity(with 95%)by serological test method and antibody class--Forest plot of LFIA IgM sensitivity 20
19.Supplementary figure S2 Meta-analytical estimates of sensitivity(with 95%)by serological test method and antibody class--Forest plot of LFIA IgG or IgM sensitivity 21
20.Supplementary figure S3 Meta-analytical estimates of specificity (with 95%)by serological test method and antibody class--ELISA IgG specificity 22
21.Supplementary figure S3 Meta-analytical estimates of specificity (with 95%)by serological test method and antibody class--ELISA IgM specificity 23
22.Supplementary figure S3 Meta-analytical estimates of specificity (with 95%)by serological test

method and antibody class--ELISA IgG or IgM specificity 24
23.Supplementary figure S3 Meta-analytical estimates of specificity (with 95%)by serological test method and antibody class--CLIA IgG specificity 25
24.Supplementary figure S3 Meta-analytical estimates of specificity (with 95%)by serological test method and antibody class--CLIA IgM specificity 26
25.Supplementary figure S3 Meta-analytical estimates of specificity (with 95%)by serological test method and antibody class--CLIA IgG or IgM specificity 27
26.Supplementary figure S3 Meta-analytical estimates of specificity (with 95%)by serological test method and antibody class--LFIA IgG specificity 28
27.Supplementary figure S3 Meta-analytical estimates of specificity (with 95%)by serological test method and antibody class--LFIA IgM specificity 29
28.Supplementary figure S3 Meta-analytical estimates of specificity (with 95%)by serological test method and antibody class--LFIA IgG or IgM specificity 30
29.Supplementary S4 Sensitivity of ELISA Around the World 31
30.Supplementary S5 Sensitivity of CLIA Around the World 32
31.Supplementary S6 Sensitivity of LFIA Around the World 33
32.Supplementary S7 Specificity of ELISA Around the World 34
33.Supplementary S8 Specificity of CLIA Around the World 35
34.Supplementary S9 Specificity of LFIA Around the World 36
35.Supplementary table S1 37
36. supplementary table S2 44
37. Supplementtary table S3 60

1.Supplementary Methods S1 Search strategy

Pubmed:

#1 ((COVID-19[Supplementary Concept]) OR (2019 novel coronavirus disease[Title/Abstract]) OR (COVID19[Title/Abstract])
OR (COVID-19 pandemic[Title/Abstract])
OR (SARS-CoV-2 infection[Title/Abstract])
OR (COVID-19 virus disease[Title/Abstract])
OR (2019 novel coronavirus infection[Title/Abstract])
OR (2019-nCoV infection[Title/Abstract])
OR (coronavirus disease 2019[Title/Abstract])
OR (coronavirus disease-19[Title/Abstract])
OR (2019-nCoV disease[Title/Abstract])
OR (COVID-19 virus infection[Title/Abstract]))
OR (Wuhan coronavirus[Title/Abstract]))

#2 ((Immunoglobulin M[Mesh]) OR (IgM[Title/Abstract] OR (Immunoglobulin G[Mesh]) OR (IgG[Title/Abstract]))

#3 (animals [MeSH Terms] OR (editorial[Publication Type] OR comment[Publication Type] OR letter[Publication Type] OR newspaper article[Publication Type])

#4 #1 AND #2 NOT #3

Embase:

('coronavirus disease 2019'/exp OR 'covid-19':ab,ti OR '2019 novel coronavirus disease':ab,ti OR 'covid19':ab,ti OR 'covid-19 pandemic':ab,ti OR 'sars-cov-2 infection':ab,ti OR 'covid-19 virus disease':ab,ti OR '2019 novel coronavirus infection':ab,ti OR '2019-ncov infection':ab,ti OR 'coronavirus disease-19':ab,ti OR '2019-ncov disease':ab,ti OR 'covid-19 virus infection':ab,ti OR 'wuhan coronavirus':ab,ti) AND ('immunoglobulin m'/exp OR 'igm':ab,ti OR 'immunoglobulin g'/exp OR 'igg':ab,ti) NOT (editorial:it OR letter:it OR 'newspaper article':it)

Cochrane Database of Systematic Reviews

#1 (coronavirus disease 2019):ti,ab,kw OR (covid-19):ti,ab,kw OR (2019 novel coronavirus disease):ti,ab,kw OR (covid19):ti,ab,kw OR (covid-19 pandemic):ti,ab,kw OR (sars-cov-2 infection):ti,ab,kw OR (covid-19 virus disease):ti,ab,kw OR (2019 novel coronavirus infection):ti,ab,kw OR (coronavirus disease-19):ti,ab,kw OR (covid-19 virus infection):ti,ab,kw OR (wuhan coronavirus):ti,ab,kw

#2 MeSH descriptor: [Immunoglobulin G] explode all trees

#3 ("IgG"):ti,ab,kw

#4 MeSH descriptor: [Immunoglobulin M] explode all trees

#5 ("IgM"):ti,ab,kw

#6 #2 OR #3 OR #4 OR #5

#7 #1 AND #6

2.Supplementary figure S1 visual inspection of summary ROC curves by test method and antibody class--ROC curves of ELISA IgG

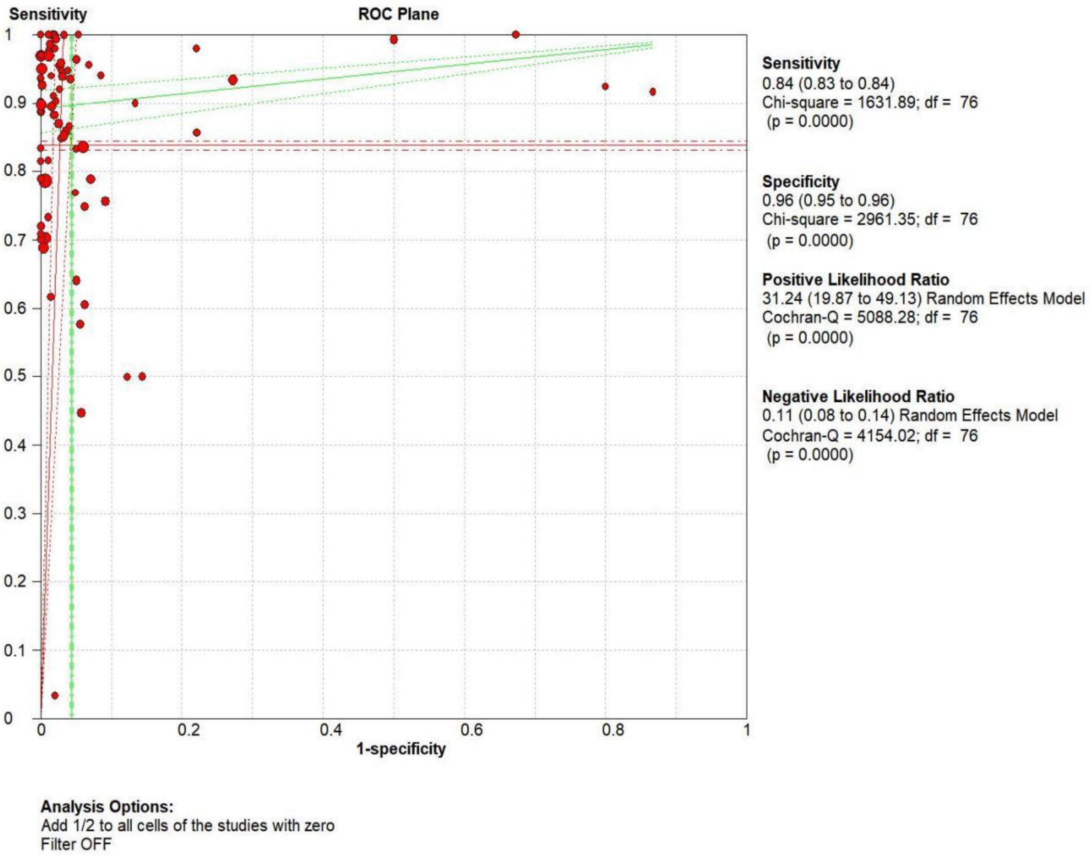

3.Supplementary figure S1 visual inspection of summary ROC curves by test method and antibody class--ROC curves of ELISA IgM

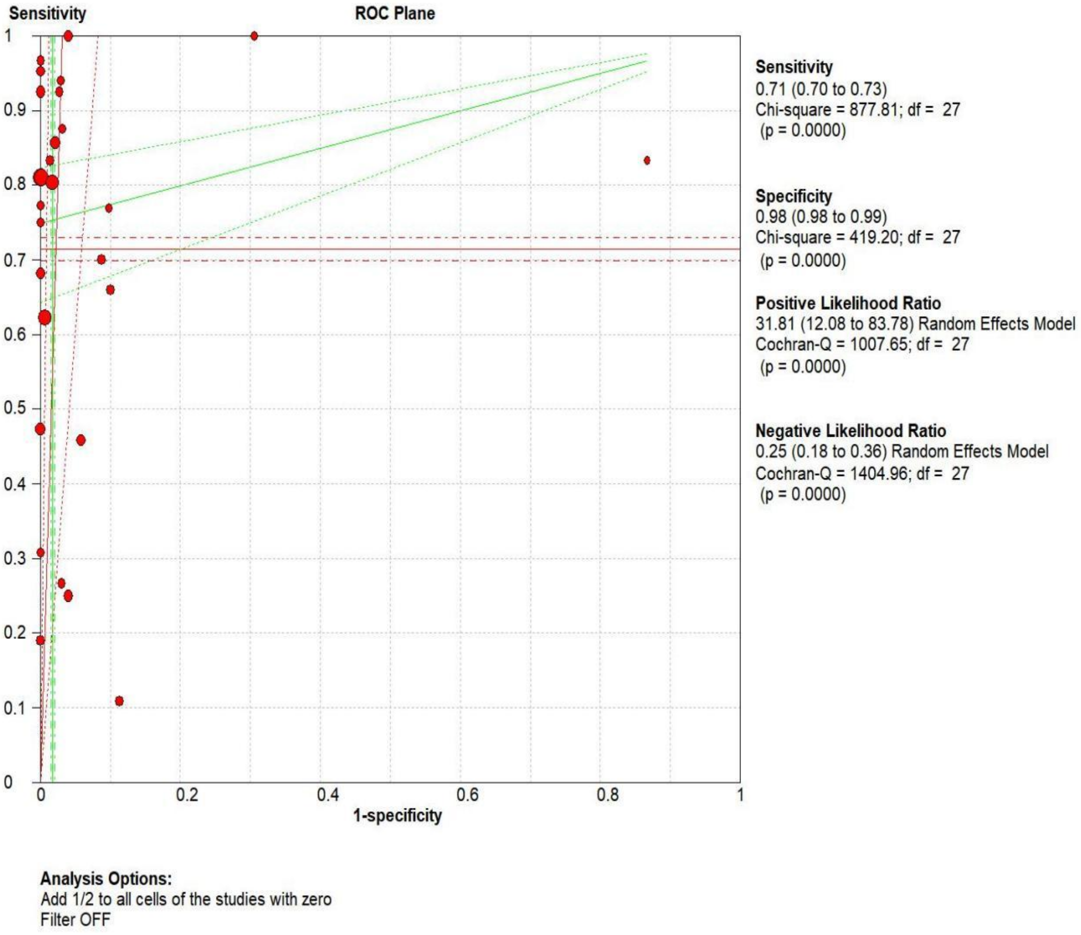

4.Supplementary figure S1 visual inspection of summary ROC curves by test method and antibody class--ROC curves of ELISA IgG or IgM

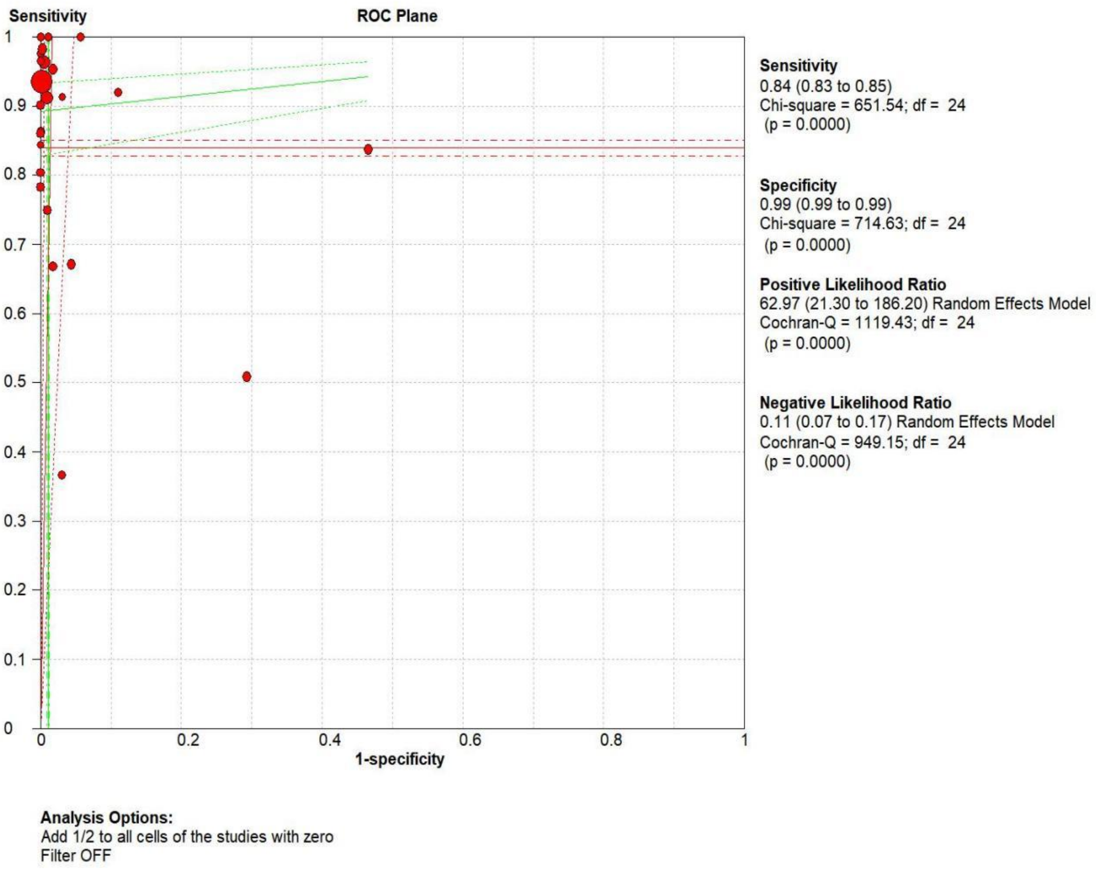

5.Supplementary figure S1 visual inspection of summary ROC curves by test method and antibody class--ROC curves of CLIA IgG

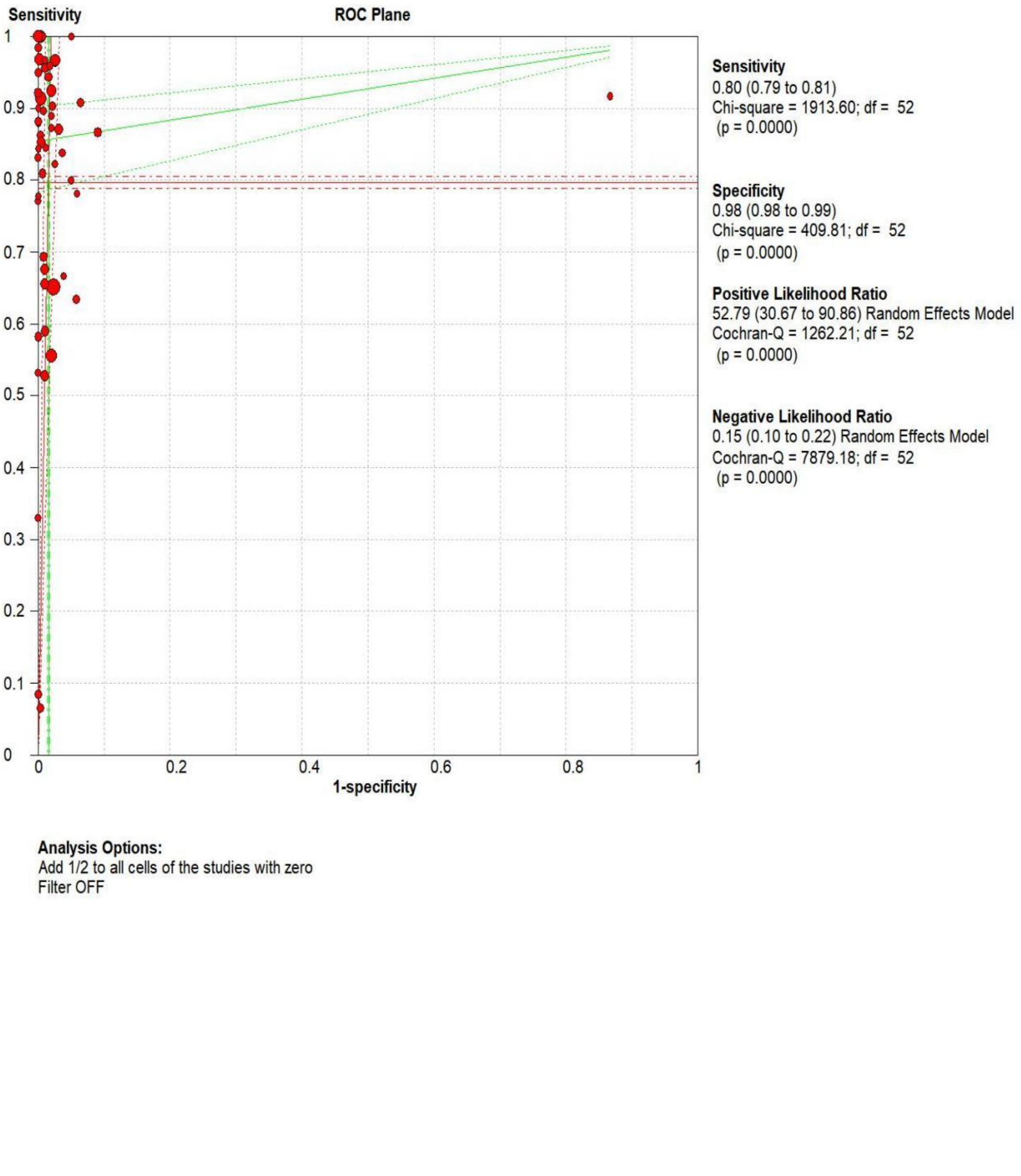

6.Supplementary figure S1 visual inspection of summary ROC curves by test method and antibody class--ROC curves of CLIA IgM

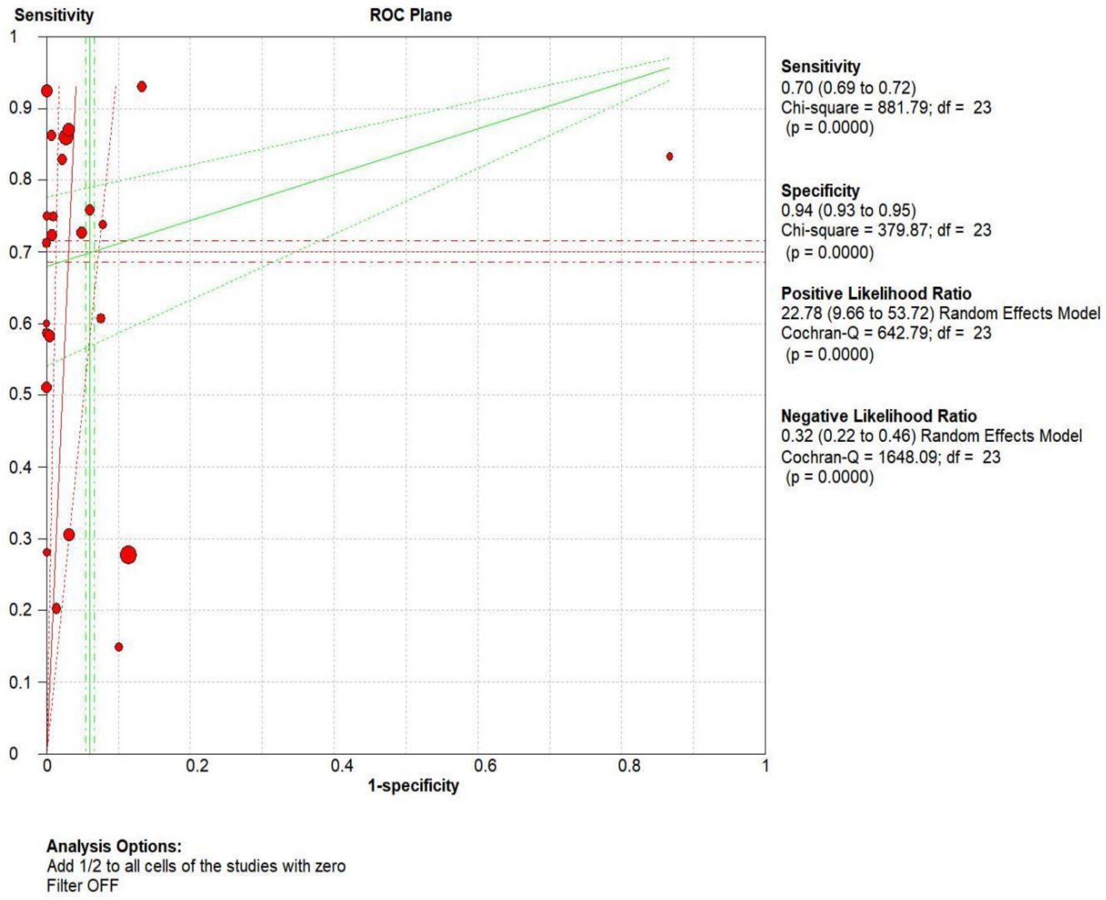

7.Supplementary figure S1 visual inspection of summary ROC curves by test method and antibody class--ROC curves of CLIA IgG or IgM

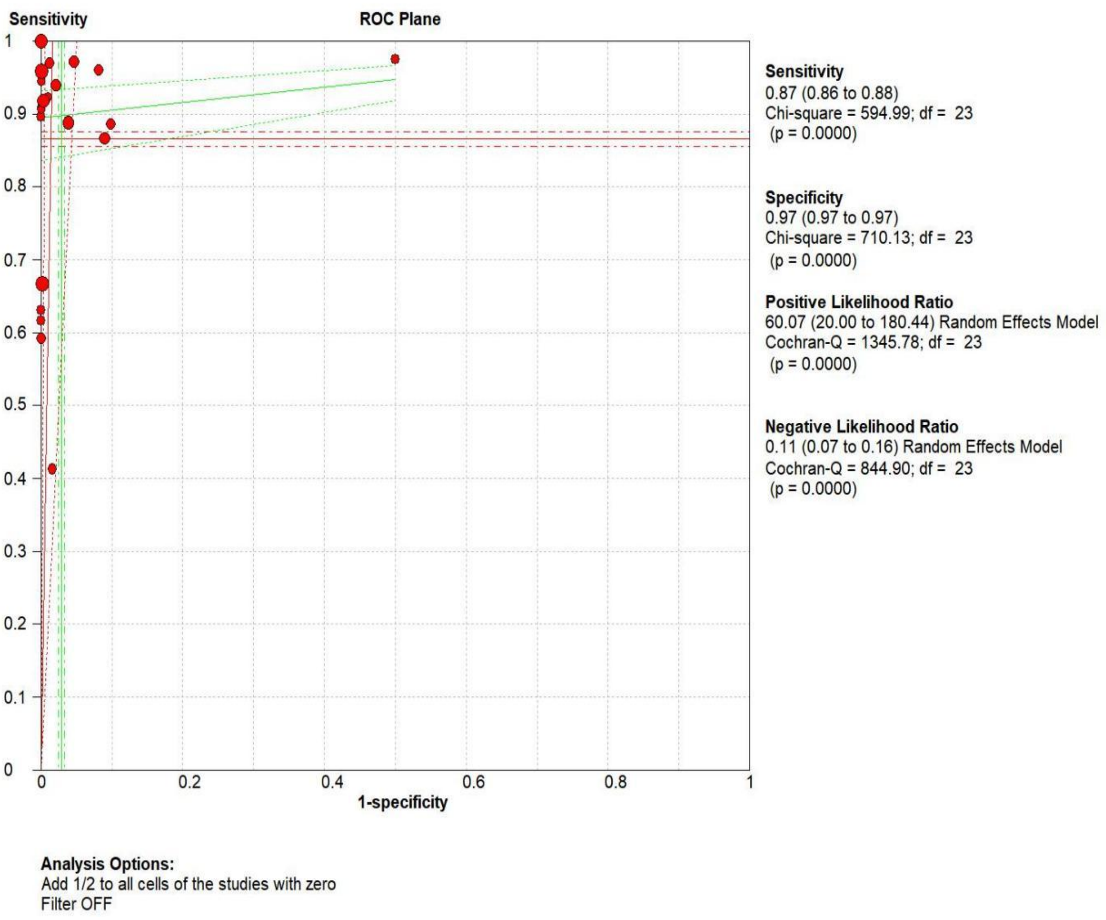

8.Supplementary figure S1 visual inspection of summary ROC curves by test method and antibody class--ROC curves of LFIA IgG

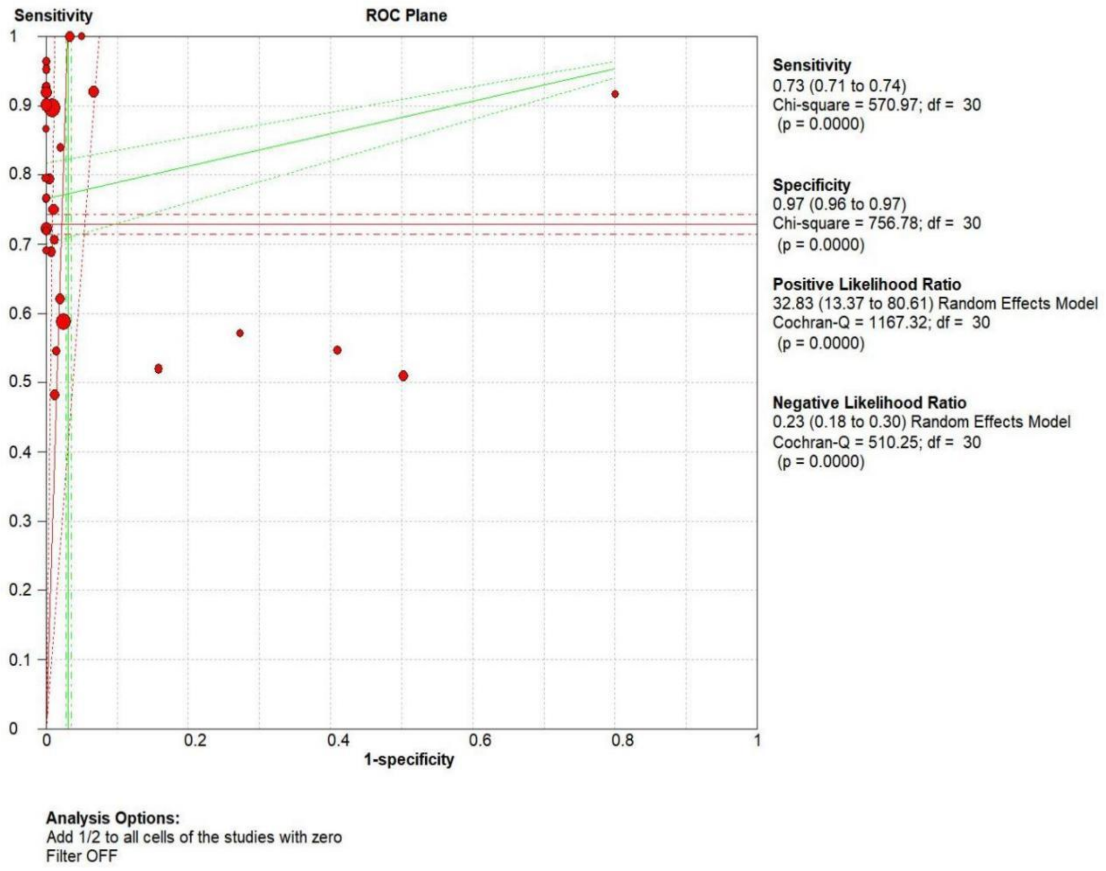

9.Supplementary figure S1 visual inspection of summary ROC curves by test method and antibody class--ROC curves of LFIA IgM

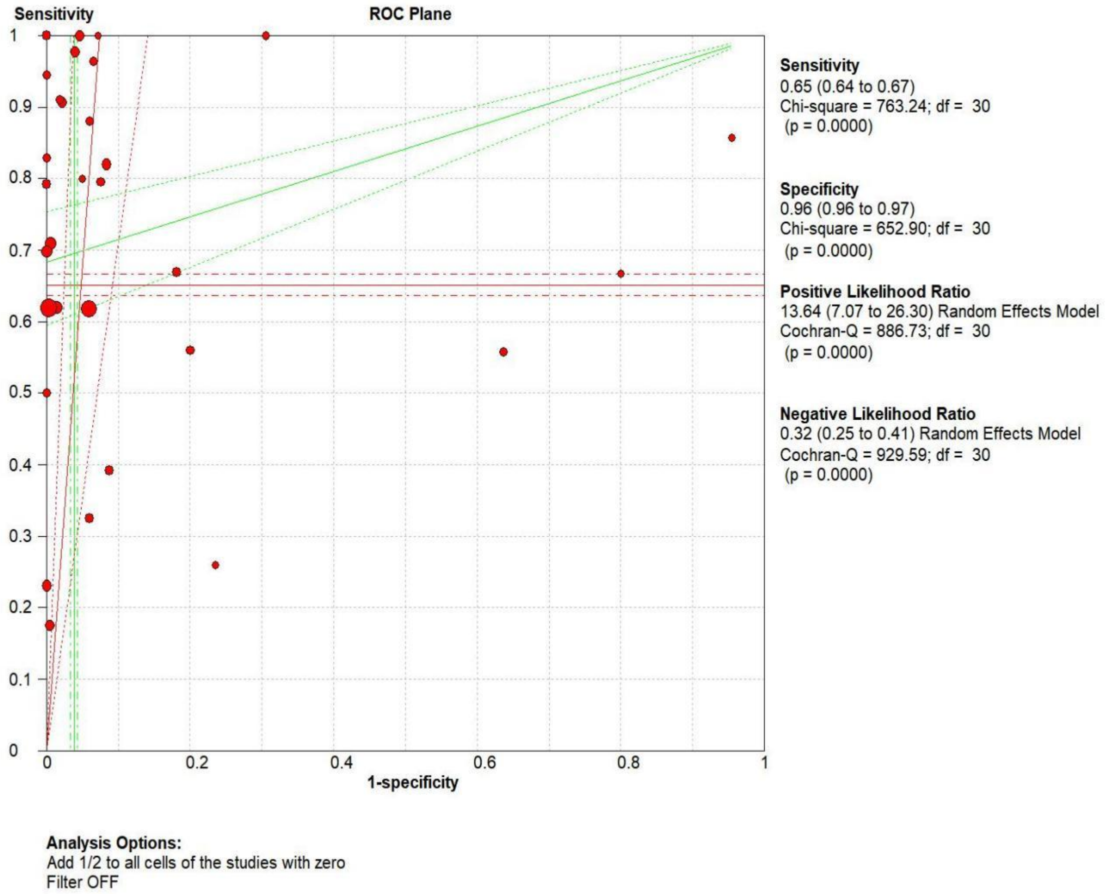

10.Supplementary figure S1 visual inspection of summary ROC curves by test method and antibody class--ROC curves of LFIA IgG or IgM

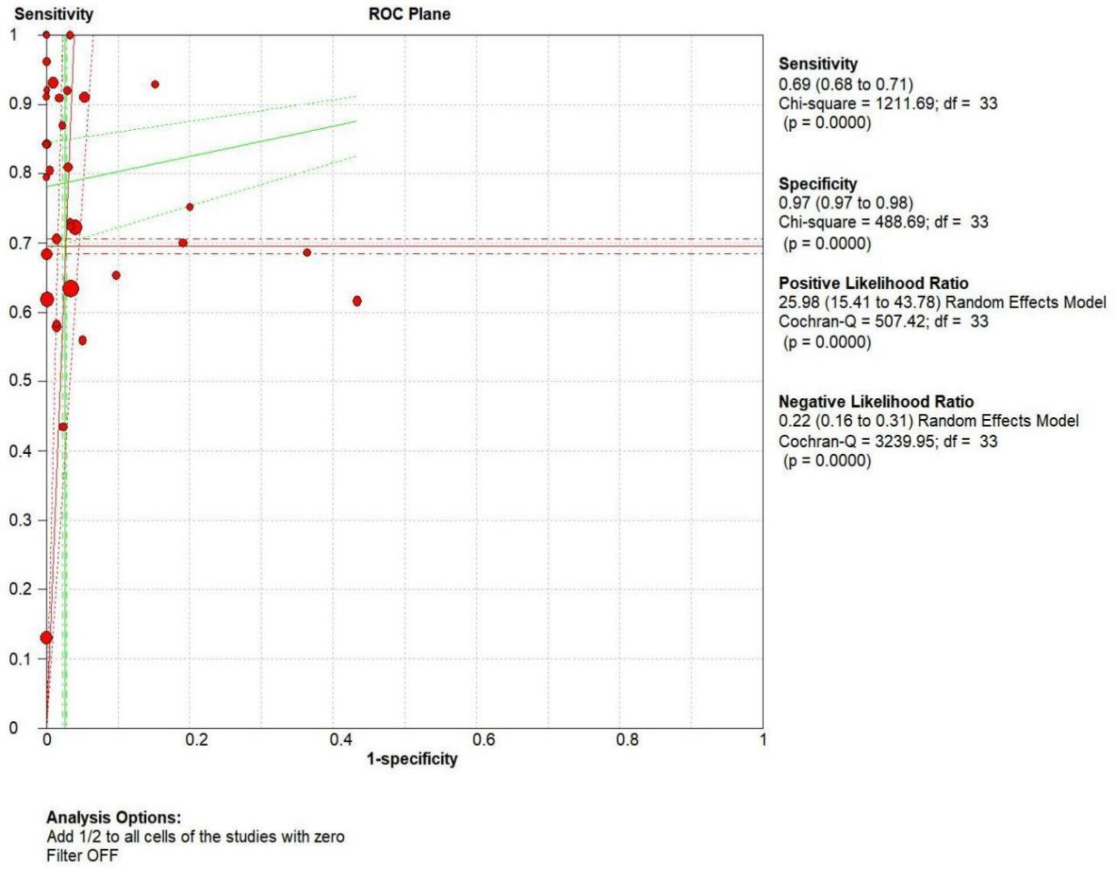

11.Supplementary figure S2 Meta-analytical estimates of sensitivity(with 95%)by serological test method and antibody class--Forest plot of ELISA IgG sensitivity

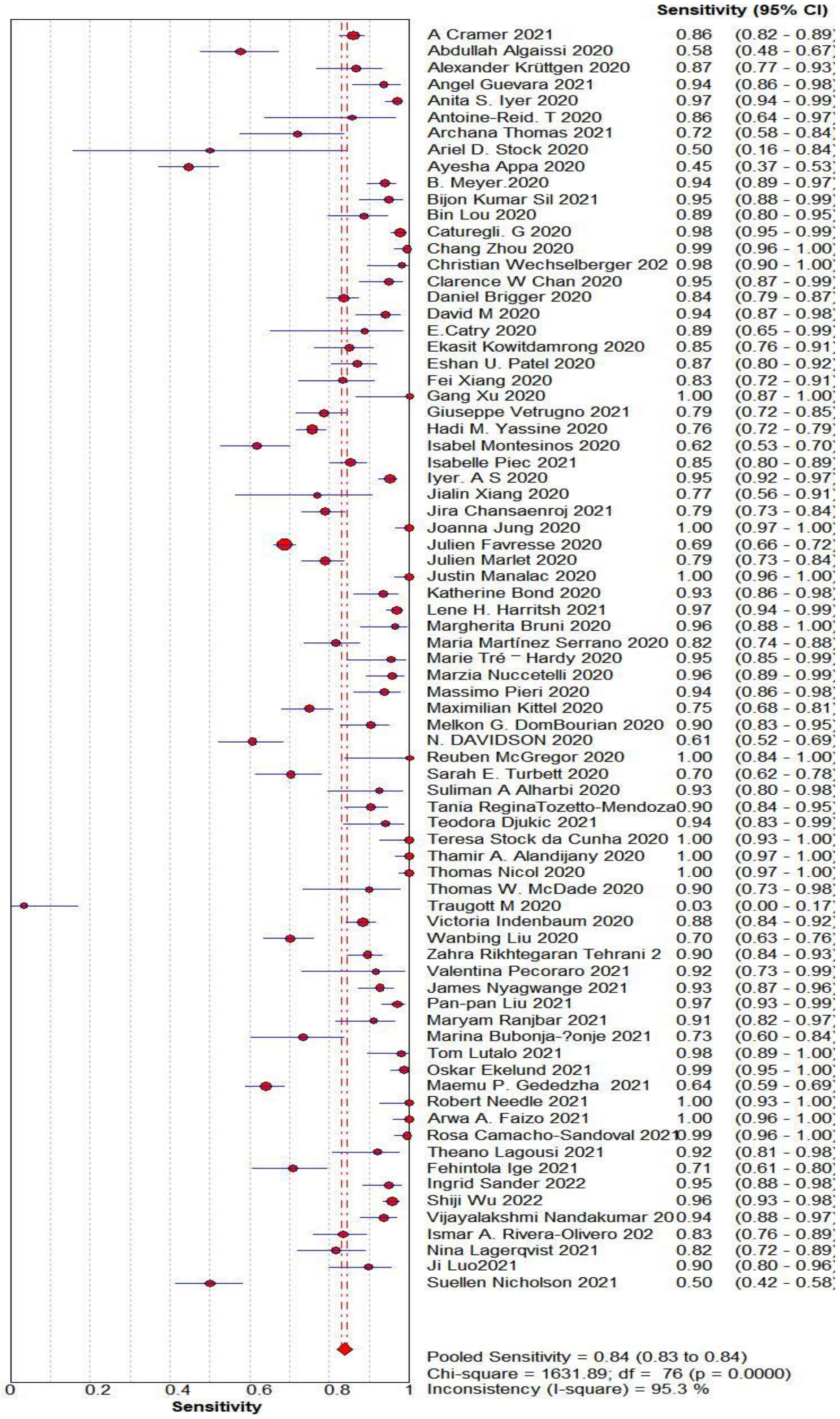

12.Supplementary figure S2 Meta-analytical estimates of sensitivity(with 95%)by serological test method and antibody class--Forest plot of ELISA IgM sensitivity

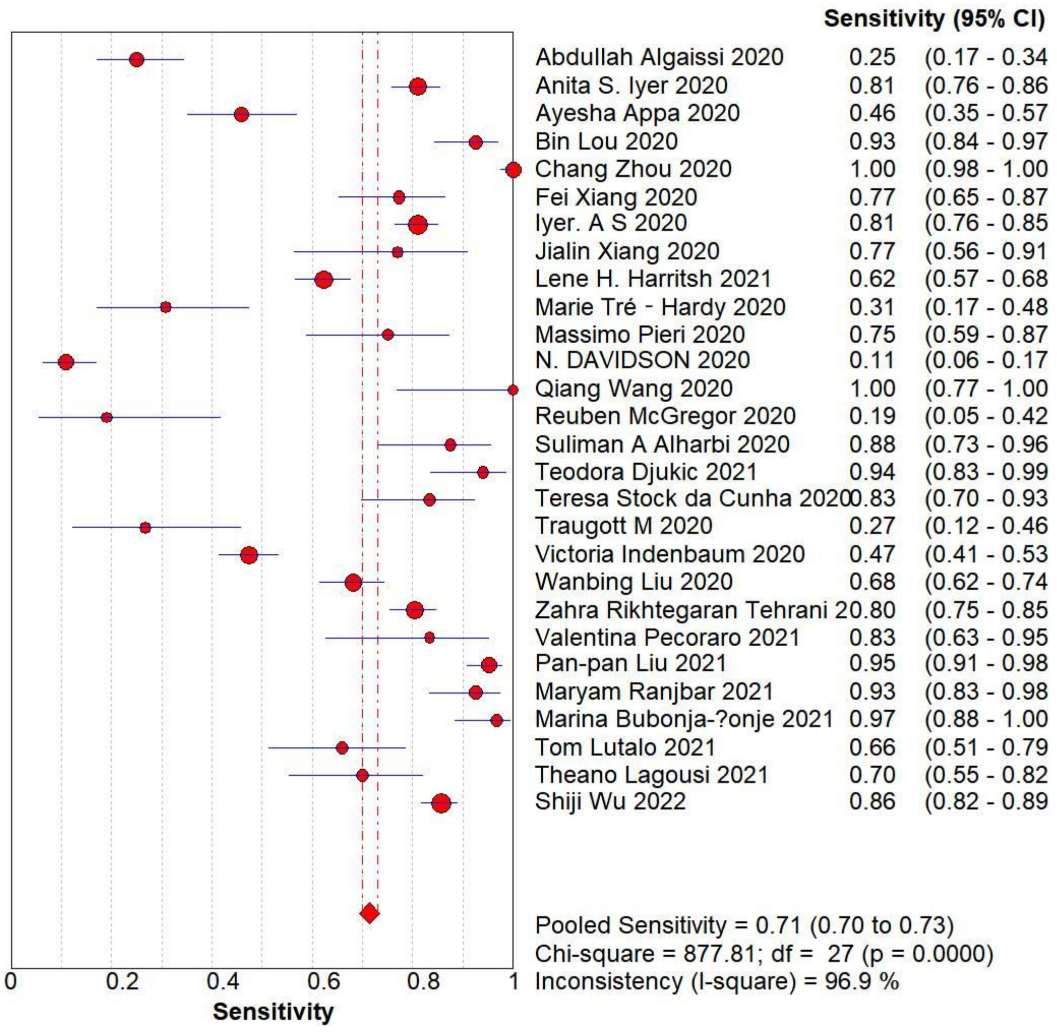

13.Supplementary figure S2 Meta-analytical estimates of sensitivity(with 95%)by serological test method and antibody class--Forest plot of ELISA IgG or IgM sensitivity

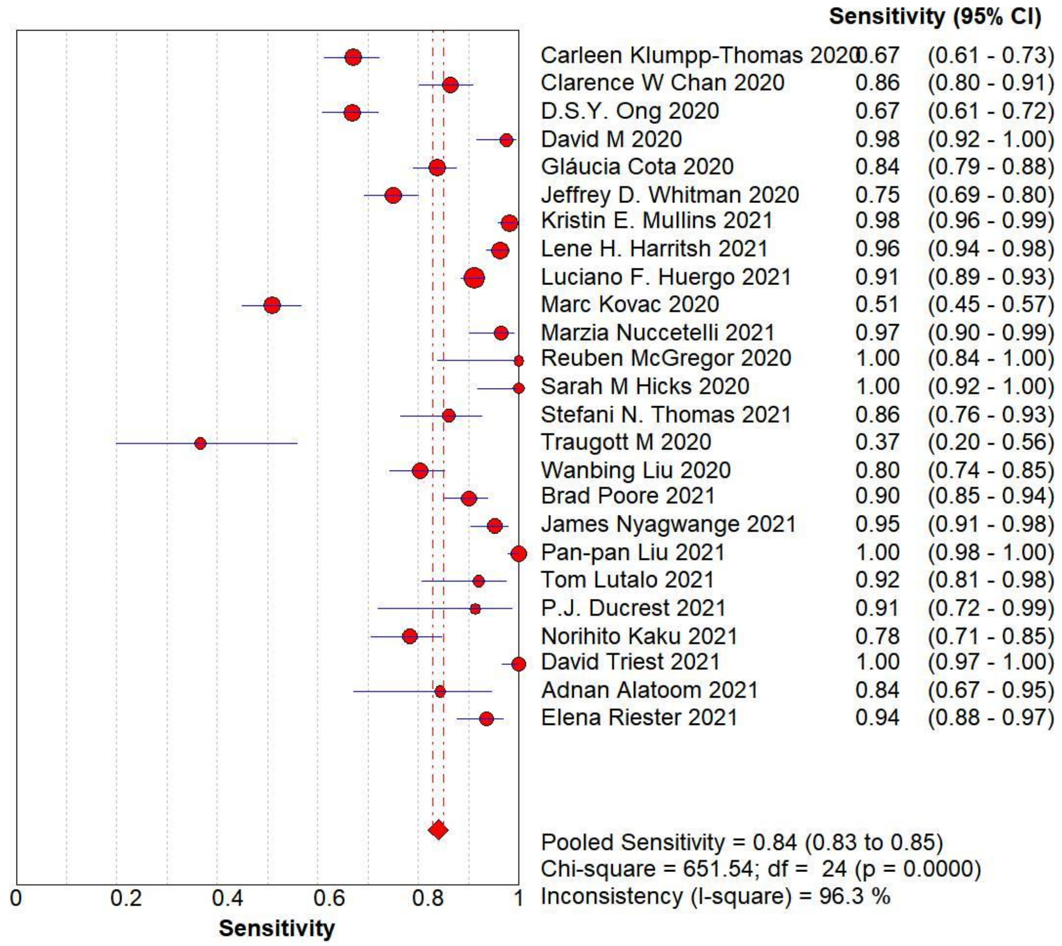

14.Supplementary figure S2 Meta-analytical estimates of sensitivity(with 95%)by serological test method and antibody class--Forest plot of CLIA IgG sensitivity

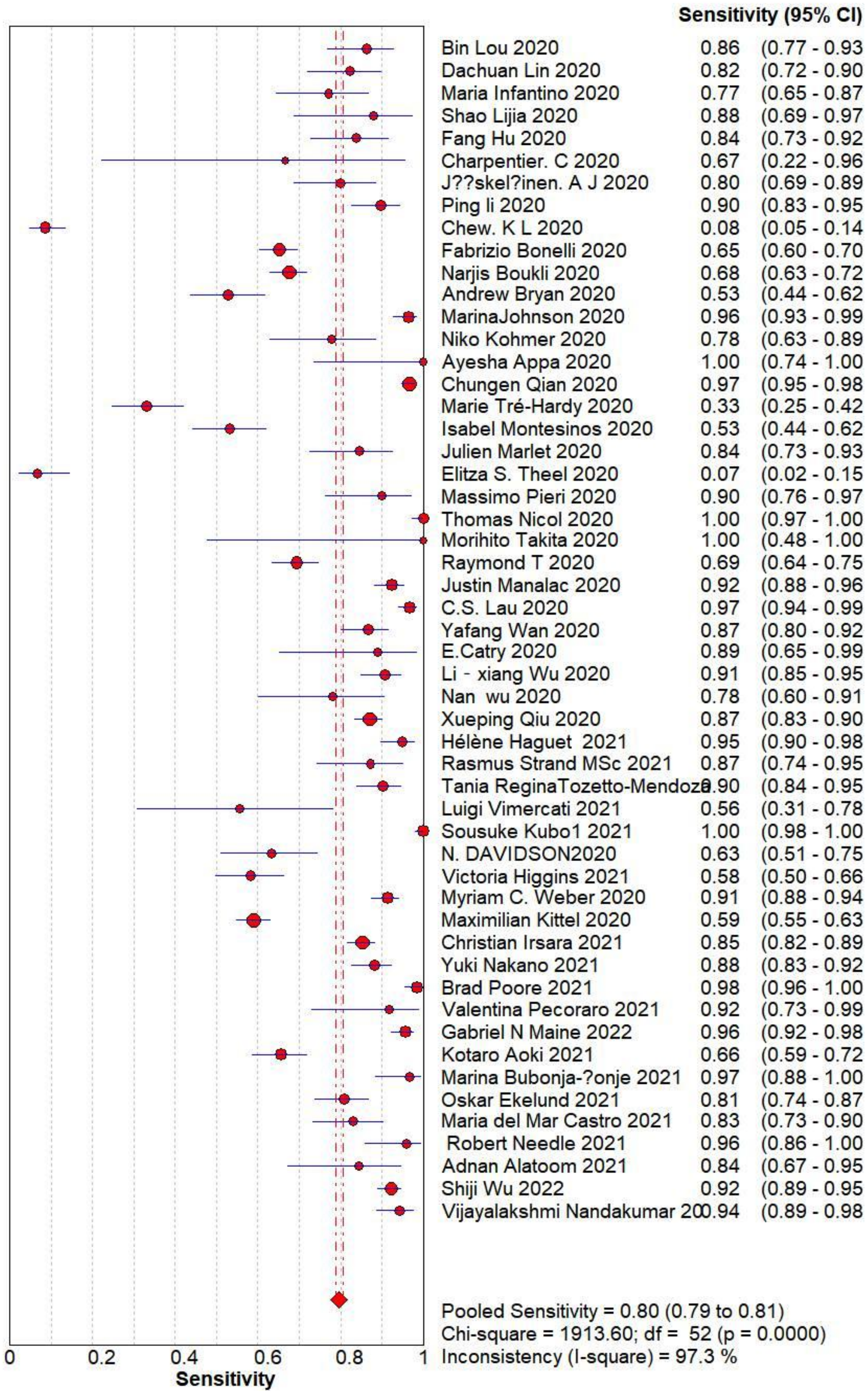

15.Supplementary figure S2 Meta-analytical estimates of sensitivity(with 95%)by serological test method and antibody class--Forest plot of CLIA IgM sensitivity

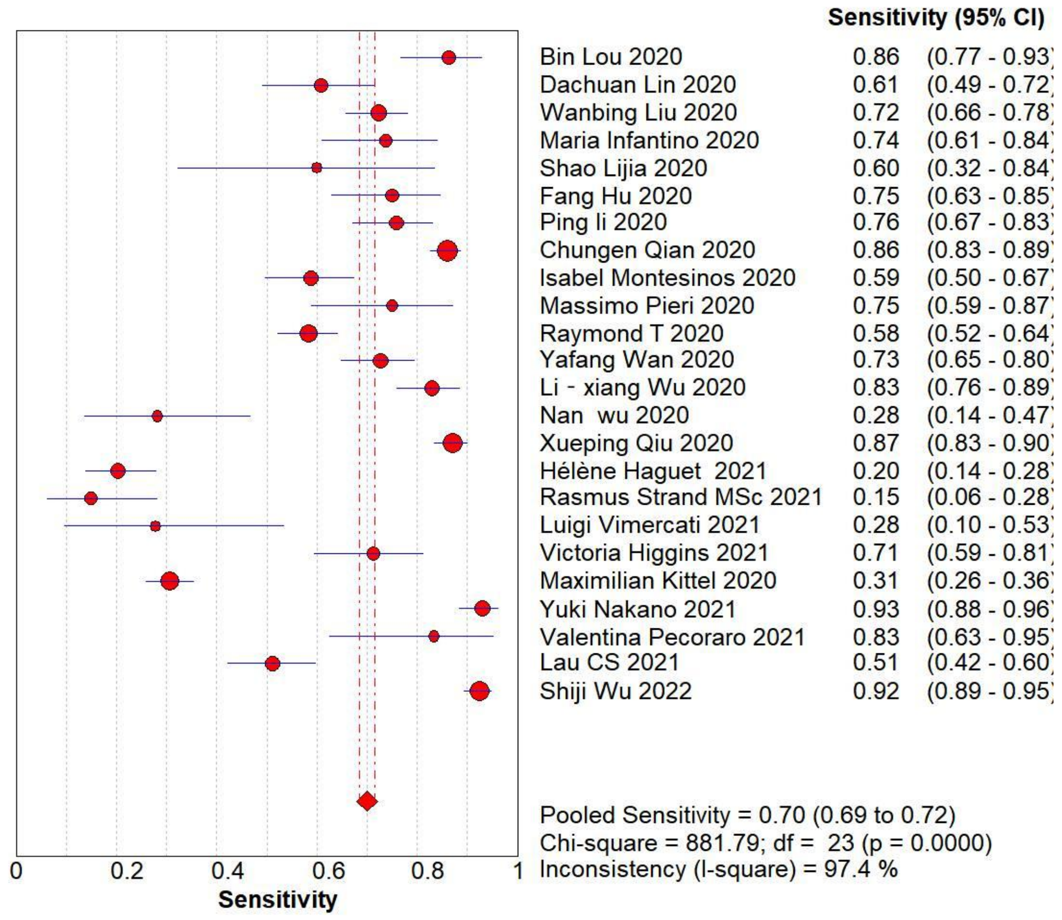

16.Supplementary figure S2 Meta-analytical estimates of sensitivity(with 95%)by serological test method and antibody class--Forest plot of CLIA IgG or IgM sensitivity

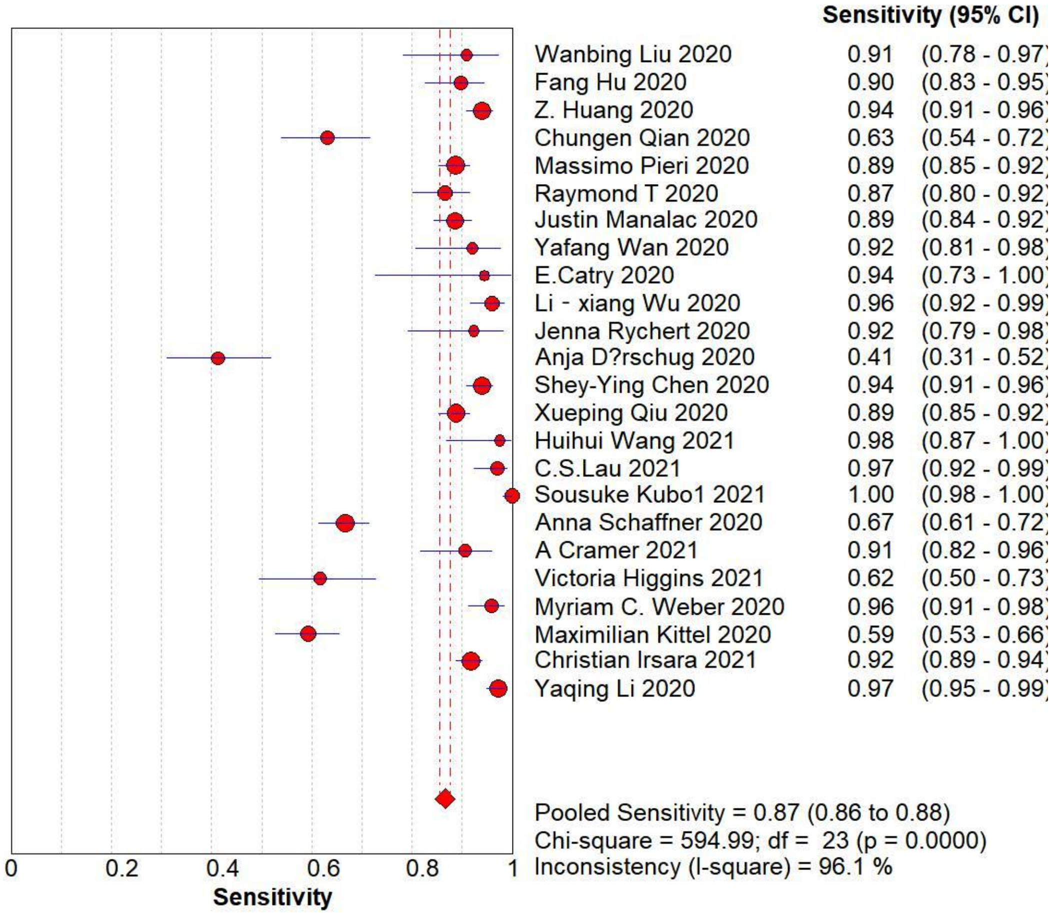

17.Supplementary figure S2 Meta-analytical estimates of sensitivity(with 95%)by serological test method and antibody class--Forest plot of LFIA IgG sensitivity

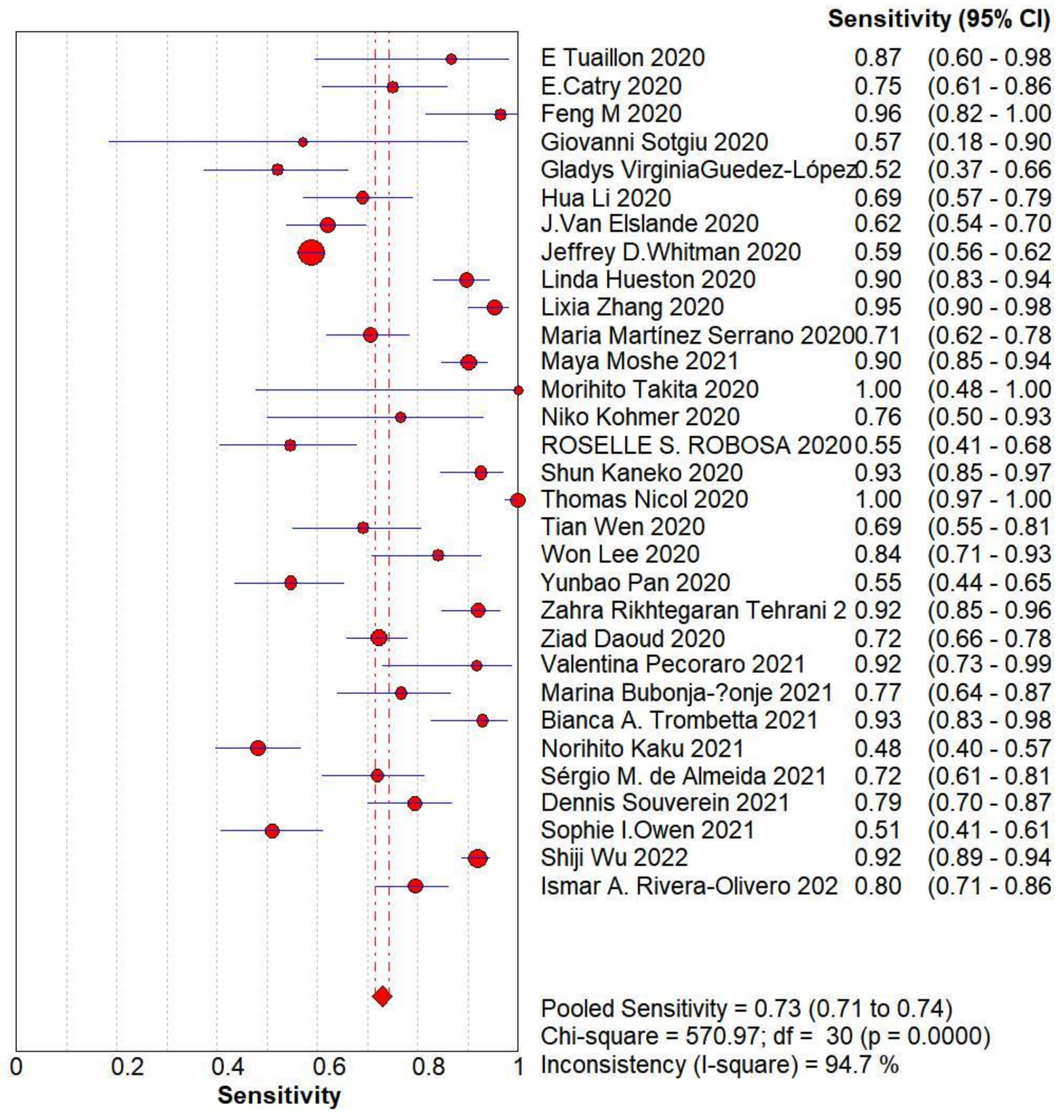

18.Supplementary figure S2 Meta-analytical estimates of sensitivity(with 95%)by serological test method and antibody class--Forest plot of LFIA IgM sensitivity

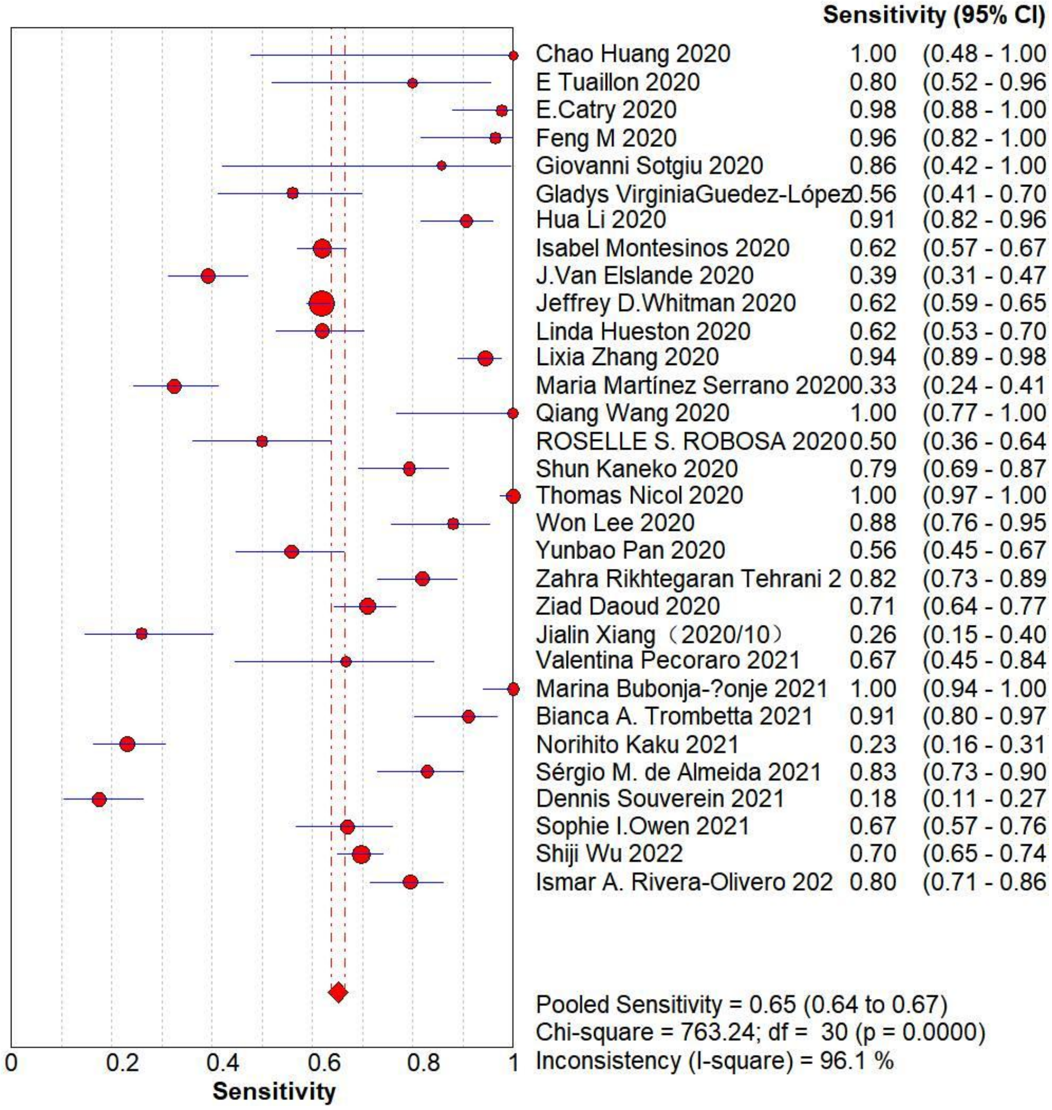

19.Supplementary figure S2 Meta-analytical estimates of sensitivity(with 95%)by serological test method and antibody class--Forest plot of LFIA IgG or IgM sensitivity

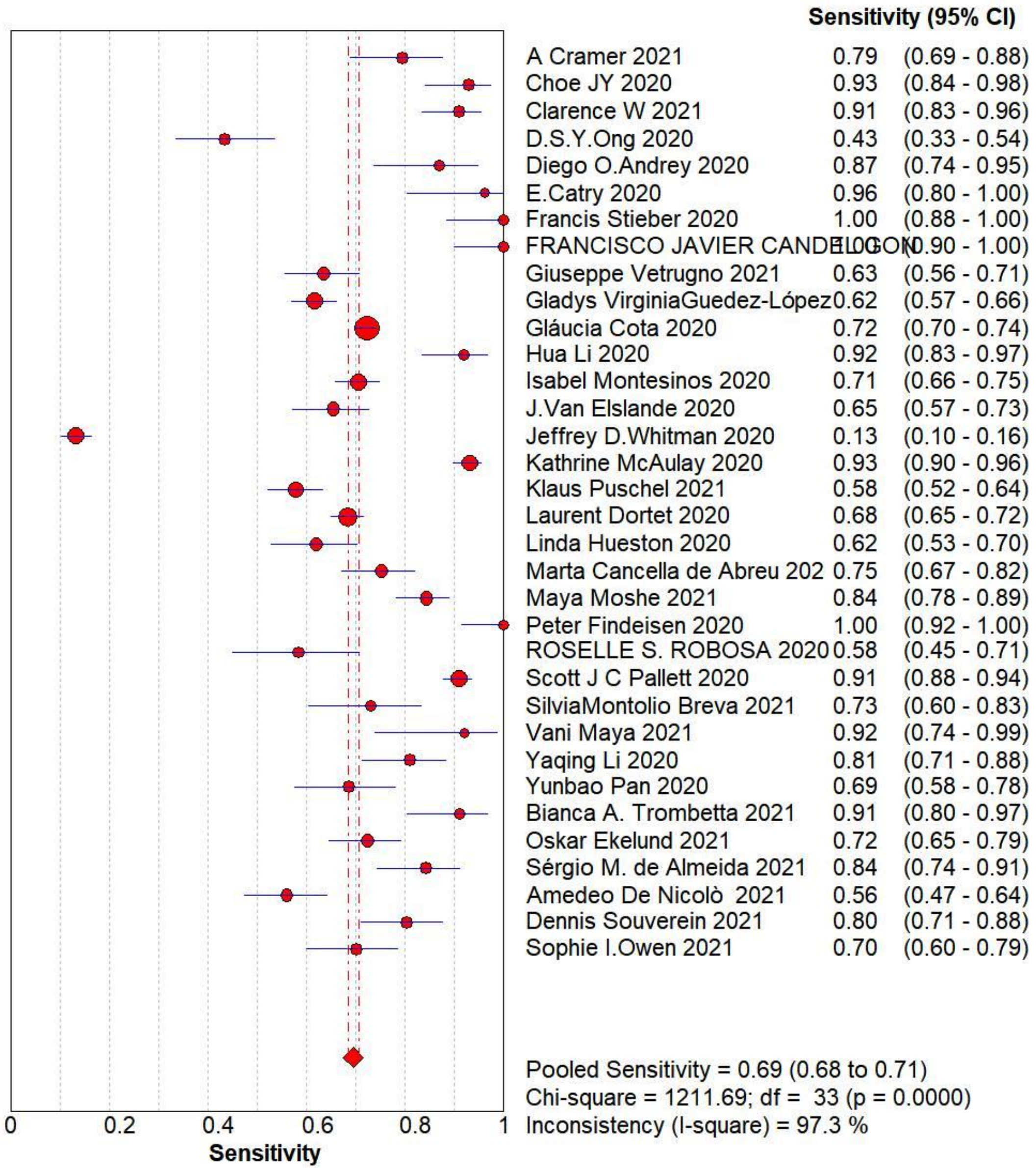

20.Supplementary figure S3 Meta-analytical estimates of specificity (with 95%)by serological test method and antibody class--ELISA IgG specificity

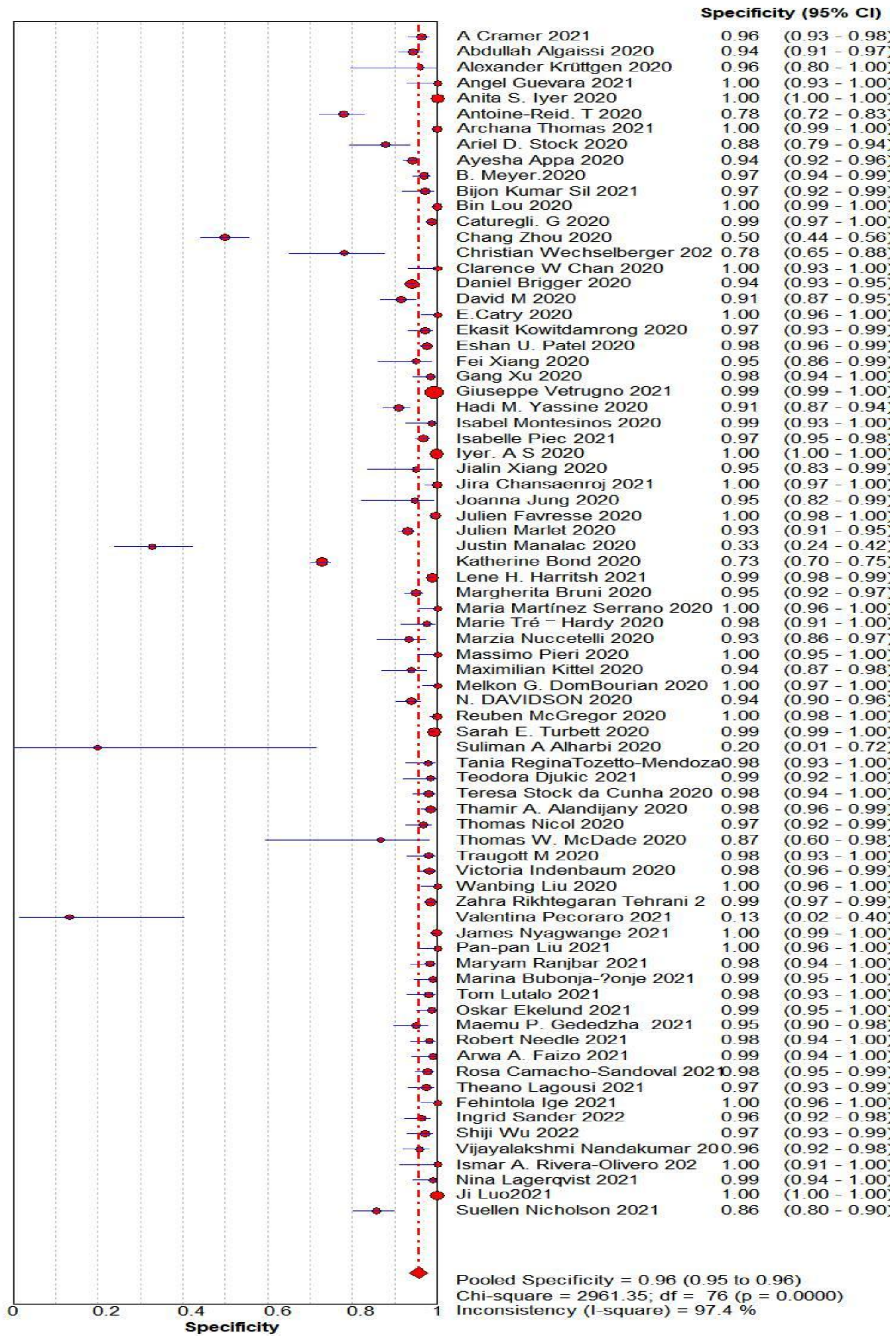

21.Supplementary figure S3 Meta-analytical estimates of specificity (with 95%)by serological test method and antibody class--ELISA IgM specificity

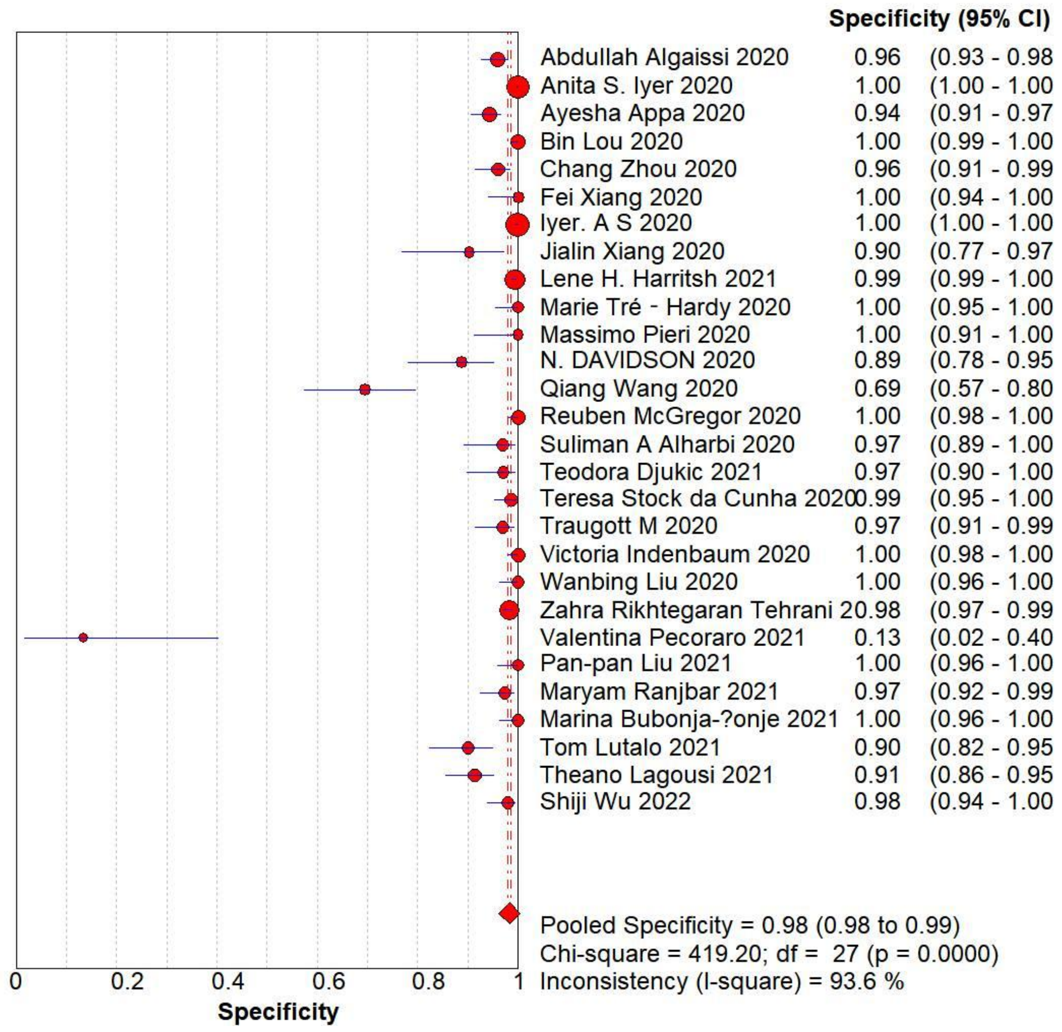

22.Supplementary figure S3 Meta-analytical estimates of specificity (with 95%)by serological test method and antibody class--ELISA IgG or IgM specificity

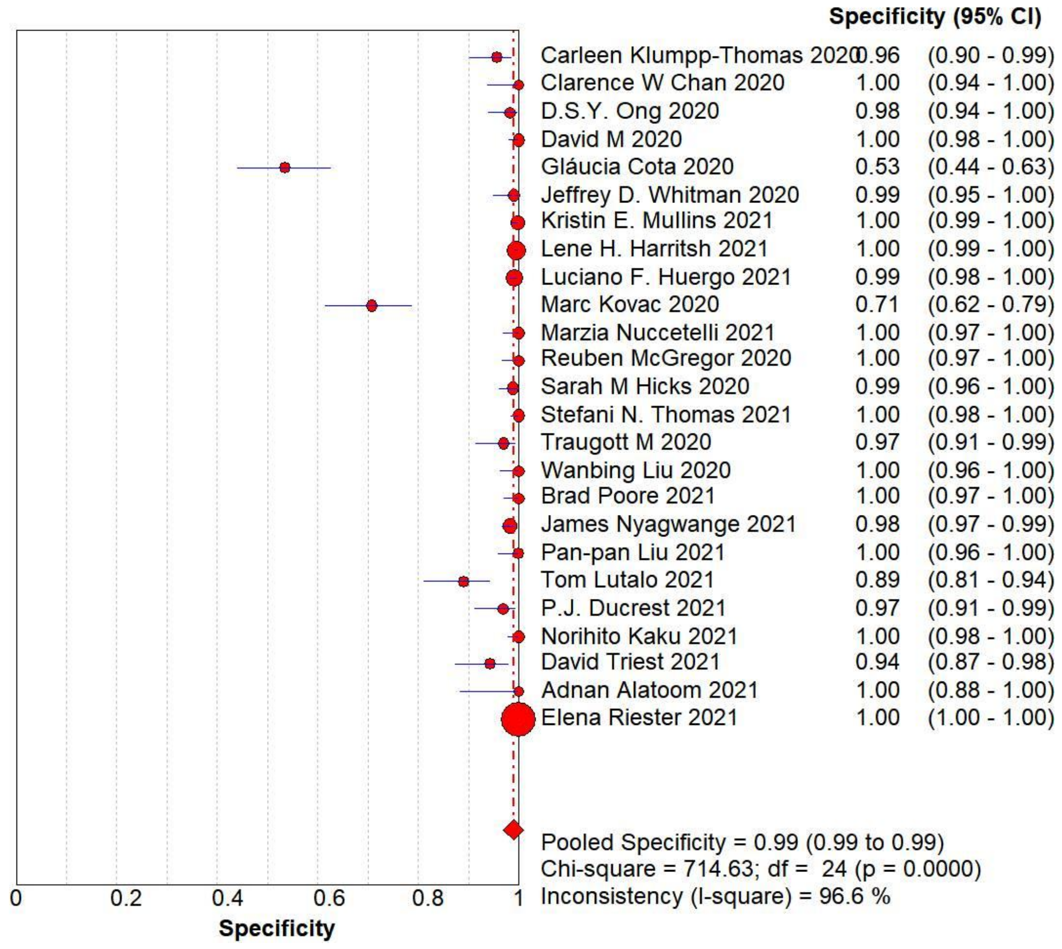

23.Supplementary figure S3 Meta-analytical estimates of specificity (with 95%)by serological test method and antibody class--CLIA IgG specificity

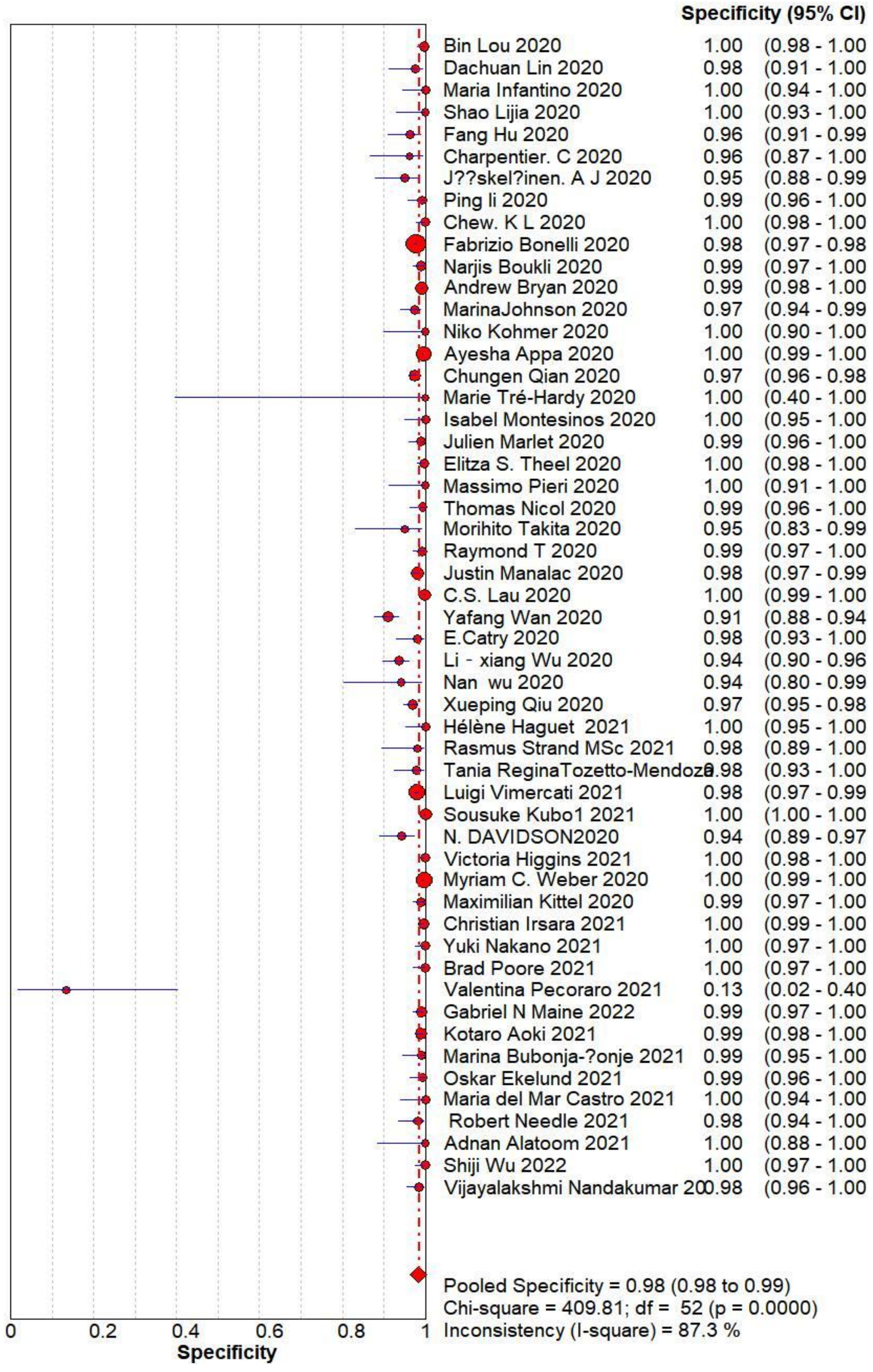

24.Supplementary figure S3 Meta-analytical estimates of specificity (with 95%)by serological test method and antibody class--CLIA IgM specificity

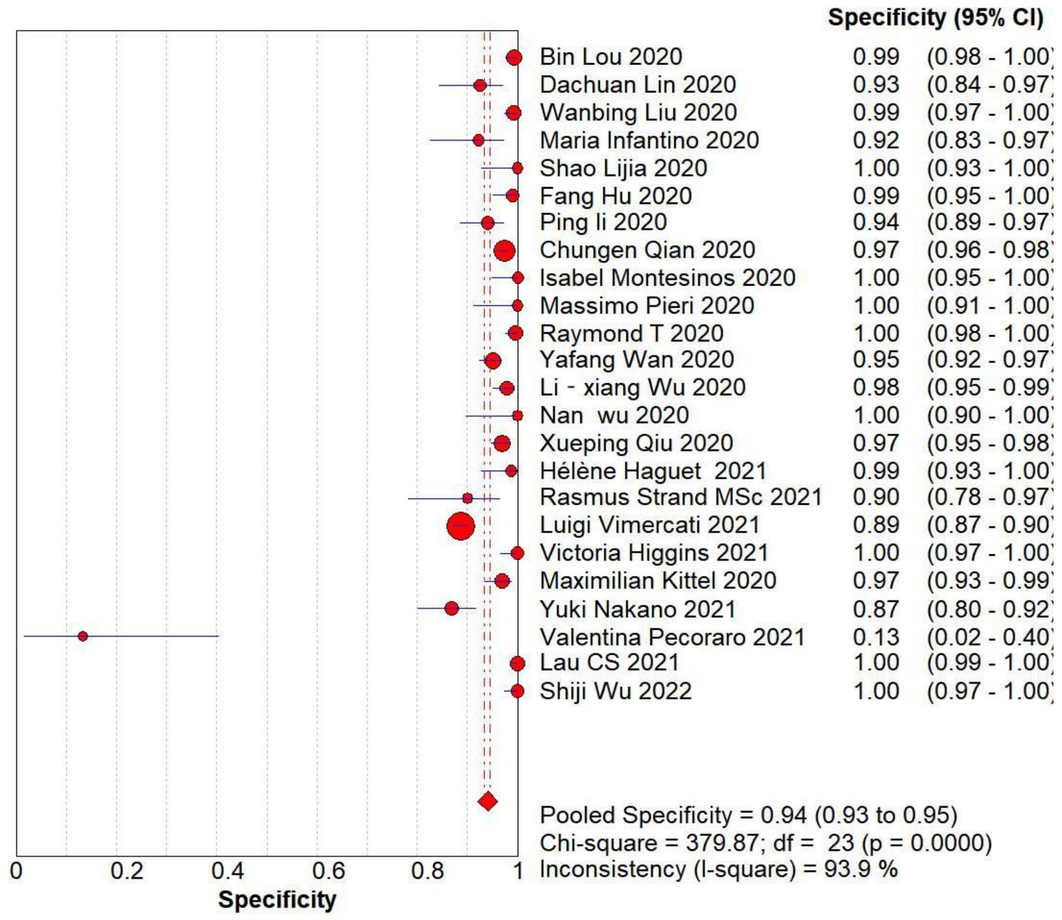

25.Supplementary figure S3 Meta-analytical estimates of specificity (with 95%)by serological test method and antibody class--CLIA IgG or IgM specificity

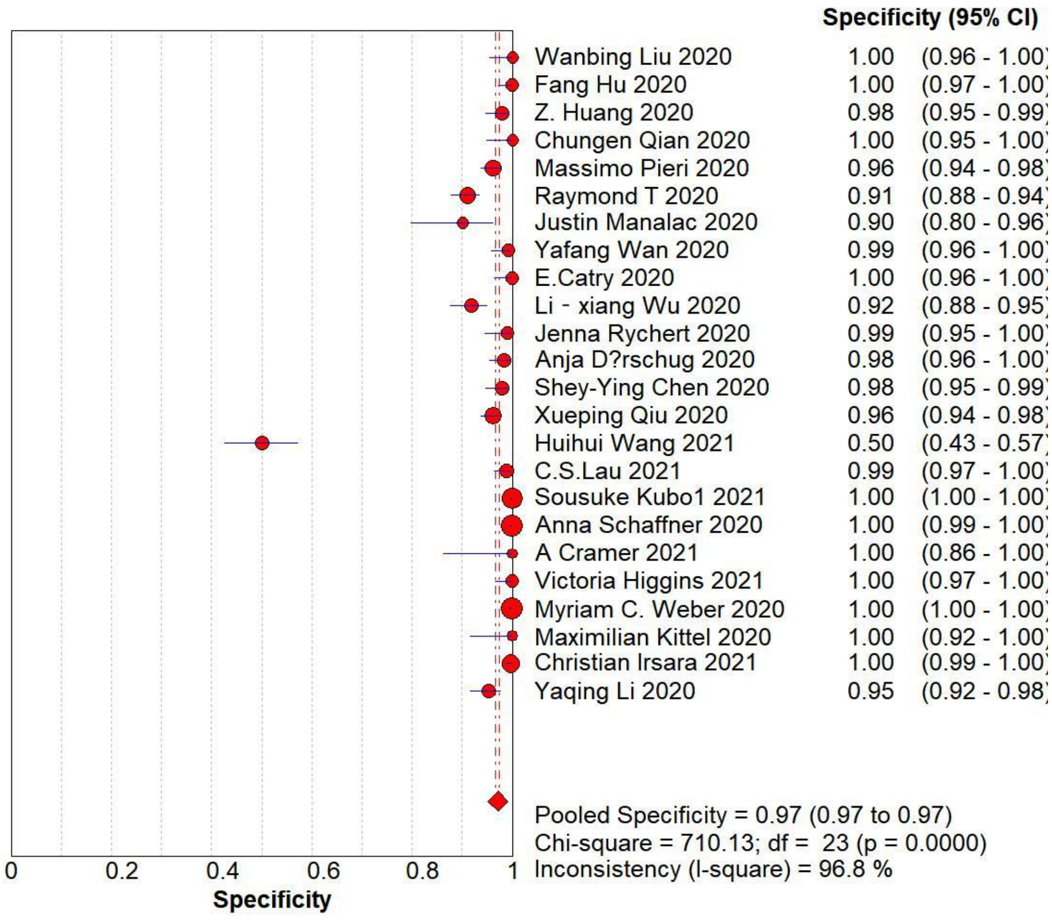

26.Supplementary figure S3 Meta-analytical estimates of specificity (with 95%)by serological test method and antibody class--LFIA IgG specificity

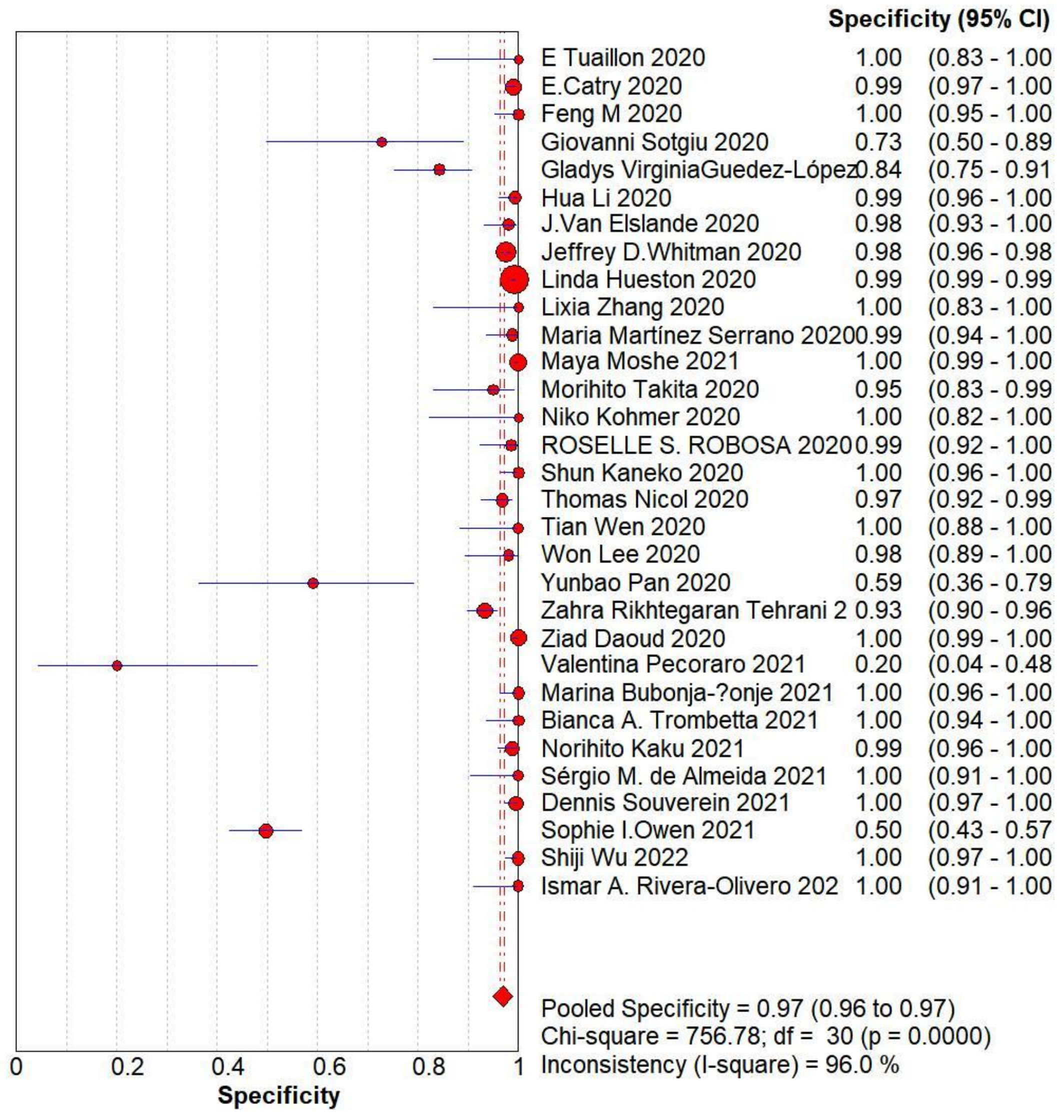

27.Supplementary figure S3 Meta-analytical estimates of specificity (with 95%)by serological test method and antibody class--LFIA IgM specificity

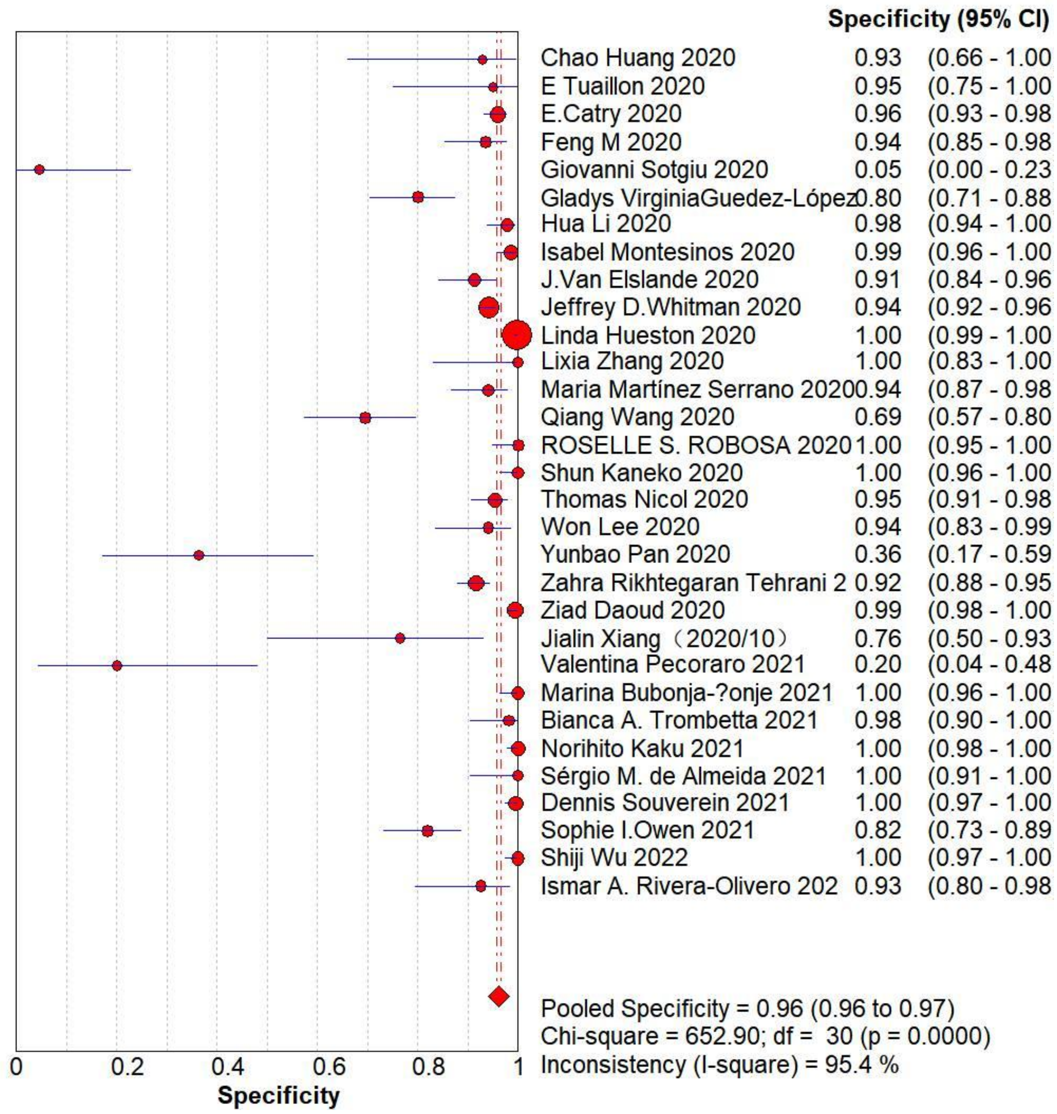

28.Supplementary figure S3 Meta-analytical estimates of specificity (with 95%)by serological test method and antibody class--LFIA IgG or IgM specificity

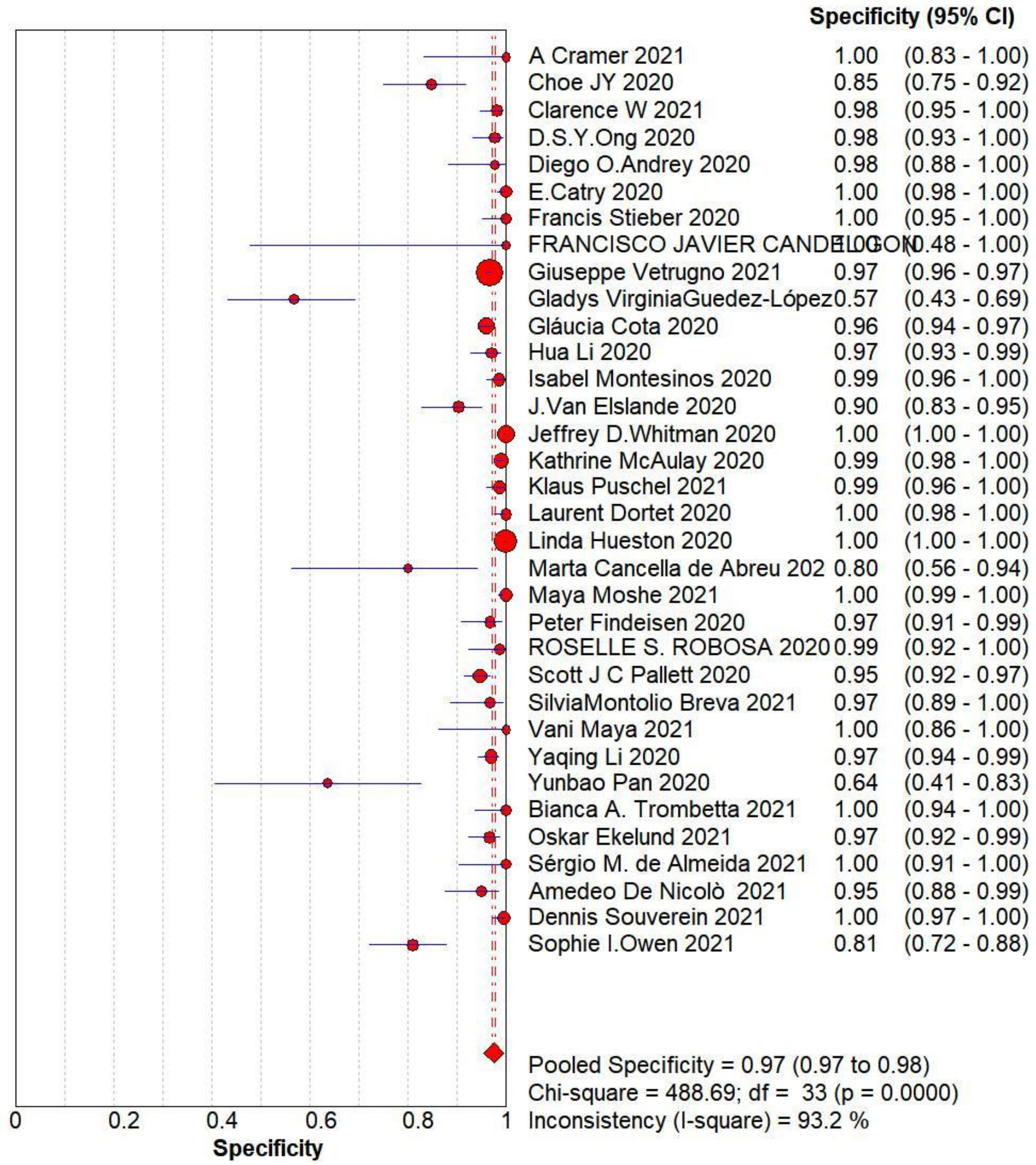

29.Supplementary S4 Sensitivity of ELISA Around the World

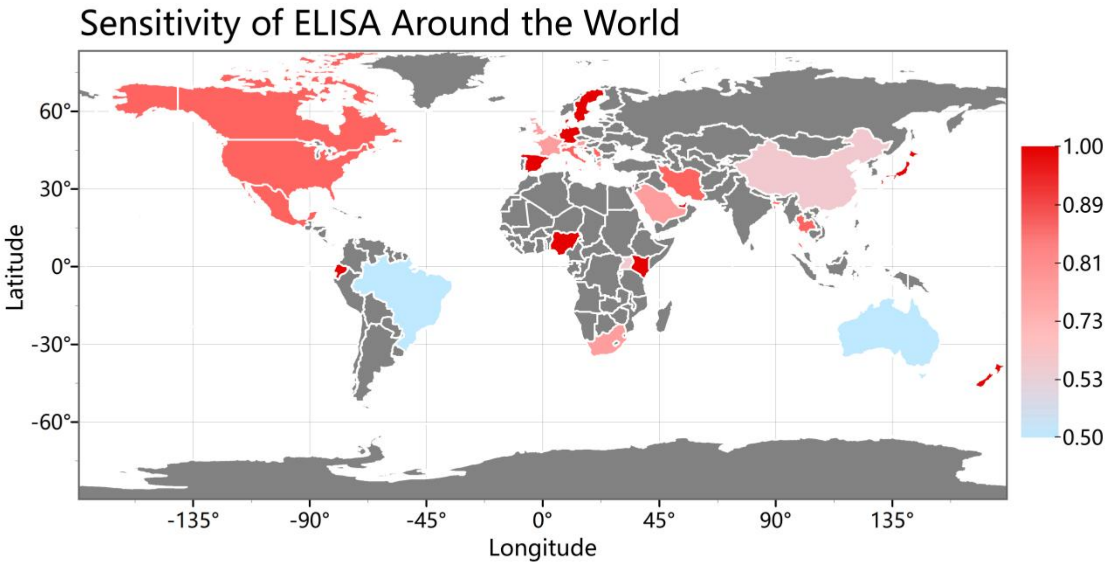

30.Supplementary S5 Sensitivity of CLIA Around the World

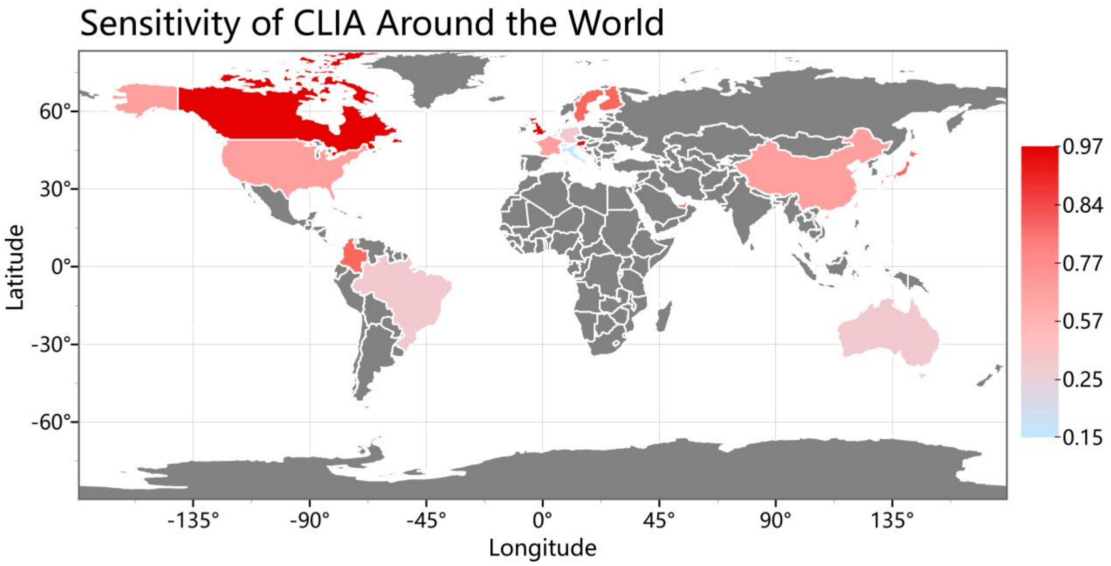

31.Supplementary S6 Sensitivity of LFIA Around the World

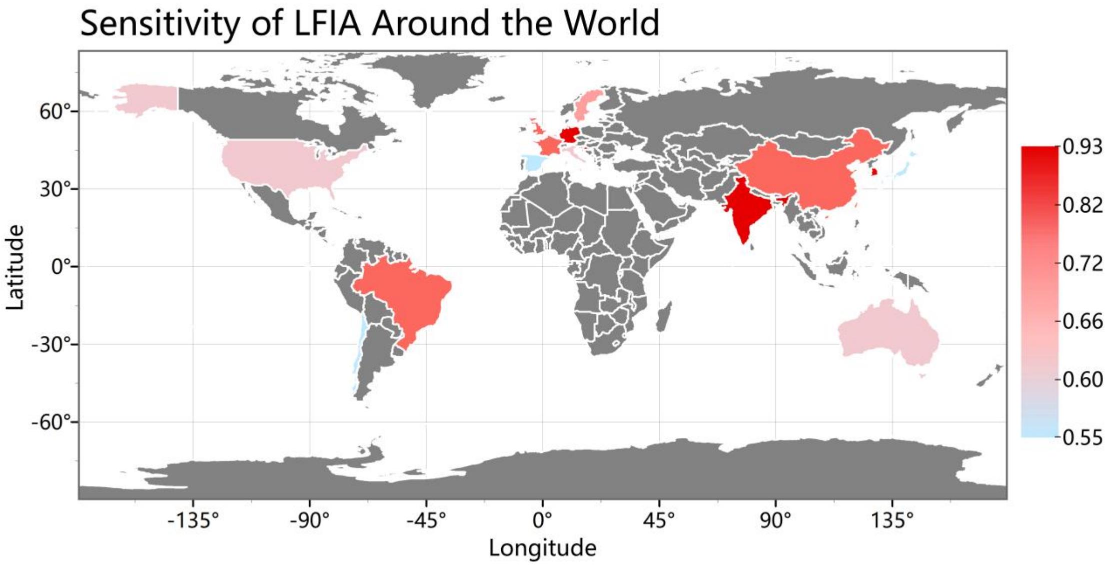

32.Supplementary S7 Specificity of ELISA Around the World

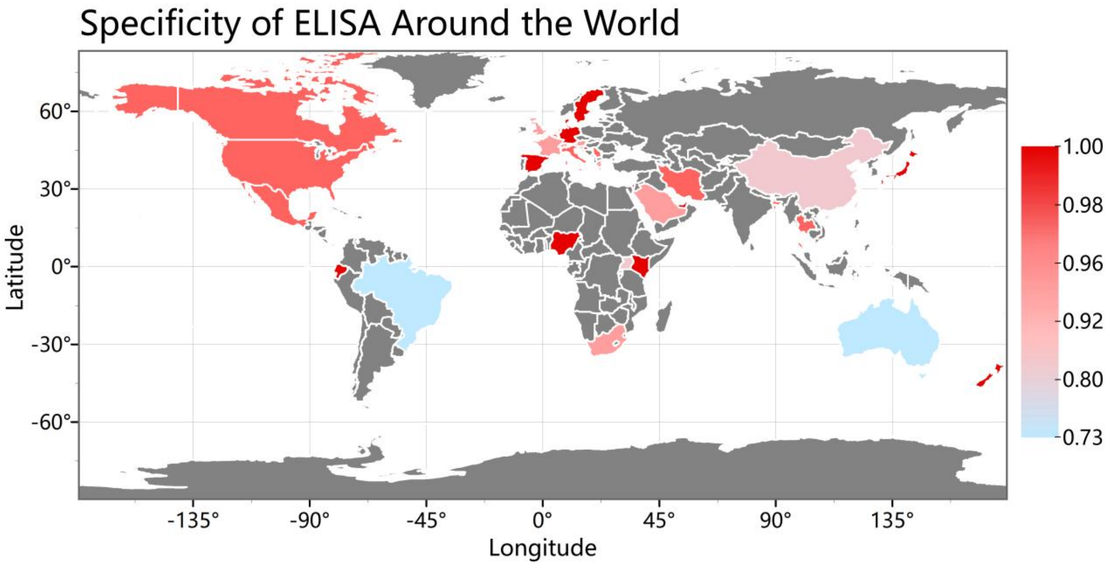

33.Supplementary S8 Specificity of CLIA Around the World

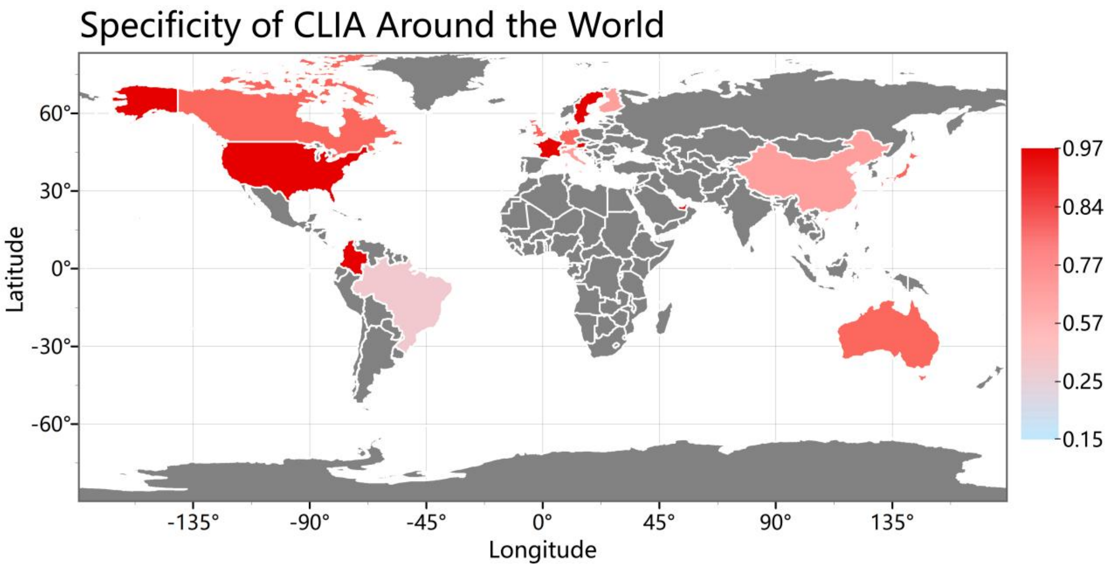

34.Supplementary S9 Specificity of LFIA Around the World

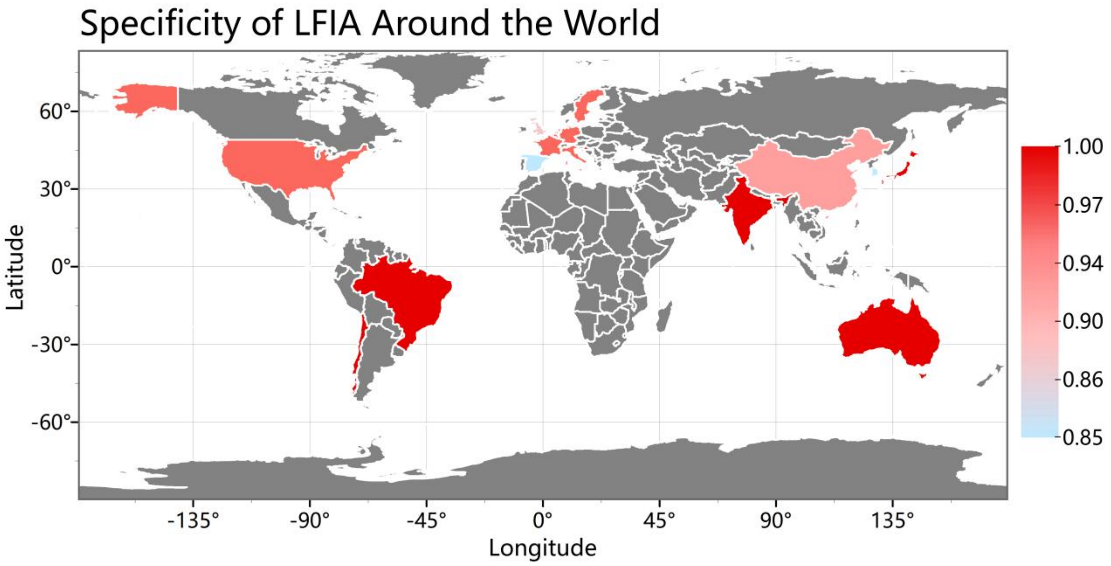

35.Supplementary table S1

author (years)	patient selection	index test(antibody test)	reference standard	flow and timing
A Cramer(2021/2)	high	unclear	low	unclear
Abdullah Algaissi (2020/10)	high	unclear	low	low
Alexander Kr¨uttgen (2020/9/19)	unclear	high	low	low
Andrew Bryan, (2020/7/23)	unclear	unclear	low	low
Angel Guevara(2021/2/25)	unclear	high	low	low
Anita S. Iyer (2020/10/8)	unclear	high	low	low
Anja Dörschug (2020/9/25)	unclear	high	low	low
Anna Schaffner(2020/12)	unclear	unclear	low	low
Antoine-Reid, T, (2020/8/4)	unclear	unclear	low	low
Archana Thomas(2021/1)	high	unclear	low	low
Ariel D. Stock (2020/8)	unclear	unclear	low	low
Ayesha Appa, (2020/8/17)	low	high	low	low
B. Meyer (2020/6)	high	unclear	low	low
Bijon Kumar Sil(2021/2)	high	unclear	low	low
Bin Lou, (2020/8/27)	unclear	high	low	low
C S Lau (2020/9/8)	unclear	unclear	low	low
C.S.Lau(2021/1/19)	unclear	unclear	low	low
Carleen Klumpp-Thomas, (2020/5/25)	unclear	high	low	low
Caturegli, G, (2020/7/6)	unclear	high	low	low
Chang Zhou (2020/12/17)	unclear	high	low	unclear
Chao Huang, (2020/5/18)	unclear	high	low	low

Charpentier, C(2020/1/1)	unclear	high	low	low
Chew, K L, （2020/6/9）	unclear	high	low	low
Choe, J Y（2020/10/13）	unclear	high	low	low
Christian Irsara(2021/1)	high	unclear	low	unclear
Christian Wechselberger（2020/8）	high	unclear	low	low
Chungen Qian（2020/7）	high	unclear	low	low
Clarence W Chan（2020/11/2）	unclear	high	low	unclear
D.S.Y. Ong（2020/6）	unclear	unclear	low	low
Dachuan Lin,（2020/7/17）	unclear	high	low	low
Daniel Brigger（2020/9/30）	unclear	high	low	low
David M Manthei（2020/10/9）	unclear	high	low	unclear
Diego O. Andrey, （2020/7/21）	unclear	high	low	low
E Tuaillon（2020/6）	unclear	unclear	low	low
E.Catry（2020/12/24）	unclear	high	low	unclear
Ekasit Kowitdamrong, （2020/10/9）	unclear	unclear	low	low
Zeno Bisoffi(2020/9)	high	unclear	low	low
Eshan U. Patel(2020/12/02)	high	unclear	low	unclear
Fabrizio Bonelli,（2020/6/24）	unclear	high	low	low
Fang Hu（2020/9/24）	unclear	high	low	low
Fei Xiang（2020/10/15）	unclear	high	low	unclear
Feng, M（2020/1/1）	unclear	high	low	low
Francis Stieber（2020/10）	high	unclear	low	low
FRANCISCO JAVIER CANDEL GONZÁLEZ, （2020/6/4）	unclear	high	low	low
Gang Xu（2020/8）	high	unclear	low	low
Giovanni Sotgiu	unclear	high	low	low
Giuseppe Vetrugno(2021/3)	unclear	high	low	unclear

Gladys Virginia Guedez-López (2020/8/17)	unclear	high	low	low
Gláucia Cota, (2020/10/9)	unclear	high	low	low
Hadi M. Yassine (2020/10)	high	unclear	low	low
Hélène Haguet (2021/2/18)	unclear	high	low	low
Hua Li, (2020 Jun 5)	unclear	high	low	low
Huihui Wang(2021/1/23)	unclear	high	low	low
Isabel Montesinos(2020/4)	high	unclear	low	low
Isabelle Piec(2021/2)	high	unclear	low	unclear
Iyer, A S, (2020/10/8)	unclear	high	low	low
J. Van Elslande(2020/8)	high	unclear	low	unclear
Jääskeläinen, A J (2020/8/1)	unclear	high	low	low
Jeffrey D. Whitman (2020/8)	high	high	low	low
Jenna Rychert (2020/10/16)	unclear	high	low	low
Jialin Xiang (2020/10)	unclear	unclear	low	low
Jira Chansaenroj(2021/3)	high	unclear	low	low
Joanna Jung, (2020/9/19)	unclear	unclear	low	low
Julien Favresse (2020/11/17)	unclear	unclear	low	low
Julien Marlet (2020/9)	high	high	low	low
Justin Manalac (2020/07/02)	high	unclear	low	unclear
Katherine Bond(2020/6)	unclear	unclear	low	low
Kathrine McAulay(2020/08/02)	unclear	high	low	unclear
Klaus Puschel (2021/2/23)	unclear	high	low	low
Kristin E. Mullins(2021/1)	high	unclear	low	unclear
Laurent Dortet (2020/10/7)	unclear	high	low	low
Linda Hueston, (2020/8/27)	unclear	high	low	low
Li- Xia Zhang(2020/07/24)	high	high	low	unclear

Li - xiang Wu(2020/11/13)	unclear	high	low	low
Luciano F. Huergo (2021/01/20)	high	high	low	low
Luigi Vimercati (2021/01/27)	high	high	low	unclear
Marc Kovac (2020/8/14)	unclear	unclear	low	low
Margherita Bruni ， (2020/10/1)	unclear	high	low	low
Maria Infantino， (2020/4/24)	unclear	unclear	low	low
Maria Martínez Serrano (2020/6)	high	unclear	low	low
Marie Tré-Hardy (2020/5)	high	unclear	low	low
MarinaJohnson， (2020/8/18)	unclear	high	low	low
Marta Cancella de Abreu， (2020/7/30)	unclear	high	low	low
Marzia Nuccetelli 2020	high	unclear	low	low
Marzia Nuccetelli 2021	unclear	unclear	low	low
Massimo Pieri (2020/9)	high	high	low	low
Maximilian Kittel(2020/12)	high	unclear	low	low
Maya Moshe (2021/02/11)	high	high	low	unclear
Melkon G. DomBourian(2020/8/20)	high	high	low	low
Morihito Takita (2020/6)	high	high	low	unclear
Myriam C. Weber (2020/11)	high	unclear	low	unclear
N. DAVIDSON (2020/12)	high	unclear	low	low
Narjis Boukli， (2020/10/21)	low	high	low	low
Niko Kohmer， (2020/8/1)	unclear	high	low	low
Peter Findeisen(2020/11)	high	high	low	low
Qiang Wang(2020/5)	high	high	low	low
Rasmus Strand MSc(2021/3/15)	unclear	high	low	low
Raymond T(2020/12/01)	high	high	low	unclear
Reuben McGregor (2020/9/3)	unclear	unclear	low	low

ROSELLE S. ROBOSA(2020/9)	high	high	low	low
Sarah E. Turbett(2020/10)	high	unclear	low	low
Sarah M Hicks (2020/10/3)	unclear	unclear	low	low
Scott J C Pallett(2020/12/08)	low	high	low	unclear
Shao Lijia (2020/7/16)	unclear	unclear	low	low
Shey-Ying Chen (2020/10/1)	unclear	high	low	low
Shun Kaneko MD, PhD (2020/7/28)	unclear	high	low	low
SilviaMontolio Breva (2021/1/20)	unclear	high	low	unclear
Sousuke Kubo1	high	unclear	low	unclear
Stefani N. Thomas (2021/2)	high	unclear	low	low
Suliman A Alharbi, (2020/9/6)	high	high	low	low
Tania ReginaTozetto-Mendoza (2021/1/23)	unclear	high	low	low
Teodora Djukic(2021/2)	high	unclear	low	unclear
Teresa Stock da Cunha , (2020/7/16)	unclear	high	low	unclear
Thamir A. Alandijany (2020/9/28)	unclear	high	low	low
Thomas Nicol(2020/8)	high	high	low	low
Thomas W. McDade(2020/8)	unclear	high	low	low
Tian Wen(2020/05/29)	high	high	low	unclear
Traugott M(2020/5)	high	unclear	low	low
Vani Maya(2021/3/4)	unclear	high	low	low
Victoria Higgins (2021/1)	high	unclear	low	low
Victoria Indenbaum (2020/11)	high	unclear	low	low
Wanbing Liu,(2020/5/26)	unclear	high	low	low
Won Lee (2020/12/4)	unclear	high	low	low
Xueping Qiu (2020/8)	unclear	unclear	low	low
Yafang Wan (2020/11/12)	unclear	unclear	low	low

Yaqing Li, MD, （2020/9/23）	unclear	high	low	low
Yuki Nakano(2021/2)	high	unclear	low	unclear
Yunbao Pan（2020/4）	high	high	low	low
Z. Huang, （2020/8/7）	unclear	high	low	low
Zahra Rikhtegaran Tehrani（2020/11）	high	unclear	low	low
Ziad Daoud（2020/10）	high	unclear	low	low
Clarence W Chan(2020/11/2)	high	high	low	low
Marie Tré-Hardy(2020/7/27)	high	unclear	low	low
Wanbing Liu(2020/8/1)	high	high	low	low
Brad Poore（2021/6/9）	H	unclear	low	low
Valentina Pecoraro（2021/4/10）	unclear	unclear	unclear	low
Gabriel N Maine（2022/2/16）	high	unclear	low	unclear
Kotaro Aoki(2021/5/10)	high	unclear	low	low
James Nyagwange(2021/12/22)	unclear	unclear	low	unclear
Pan-pan Liu(2021/3/22)	high	unclear	low	unclear
Maryam Ranjbar(2021/12/19)	high	unclear	low	low
Marina Bubonja-Šonje(2021/4/20)	high	high	low	unclear
Bianca A. Trombetta(2021/5/25)	high	unclear	unclear	unclear
Tom Lutalo(2021/9/13)	high	high	low	unclear
Oskar Ekelund(2021/5/29)	high	high	low	unclear
P.J. Ducrest(2021/4/30)	high	unclear	low	unclear
Lau CS(2021/4/21)	high	unclear	low	low
Norihito Kaku(2021/9/1)	high	unclear	low	unclear
David Triest(2021/6/8)	high	unclear	low	unclear
Maemu P. Gededzha (2021/6/23)	high	unclear	low	unclear
Maria del Mar Castro(2021/8/10)	unclear	low	low	low

Sérgio M. de Almeida(2021/4/15)	unclear	unclear	low	unclear
Robert Needle(2021/4/16)	unclear	unclear	low	unclear
Arwa A. Faizo (2021/4/30)	high	unclear	low	unclear
Amedeo De Nicolò (2021/5/10)	high	unclear	low	low
Dennis Souverein(2021/12/22)	high	low	low	unclear
Rosa Camacho-Sandoval (2021/8/17)	high	unclear	low	unclear
Theano Lagousi(2021/10/20)	high	unclear	low	low
Adnan Alatoom(2021/4/18)	high	unclear	low	unclear
Fehintola Ige(2021/10/6)	unclear	unclear	low	low
Ingrid Sander(2022/1/30)	high	unclear	low	low
Sophie I.Owen(2021/12/11)	high	unclear	low	low
Shiji Wu(2022/8/8)	unclear	unclear	low	low
Vijayalakshmi Nandakumar(2021/6/28)	high	unclear	low	low
Elena Riester(2021/8/9)	high	unclear	low	unclear
Ismar A. Rivera-Olivero(2022/2/18)	unclear	unclear	low	low
Nina Lagerqvist(2021/4/7)	high	unclear	low	low
Ji Luo(2022/3/19)	high	unclear	low	low
Suellen Nicholson(2021/5/10)	unclear	unclear	low	unclear

36. supplementary table S2

	Author	PMID	TEST METHOD
Evaluation of Serological Tests for SARS-CoV-2: Implications for Serology Testing in a Low-Prevalence Setting	Katherine Bond	32761124	ELISA
Comparative analysis of three laboratory based serological assays for SARS-CoV-2 in an Australian cohort	N. DAVIDSON	33070955	ELISA， CLIA
A Dual-Antigen Enzyme-Linked Immunosorbent Assay Allows the Assessment of Severe Acute Respiratory Syndrome Coronavirus 2 Antibody Seroprevalence in a Low-Transmission Setting	Sarah M Hicks	33009908	ELISA
Performance evaluation of serological assays to determine the immunoglobulin status in SARS-CoV-2 infected patients	Christian Wechselberger	32810840	ELISA
Performance of SARS-CoV-2 antibody assays in different stages of the infection: Comparison of commercial ELISA and rapid tests	Traugott M	32473021	ELISA
Development and performance evaluation of a rapid in-house ELISA for retrospective serosurveillance of SARS-CoV-2	Bijon Kumar Sil	33529223	ELISA
Analytical and clinical evaluation of four commercial SARS-CoV-2 serological immunoassays in hospitalized patients and ambulatory individuals	E.Catry	33359614	ELISA， CLIA， LFIA
Evaluation of two automated and three rapid lateral flow immunoassays for the detection of anti-SARS-CoV-2 antibodies	Isabel Montesinos	32403010	ELISA， CLIA， LFIA
Performance of SARS-CoV-2 serology tests:Are they good enough?	Isabelle Piec	33596236	ELISA

An original multiplex method to assess five different SARS-CoV-2 antibodies	Julien Favresse	33554567	ELISA
Analytical and clinical validation of an ELISA for specific SARS-CoV-2 IgG, IgA, and IgM antibodies	Marie Tré-Hardy	32667733	ELISA
Diagnostic performance of commercially available COVID-19 serology tests in Brazil	Gláucia Cota	33039612	ELISA，LFIA
Nucleoprotein-based ELISA for detection of SARS-COV-2 IgG antibodies: Could an old assay be suitable for serodiagnosis of the new coronavirus?	Tania ReginaTozetto-Mendoza	33453299	ELISA，CLIA
Serology characteristics of SARS-CoV-2 infection after exposure and post-symptom onset	Bin Lou	32430429	ELISA，CLIA
Evaluation of serum IgM and IgG antibodies in COVID-19 patients by enzyme linked immunosorbent assay	Chang Zhou	33331654	ELISA
Antibody Detection and Dynamic Characteristics in Patients with COVID-19	Fei Xiang	32306047	ELISA
Comparative analysis of the main haematological indexes and RNA detection for the diagnosis of SARS-CoV-2 infection	Jialin Xiang	33081702	ELISA
A method to prevent SARS-CoV-2 IgM false positives in gold immunochromatography and enzyme-linked immunosorbent assays	Qiang Wang	32277023	ELISA，LFIA
Evaluation of Nucleocapsid and Spike Protein-Based Enzyme-Linked Immunosorbent Assays for Detecting Antibodies against SARS-CoV-2	Wanbing Liu	32229605	ELISA
A Low-Cost SARS-CoV-2 rRBD ELISA to Detect Serostatus in Ecuadorian Population with COVID-19	Angel Guevara	33630751	ELISA

Clinical performance of four immunoassays for antibodies to SARS-CoV-2, including a prospective analysis for the diagnosis of COVID-19 in a real-life routine care setting	Julien Marlet	32927357	ELISA，CLIA
Assessment of SARS-CoV-2 serological tests for the diagnosis of COVID-19 through the evaluation of three immunoassays: Two automated immunoassays (Euroimmun and Abbott) and one rapid lateral flow immunoassay (NG Biotech)	Thomas Nicol	32593133	ELISA，CLIA，LFIA
Magnetic Bead-Based Immunoassay Allows Rapid, Inexpensive, and Quantitative Detection of Human SARS-CoV- 2 Antibodies	Luciano F. Huergo	33496577	ELISA
Clinical evaluation of commercial automated SARS-CoV-2 immunoassays	Maximilian Kittel	33310108	ELISA，CLIA
Testing IgG antibodies against the RBD of SARS-CoV-2 is sufficient and necessary for COVID-19 diagnosis	Victoria Indenbaum	33227020	ELISA
Comparison of four new commercial serologic assays for determination of SARS-CoV-2 IgG	Alexander Krüttgen	32416599	ELISA
COVID-19 Seroprevalence among Healthcare Workers of a Large COVID-19 Hospital in Rome Reveals Strengths and Limits of Two Different Serological Tests	Giuseppe Vetrugno	33800721	ELISA，LFIA
Persistence of Anti-SARS-CoV-2 Antibodies in Non-Hospitalized COVID-19 Convalescent Health Care Workers	Margherita Bruni	33019628	ELISA
Combined anti-SARS-CoV-2 IgA, IgG, and IgM Detection as a Better Strategy to Prevent Second Infection Spreading Waves	Marzia Nuccetelli	32945214	ELISA
Evaluation of a new simultaneous anti-SARS-CoV-2 IgA, IgM and IgG screening automated assay based on native inactivated virus	Marzia Nuccetelli	33412393	ELISA
SARS-CoV-2 infection serology validation of different methods: Usefulness of IgA in the early phase of infection	Massimo Pieri	33002475	ELISA，CLIA

Comparison of diagnostic accuracies of rapid serological tests and ELISA to molecular diagnostics in patients with suspected coronavirus disease 2019 presenting to the hospital	D.S.Y. Ong	32502646	ELISA，LFIA
Collaborative networks enable the rapid establishment of serological assays for SARS-CoV-2 during nationwide lockdown in New Zealand	Reuben McGregor	32953275	ELISA
Performance evaluation of five ELISA kits for detecting anti-SARS-COV-2 IgG antibodies	Hadi M. Yassine	33127504	ELISA
SARS-CoV-2 S1 and N-based serological assays reveal rapid seroconversion and induction of specific antibody response in COVID-19 patients	Abdullah Algaissi	33024213	ELISA
Enzyme-Linked Immunosorbent Assay for the Detection of Severe Acute Respiratory Syndrome Coronavirus 2 (SARS-CoV-2) IgM/IgA and IgG Antibodies Among Healthcare Workers	Suliman A Alharbi	33047077	ELISA
Development and Optimization of In-house ELISA for Detection of Human IgG Antibody to SARS-CoV-2 Full Length Spike Protein	Thamir A. Alandijany	32998438	ELISA
Expression, purification and immunological characterization of recombinant nucleocapsid protein fragment from SARS-CoV-2	Teodora Djukic	33582454	ELISA
Comparison of commercial lateral flow immunoassays and ELISA for SARS-CoV-2 antibody detection	Maria Martínez Serrano	32659710	ELISA，LFIA
The Spectrum of Clinical and Serological Features of COVID-19 in Urban Hemodialysis Patients	Teresa Stock da Cunha	32708750	ELISA
Validation of a commercially available SARS-CoV-2 serological immunoassay	B. Meyer	32603801	ELISA
Accuracy of serological testing for SARS-CoV-2 antibodies: First results of a large mixed-method evaluation study	Daniel Brigger	32997812	ELISA

EDTA-Anticoagulated Whole Blood for SARS-CoV-2 Antibody Testing by Electrochemiluminescence Immunoassay (ECLIA) and Enzyme-Linked Immunosorbent Assay (ELISA)	Marc Kovac	32823852	ELISA
Antibody responses to SARS-CoV-2 in patients with differing severities of coronavirus disease 2019	Ekasit Kowitdamrong	33035234	ELISA
Detection of SARS-CoV-2-specifc antibodies via rapid diagnostic immunoassays in COVID-19 patients	Jira Chansaenroj	33750394	ELISA
Analytical evaluation and critical appraisal of early commercial SARS-CoV-2 immunoassays for routine use in a diagnostic laboratory	A Cramer	33308026	ELISA，CLIA，LFIA
Dynamics and significance of the antibody response to SARS-CoV-2 infection	Anita S. Iyer	32743600	ELISA
Cross-Comparison of a Chemiluminescent Platform and a Commercial Receptor Binding Domain–Based ELISA for Detecting SARS-CoV-2 IgG	Antoine-Reid, T	32750140	ELISA
Establishment of Monoclonal Antibody Standards for Quantitative Serological Diagnosis of SARS-CoV-2 in Low-Incidence Settings	Archana Thomas	33723513	ELISA
COVID-19 Infection Among Healthcare Workers: Serological Findings Supporting Routine Testing	Ariel D. Stock	32974370	ELISA
Sensitivity, Specificity and Predictive Values of Molecular and Serological Tests for COVID-19: A Longitudinal Study in Emergency Room	Zeno Bisoffi	32899333	ELISA
Standardization of enzyme-linked immunosorbent assays for serosurveys of the SARS-CoV-2 pandemic using clinical and at-home blood sampling	Carleen Klumpp-Thomas	32511472	ELISA

Clinical Validity of Serum Antibodies to SARS-CoV-2 : A Case-Control Study	Caturegli, G	32628534	ELISA
Analytical and Clinical Evaluation of the Automated Elecsys Anti–SARS-CoV-2 Antibody Assay on the Roche cobas e602 Analyzer	Clarence W Chan	32814955	ELISA
Differences in Performance Characteristics Among Four High-Throughput Assays for the Detection of Antibodies Against SARS-CoV-2 Using a Common Set of Patient Samples	David M	33033840	ELISA
Comparative performance of five commercially available serologic assays to detect 1 antibodies to SARS-CoV-2 and identify individuals with high neutralizing titers	Eshan U. Patel	33139419	ELISA
Evaluation of Orthogonal Testing Algorithm for Detection of SARS-CoV-2 IgG Antibodies	Gang Xu	32894753	ELISA
Persistence and decay of human antibody responses to the receptor binding domain of SARS-CoV-2 spike protein in COVID-19 patients	Iyer, A S	33033172	ELISA
Evaluation of SARS-CoV-2 serology assays reveals a range of test performance	Jeffrey D. Whitman	32855547	ELISA，LFIA
Clinical performance of a semi-quantitative assay for SARS-CoV2 IgG and SARS-CoV2 IgM antibodies	Joanna Jung	32956703	ELISA
Evaluation of Abbott anti-SARS-CoV-2 CMIA IgG and Euroimmun ELISA IgG/IgA assays in a clinical lab	Justin Manalac	32910980	ELISA，CLIA
Validation of COVID-19 serologic tests and large scale screening of asymptomatic healthcare workers	Kristin E. Mullins	33472036	ELISA
Analysis of COVID-19 convalescent plasma for SARS-CoV-2 IgG using two commercial immunoassays	Melkon G. DomBourian	32828791	ELISA

Evaluation of Three Commercial SARS-CoV-2 Serologic Assays and their Performance in Two-Test Algorithms	Sarah E. Turbett	33020186	ELISA
Initial determination of COVID-19 seroprevalence among outpatients and healthcare workers in Minnesota using a novel SARS-CoV-2 total antibody ELISA	Stefani N. Thomas	33539808	ELISA
High seroprevalence for SARS-CoV-2 among household members of essential workers detected using a dried blood spot assay	Thomas W. McDade	32797108	ELISA
Performance of nucleocapsid and spike_x005f_x005f_x0002_based SARS-CoV-2 serologic assays	Zahra Rikhtegaran Tehrani	33137138	ELISA，LFIA
Evaluations of the serological test in the diagnosis of 2019 novel coronavirus (SARS-CoV-2) infections during the COVID-19 outbreak	Dachuan Lin	32681308	CLIA
Clinical application of Chemiluminescence Microparticle Immunoassay for SARS-CoV-2 infection diagnosis	Wanbing Liu	32763810	CLIA
Diagnostic accuracy of an automated chemiluminescent immunoassay for anti-SARS-CoV-2 IgM and IgG antibodies: an Italian experience	Maria Infantino	32330291	CLIA
Serological chemiluminescence immunoassay for the diagnosis of SARS-CoV-2 infection	Shao Lijia	32671890	CLIA
Joint Detection of Serum IgM/IgG Antibody Is an Important Key to Clinical Diagnosis of SARS-CoV-2 Infection	Fang Hu	33014210	CLIA
Performance evaluation of two SARS-CoV-2 IgG/IgM rapid tests (Covid-Presto and NG-Test) and one IgG automated immunoassay (Abbott)	Charpentier, C	32919222	CLIA
Performance of six SARS-CoV-2 immunoassays in comparison with microneutralisation	Jääskeläinen, A J	32563180	CLIA

Clinical evaluation of serological IgG antibody response on the Abbott Architect for established SARS-CoV-2 infection	Chew, K L	32531475	CLIA
Clinical and Analytical Performance of an Automated Serological Test That Identifies S1/S2-Neutralizing IgG in COVID-19 Patients Semiquantitatively	Fabrizio Bonelli	32580948	CLIA
High Incidence of False-Positive Results in Patients with Acute Infections Other than COVID-19 by the Liaison SARS-CoV-2 Commercial Chemiluminescent Microparticle Immunoassay for Detection of IgG Anti-SARS-CoV-2 Antibodies	Narjis Boukli	32848041	CLIA
Performance Characteristics of the Abbott Architect SARS-CoV-2 IgG Assay and Seroprevalence in Boise, Idaho	Andrew Bryan	32381641	CLIA
Evaluation of a novel multiplexed assay for determining IgG levels and functional activity to SARS-CoV-2	MarinaJohnson	32769024	CLIA
Brief clinical evaluation of six high-throughput SARS-CoV-2 IgG antibody assays	Niko Kohmer	32505777	CLIA
Characteristics and roles of severe acute respiratory syndrome coronavirus 2-specific antibodies in patients with different severities of coronavirus 19	Z. Huang	32706417	CLIA
Universal PCR and antibody testing demonstrate little to no transmission of SARS-CoV-2 in a rural community	Ayesha Appa	32839781	CLIA
Development and multicenter performance evaluation of fully automated SARS-CoV-2 IgM and IgG immunoassays	Chungen Qian	32609640	CLIA
Dynamic changes of throat swabs RNA and serum antibodies for SARS-CoV-2 and their diagnostic performances in patients with COVID-19	Xueping Qiu	32787527	CLIA

Validation of a chemiluminescent assay for specific SARS-CoV-2 antibody	Marie Tré-Hardy	32447328	CLIA
Geographical Profiles of COVID-19 Outbreak in Tokyo: An Analysis of the Primary Care Clinic-Based Point-of-Care Antibody Testing	Morihito Takita	32674696	CLIA，LFIA
Longitudinal Monitoring of SARS-CoV-2 IgM and IgG Seropositivity to Detect COVID-19	Raymond T	33458749	CLIA
Performance of an automated chemiluminescence SARS-CoV-2 IG-G assay	C.S. Lau	32910979	CLIA
Performance verification of anti-SARS-CoV-2-specific antibody detection by using four chemiluminescence immunoassay systems	Yafang Wan	32961061	CLIA
Clinical significance of the serum IgM and IgG to SARS-CoV-2 in coronavirus disease-2019	Li-xiang Wu	33184940	CLIA
Evaluation of Three SARS CoV-2 IgG Antibody Assays and Correlation with Neutralizing Antibodies	Jenna Rychert	33064790	CLIA
Evaluation of the Xiamen AmonMed Biotechnology rapid diagnostic test COVID-19 IgM/IgG test kit (Colloidal gold)	Anja Dörschug	32979256	CLIA
Multicenter evaluation of two chemiluminescence and three lateral flow immunoassays for the diagnosis of COVID-19 and assessment of antibody dynamic responses to SARS-CoV-2 in Taiwan	Shey-Ying Chen	32940547	CLIA
Clinical performance of the Panbio assay for the detection of SARS-CoV-2 IgM and IgG in COVID-19 patients	Hélène Haguet	33599299	CLIA
Development of a SARS-CoV-2 rapid antibody detection kit and study on dynamic changes in antibodies in infected patients	Huihui Wang	33484220	CLIA

Performance of two rapid point of care SARS-COV-2 antibody assays against laboratory-based automated chemiluminescent immunoassays for SARS-COV-2 IG-G, IG-M and total antibodies	C.S.Lau	33501369	CLIA
Rapid diagnostic testing for SARS-CoV-2: Validation and comparison of three point-of-care antibody tests	Rasmus Strand MSc	33666238	CLIA
Large-scale IgM and IgG SARS-CoV-2 serological screening among healthcare workers with a low infection prevalence based on nasopharyngeal swab tests in an Italian university hospital: Perspectives for public health	Luigi Vimercati）	33508260	CLIA
Development of an Automated Chemiluminescence Assay System for Quantitative Measurement of Multiple Anti-SARS-CoV-2 Antibodies	Sousuke Kubo1	33519790	CLIA
Characterization of a Pan-Immunoglobulin Assay Quantifying Antibodies Directed against the Receptor Binding Domain of the SARS-CoV-2 S1-Subunit of the Spike Protein: A Population-Based Study	Anna Schaffner	33317059	CLIA
Anti-SARS-CoV-2 IgM improves clinical sensitivity early in disease course	Victoria Higgins	33476578	CLIA
Characteristics of three different chemiluminescence assays for testing for SARS-CoV-2 antibodies	Myriam C. Weber	33532006	CLIA
Evaluation of four commercial, fully automated SARS-CoV-2 antibody tests suggests a revision of the Siemens SARS-CoV-2 IgG assay	Christian Irsara	33554557	CLIA
Highlighted Prospects of an IgM/IgG Antibodies Test in Identifying Individuals With Asymptomatic Severe Acute Respiratory Syndrome Coronavirus 2 (SARS-CoV-2) Infection	Yaqing Li	32966561	CLIA，LFIA

Time course of the sensitivity and specifcity of anti- SARS- CoV- 2 IgM and IgG antibodies for symptomatic COVID- 19 in Japan	Yuki Nakano	33531605	CLIA
Rapid Detection of IgM Antibodies against the SARS-CoV-2 Virus via Colloidal Gold Nanoparticle-Based Lateral-Flow Assay	Chao Huang	32542208	LFIA
Clinical validation of an immunochromatographic SARS-Cov-2 IgM/IgG antibody assay with Japanese cohort	Shun Kaneko	32720704	LFIA
Evaluation of three immunochromatographic tests for rapid detection of antibodies against SARS-CoV-2	Guedez-López GV	32808111	LFIA
Diagnostic accuracy of Augurix COVID-19 IgG serology rapid test	Diego O.Andrey	32691863	LFIA
Development of a Sensitive Immunochromatographic Method Using Lanthanide Fluorescent Microsphere for Rapid Serodiagnosis of COVID-19	Feng M	32660240	LFIA
Clinical performance of different SARS-CoV-2 IgG antibody tests	Niko Kohmer	32510168	LFIA
SARS-CoV-2 IGM and IGG rapid serologic test for the diagnosis of COVID-19 in the emergency department	Marta Cancella de Abreu	32739490	LFIA
Utility of lateral flow tests in SARS-CoV-2 infection monitorization	FRANCISCO JAVIER CANDEL GONZÁLEZ	32492991	LFIA
A new and rapid approach for detecting COVID-19 based on S1 protein fragments	Hua Li	32508026	LFIA
Clinical evaluation of SARS-CoV-2 point-of-care antibody tests	ROSELLE S.ROBOSA	33039095	LFIA
Detection of SARS-CoV-2 antibodies using commercial assays and seroconversion patterns in hospitalized patients	E Tuaillon	32504735	LFIA
Diagnostic performance of seven rapid IgG/IgM antibody tests and the Euroimmun IgA/IgG ELISA in COVID-19 patients	J.Van Elslande	32473953	LFIA
SARS-CoV-2 specific serological pattern in healthcare workers of an Italian COVID-19 forefront hospital	Giovanni Sotgiu	32727446	LFIA

Serological immunochromatographic approach in diagnosis with SARS-CoV-2 infected COVID-19 patients	Yunbao Pan	32283141	LFIA
Diagnostic performance of immunochromatography assay for rapid detection of IgM and IgG in coronavirus disease 2019	Choe JY	32458479	LFIA
Development of a lateral flow immunoassay strip for rapid detection of IgG antibody against SARS-CoV-2 virus	Tian Wen	32568341	LFIA
Preliminary Analysis of B- and T- Cell Responses to SARS- CoV- 2	Lixia Zhang	32710269	LFIA
Retrospective clinical evaluation of 4 lateral flow assays for the detection of SARS-CoV-2 IgG	Kathrine McAulay	32947206	LFIA
Point-of-care serological assays for delayed SARS-CoV-2 case identification among health-care workers in the UK: a prospective multicentre cohort study	Scott J C Pallett	32717210	LFIA
Clinical Evaluation of a COVID-19 Antibody Lateral Flow Assay using Point of Care Samples	Won Lee	33300003	LFIA
Rapid Determination of SARS-CoV-2 antibodies using a bedside, point-of-Care, serological test	Laurent Dortet	32969769	LFIA
Evaluation of five immunoassays and one lateral flow immunochromatography for anti-SARS-CoV-2 antibodies detection	SilviaMontolio Breva	33558047	LFIA
Evaluation of diagnostic accuracy of developed rapid SARS-COV-2 IgG antibody test kit using novel diluent system	Vani Maya	33688556	LFIA
Clinical and serological profile of asymptomatic and non-severe symptomatic COVID-19 cases: Lessons from a longitudinal study in primary care in Latin America	Klaus Puschel	33199310	LFIA
SARS-CoV-2 lateral flow assays for possible use in national	Maya Moshe	33653694	LFIA

First performance report of QIAreach™ Anti-SARS-CoV-2 Total Test, an innovative nanoparticle fluorescence digital detection platform	Francis Stieber	33160178	LFIA
Higher Sensitivity Provided by the Combination of Two Lateral Flow Immunoassay Tests for the Detection of COVID-19 Immunoglobulins	Ziad Daoud	33194776	LFIA
Clinical performance evaluation of a SARS-CoV-2 Rapid Antibody Test for determining past exposure to SARS-CoV-2	Peter Findeisen	33227517	LFIA
Evaluation of the Truvian Easy Check COVID-19 IgM/IgG Lateral Flow Device for Rapid Anti-SARS-CoV-2 Antibody Detection	Clarence W Chan	33135049	LFIA
A comparison of SARS-CoV-2 nucleocapsid and spike antibody detection using three commercially available automated immunoassays	Brad Poore	34118242	ELISA,CLIA
Accuracy of the serological detection of IgG and IgM to SARS-Cov-2: a prospective, cross-sectional study	Valentina Pecoraro	33839972	ELISA,CLIA，LFIA
Comparative performance of WANTAI ELISA for total immunoglobulin to receptor binding protein and an ELISA for IgG to spike protein in detecting SARS-CoV-2 antibodies in Kenyan populations	James Nyagwange	34973474	ELISA
Development of a Nucleocapsid Protein-Based ELISA for Detection of Human IgM and IgG Antibodies to SARS-CoV-2	Pan-pan Liu	33869946	ELISA
Development of a recombinant nucleocapsid protein-based ELISA for the detection of IgM and IgG antibodies to SARS-CoV-2	Maryam Ranjbar	34965611	ELISA
Diagnostic accuracy of three SARS-CoV2 antibody detection assays, neutralizing effect and longevity of serum antibodies	Marina Bubonja-Šonje	33930473	ELISA,CLIA，LFIA

Evaluation of the performance of 25 SARS-CoV-2 serological rapid diagnostic tests using a reference panel of plasma specimens at the Uganda Virus Research Institute	Tom Lutalo	34536612	ELISA
High-throughput immunoassays for SARS-CoV-2 – considerable differences in performance when comparing three methods	Oskar Ekelund	34053400	ELISA,CLIA， LFIA
erformance evaluation of the Simtomax® CoronaCheck rapid diagnostic test	P.J. Ducrest	33961910	ELISA
Performance of anti-SARS-CoV-2 antibody testing in asymptomatic or mild COVID-19 patients: A retrospective study in outbreak on a cruise ship	Norihito Kaku	34582459	ELISA， LFIA
Performance of five rapid serological tests in mild-diseased subjects using finger prick blood for exposure assessment to SARS-CoV-2	David Triest	34304089	ELISA
Performance of the EUROIMMUN Anti-SARS-CoV-2 ELISA Assay for detection of IgA and IgG antibodies in South Africa	Maemu P. Gededzha	34161348	ELISA
Serological Evaluation of Human Antibodies of the Immunoglobulin Class A and G Against SARS-CoV-2 in Serum Collected in Newfoundland and Labrador	Robert Needle	33739895	ELISA,CLIA
A Reliable Indirect ELISA Protocol for Detection of Human Antibodies Directed to SARS-CoV-2 NP Protein	Arwa A. Faizo	34063315	ELISA,CLIA
Development and Evaluation of a Set of Spike and Receptor Binding Domain-Based Enzyme-Linked Immunosorbent Assays for SARS-CoV-2 Serological Testing	Rosa Camacho-Sandoval	34441440	ELISA
Development of an Enzyme-Linked Immunosorbent Assay (ELISA) for Accurate and Prompt Coronavirus Disease 2019 (COVID-19) Diagnosis Using the Rational Selection of Serological Biomarkers	Theano Lagousi	34829317	ELISA

Evaluation of three commercial SARS-CoV-2 serology assays in a tertiary care hospital in the United Arab Emirates	Adnan Alatoom	34119842	ELISA,CLIA
Validation of Commercial SARS-CoV-2 Immunoassays in a Nigerian Population	Fehintola Ige	34612691	ELISA
Quantitative measurement of IgG to SARS-CoV-2 antigens using monoclonal antibody-based enzyme-linked immunosorbent assays	Ingrid Sander	35127087	ELISA
Comparison of the Performance of 24 Severe Acute Respiratory Syndrome Coronavirus 2 Antibody Assays in the Diagnosis of Coronavirus Disease 2019 Patients	Shiji Wu	36003928	ELISA， LFIA
Evaluation of a Surrogate Enzyme-Linked Immunosorbent Assay–Based Severe Acute Respiratory Syndrome Coronavirus 2 (SARS-CoV-2) cPass Neutralization Antibody Detection Assay and Correlation With Immunoglobulin G Commercial Serology Assays	Vijayalakshmi Nandakumar	34181714	ELISA,CLIA
Multicentre Performance Evaluation of the Elecsys Anti-SARS-CoV-2 Immunoassay as an Aid in Determining Previous Exposure to SARS-CoV-2	Elena Riester	34368915	ELISA
Diagnostic Performance of Seven Commercial COVID-19 Serology Tests Available in South America	Ismar A. Rivera-Olivero	35252025	ELISA， LFIA
Evaluation of 11 SARS-CoV-2 antibody tests by using samples from patients with defined IgG antibody titers	Nina Lagerqvist	33828214	ELISA
Sensitive and specific serological ELISA for the detection of SARS-CoV-2 infections	Ji Luo	35305688	ELISA
Evaluation of 6 Commercial SARS-CoV-2 Serology Assays Detecting Different Antibodies for Clinical Testing and Serosurveillance	Suellen Nicholson	34258311	ELISA

Clinical and analytical evaluation of the Abbott AdviseDx quantitative SARS-CoV-2 IgG assay and comparison with two other serological tests	Gabriel N Maine	35181288	CLIA
Combination of a SARS-CoV-2 IgG Assay and RT-PCR for Improved COVID-19 Diagnosis	Kotaro Aoki	34108284	CLIA
Performance of an automated chemiluminescent immunoassay for SARS-COV-2 IgM and head-to-head comparison of Abbott and Roche COVID-19 antibody assays	Lau CS	33937471	CLIA
Performance verification of the Abbott SARS-CoV-2 test for qualitative detection of IgG in Cali, Colombia	Maria del Mar Castro	34469472	CLIA
Evaluation of serological lateral flow assays for severe acute respiratory syndrome coronavirus-2	Bianca A. Trombetta	34134647	LFIA
Rapid Serological Tests for SARS-CoV-2: Diagnostic Performance of 4 Commercial Assays	Sérgio M. de Almeida	33887722	LFIA
Analytical Validation and Clinical Application of Rapid Serological Tests for SARS-CoV-2 Suitable for Large-Scale Screening	Amedeo De Nicolò	34065954	LFIA
Clinical Sensitivity, Specificity and Epidemiology of SARS-CoV-2 Serological Testing Using the Biozek COVID-19 Test	Dennis Souverein	35054226	LFIA
Twelve lateral flow immunoassays (LFAs) to detect SARS-CoV-2 antibodies	Sophie I.Owen	34906597	LFIA

37. Supplementtary table S3

ELISA			
country	continent	sensitivity	specificity
Australia	Oceania	0.53	0.8
Austria	Europe	0.5	0.94
Bangladesh	Asia	0.95	0.97
Belgium	Europe	0.73	0.98
Brazil	South America	0.86	0.73
China	Asia	0.94	0.91
Denmark	Europe	0.85	0.99
Ecuador	Latin America	0.94	1.00
France	Europe	0.87	0.94
Germany	Europe	0.89	1.00
Israel	Asia	0.69	0.99
Italy	Europe	0.89	0.98
Netherlands	Europe	0.67	0.98
New Zealand	Oceania	0.73	1.00
Saudi Arabia	Asia	0.73	0.96
Serbia	Europe	0.94	0.98
Spain	Europe	0.86	0.99
Switzerland	Europe	0.75	0.93
Thailand	Asia	0.81	0.98
UK	Europe	0.86	0.96

USA	North America	0.85	0.98
Kenya	Eastern Africa	0.94	0.99
Iran	Asia	0.92	0.98
Croatia	Europe	0.85	1.00
Uganda	Eastern Africa	0.85	0.92
Sweden	Europe	0.92	0.99
Japan	Asia	0.78	1.00
South Africa	South Africa	0.64	0.95
Canada	North America	1.00	0.98
Mexico	North America	0.99	0.98
Greece	Europe	0.81	0.94
United Arab Emirates	Asia	0.84	1.00
Nigerian	West Africa	0.71	1.00
Qatar	Asia	0.76	0.91
CLIA			
country	continent	sensitivity	specificity
Australia	Oceania	0.36	0.98
Austria	Europe	0.93	1.00
Belgium	Europe	0.52	0.80
Brazil	South America	0.57	0.87
China	Asia	0.71	0.95
Finland	Europe	0.80	0.95
France	Europe	0.75	0.99
Germany	Europe	0.42	0.98
Italy	Europe	0.15	0.92

Japan	Asia	0.82	0.97
Liechtenstein	Europe	0.96	1.00
Singapore	Asia	0.25	0.99
Switzerland	Europe	0.21	0.96
UK	Europe	0.95	0.98
USA	North America	0.77	0.99
Croatia	Europe	0.97	0.99
Sweden	Europe	0.81	0.99
Colombia	Latin America	0.83	1.00
Canada	North America	0.96	0.98
United Arab Emirates	Asia	0.84	1.00
LFIA			
country	continent	sensitivity	specificity
Australia	Oceania	0.66	1.00
Belgium	Europe	0.65	0.97
China	Asia	0.79	0.94
Frence	Europe	0.77	0.97
Germany	Europe	0.93	0.97
Italy	Europe	0.63	0.96
Spain	Europe	0.60	0.86
UK	Europe	0.82	0.90
USA	North America	0.64	0.97
India	Asia	0.92	1.00
Japan	Asia	0.55	0.99
Korea	Asia	0.93	0.85

Santiago. Chile	Latin America	0.58	0.99
Croatia	Europe	0.88	1.00
Sweden	Europe	0.72	0.97
Brazil	South America	0.80	1.00
Netherlands	Europe	0.55	0.99
